# Synergistic blending of high-valued heterocycles inhibits growth of *Plasmodium falciparum* in culture and *P. berghei* infection in mouse model

**DOI:** 10.1038/s41598-017-06097-z

**Published:** 2017-07-27

**Authors:** Prashant Kumar, Angela O. Achieng, Vinoth Rajendran, Prahlad C. Ghosh, Brajendra K. Singh, Manmeet Rawat, Douglas J. Perkins, Prakasha Kempaiah, Brijesh Rathi

**Affiliations:** 1 Bio-Organic Research Laboratory, Department of Chemistry, University of Delhi, Delhi, 110007 India; 2 Department of Internal Medicine, Center for Global Health, University of New Mexico Health Sciences Center, Albuquerque, NM United States of America; 3 Department of Biomedical Sciences and Technology, School of Public Health and Community Development, Maseno University, Maseno, Kenya; 4 Department of Biochemistry, University of Delhi South Campus, New Delhi, 110021 India; 5 Department of Chemistry, Hansraj College University Enclave, University of Delhi, Delhi, 110007 India; 6 Department of Chemistry, Massachusetts Institute of Technology, Cambridge, MA-02139 United States of America

**Keywords:** Structure-based drug design, Drug discovery and development

## Abstract

A series of phthalimide analogues, novelized with high-valued bioactive scaffolds was synthesized by means of click-chemistry under non-conventional microwave heating and evaluated as noteworthy growth inhibitors of *Plasmodium falciparum* (3D7 and W2) in culture. Analogues **6a**, **6h** and **6 u** showed highest activity to inhibit the growth of the parasite with IC_50_ values in submicromolar range. Structure-activity correlation indicated the necessity of unsubstituted triazoles and leucine linker to obtain maximal growth inhibition of the parasite. Notably, phthalimide 6a and 6u selectively inhibited the ring-stage growth and parasite maturation. On other hand, phthalimide **6h** displayed selective schizonticidal activity. Besides, they displayed synergistic interactions with chloroquine and dihydroartemisinin against parasite. Additional *in vivo* experiments using *P. berghei* infected mice showed that administration of **6h** and **6u** alone, as well as in combination with dihydroartemisinin, substantially reduced the parasite load. The high antimalarial activity of **6h** and **6u**, coupled with low toxicity advocate their potential role as novel antimalarial agents, either as standalone or combination therapies.

## Introduction

Malaria is a devastating infectious disease in humans, causing ~214 million clinical cases globally with 438,000 deaths per annum^1^. Severe complications and mortality results primarily from infection with *Plasmodium falciparum*, which predominates in Africa. Over the last 15 years, several initiatives, including insecticide-treated bed nets, insecticide sprays and artemisinin-based combination therapies (ACT) led to reduced lethality of malaria (~4% per year) with a 40% reduction in clinical malaria cases between 2000 and 2015^2–4^. However, significant challenges remain including drug resistance, prolonged duration of infection in the human host^5^, high cost of anti-malarial drugs and lack of development for novel antimalarial drugs with potent activity^6^. Current frontline treatments for *P. falciparum* are based on ACT, which involve administration of artemisinin derivatives in combination with effective secondary agents, such as mefloquine, lumefantrine and piperaquine. The emergence of drug-resistance to malaria drugs, including the most reliable artemisinin-based therapies, has become a major global concern for controlling malaria, particularly in several countries of Southeast Asia^7–13^. The drug resistance coupled with the demand of a newly accepted set of antimalarial target product profiles has prompted the search for new inexpensive and stable antimalarials with novel modes of action that can be implemented for the treatment of all malaria species.

Phthalimide (Pht) skeleton is an imperative nucleus for various bioactive molecules^14–17^, starting material for alkaloids, pharmacophores^18, 19^ and antimalarials^20^. We also recently reported Pht analogues tailored with cyclic amines as moderate inhibitors of *P. falciparum* ^21, 22^. The presence of additional high-valued bioactive heterocycles may intensify the efficacy of the Phts. The broad spectrum pharmacological properties^23–25^ and antimalarial activities^26–29^ of benzimidazole and triazole heterocycles created the interest in the unification of these examined scaffolds into a single molecule. As a part of our ongoing efforts and diverse therapeutic efficiency of these heterocycles, we report here the design of synergistic association of Pht, benzimidazole and flexible triazoles anticipating new analogues as new entry for antimalarial chemotherapy. Click reactions under non-conventional microwave heating created new 31 Pht analogues (**6a**–**6e′**) and one representative analogue was characterized by single crystal X-ray crystallography. All the listed analogues were screened against chloroquine sensitive (3D7) and resistant (W2) strains of *P. falciparum* in culture and the lead molecules **6h** and **6u** displayed strong multi-stage (i.e. ring stage and trophozoite stages) antimalarial activity in submicromolar range. The top three Pht analogues **6a**, **6h** and **6u** were also examined as combination regimens with CQ and DHA. *In vivo* experiments carried out for **6h** and **6u** on a murine model of malaria (*P*. *berghei*) also suggested their candidature as antimalarial agents. In summary, efforts aimed at generating new antimalarial entries based on Phts was achieved through key structural variations that included the addition of benzimidazole and flexible triazoles.

## Results and Discussion

### Compound Design and Synthesis

**Figure 1:**
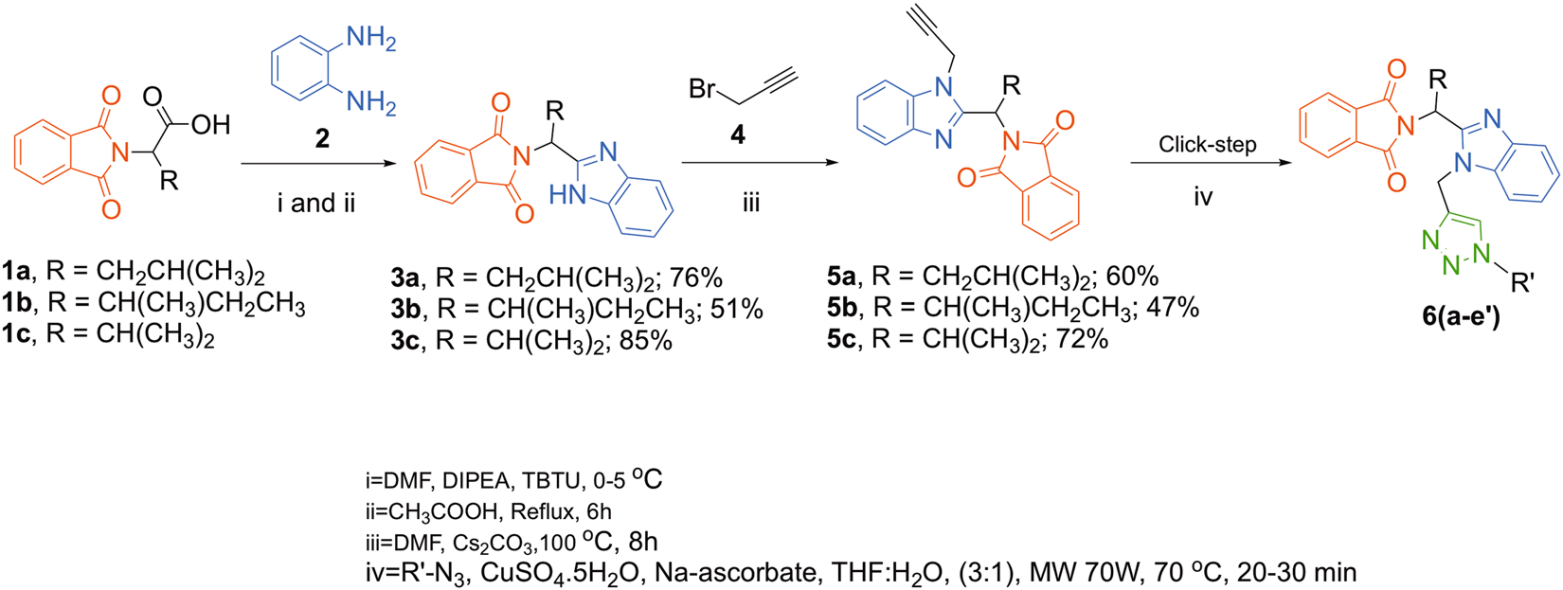
Synthesis of Pht analogues **6a**–**e′**.

We devised a chemical strategy that promoted the valuable fusion of Pht, benzimidazole and triazole. Numerous alterations on triazole scaffold were attempted, including substituted aromatic rings, alkyl chains and hydrophilic substituents to improve the activity profile. Amino acids with aliphatic chains i.e. valine, leucine and isoleucine were used as linkers. A simple and rationally compatible synthetic route was designed (Fig. 1) to build the library of new Pht analogues. Synthesis of the benzimdazole linked Pht, a key synthon began with the coupling between *N*-substituted isoindoline-1,3-dione and *o*-phenylenediamine, **2** followed by acetic acid catalyzed cyclization at refluxed temperature to furnish intermediates **3a–c**. The alkyne intermediate **5a** was prepared by the alkylation of **3a** with propargylic bromide (**4**). Initially, compound **5a** showed only 20% yield, however, efforts to standardize the reaction conditions resulted in a 62% yield. Likewise, compounds **5b** and **5c** were isolated in 47% and 72% yields, respectively.

**Figure 2:**
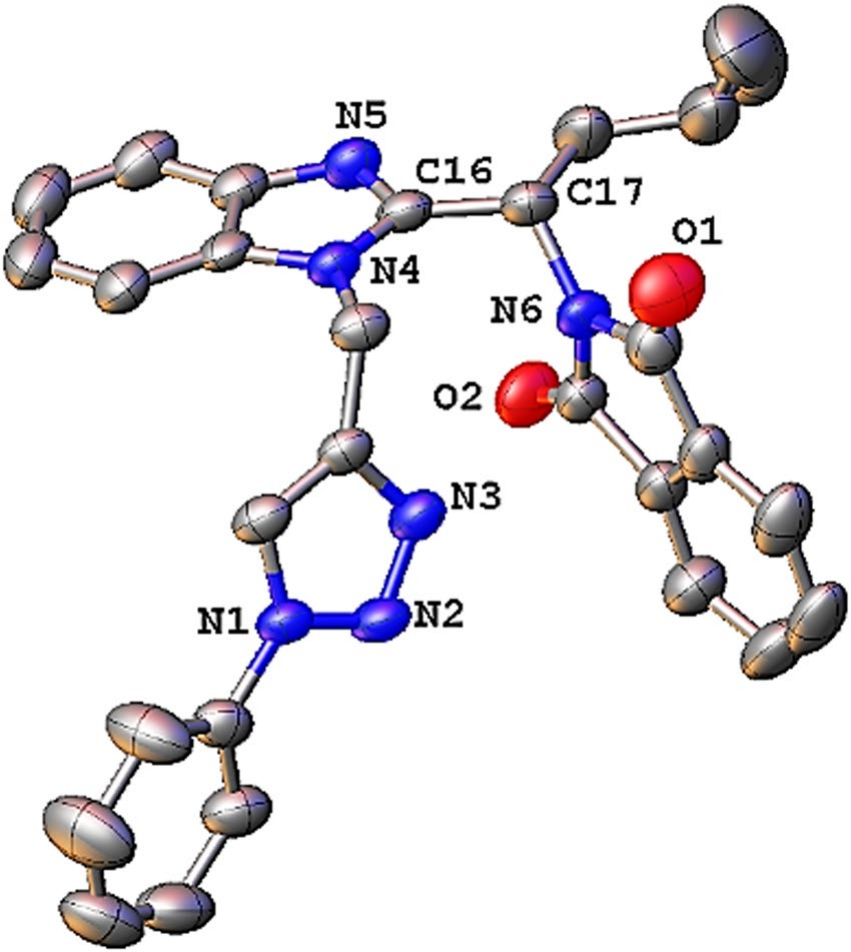
Molecular structure of **6a** at 40% probability level.

Subsequently, the alkylated products **5a–c** were treated with various azides (click reactions) under microwave irradiations to acquire the final products **6a**–**6e**′. The scope of various azides for synthesis of new Pht analogues is shown in Table S1. All the synthesized Pht analogues were characterized with various spectroscopic techniques (^1^H NMR, ^13^C NMR, HRMS and IR spectroscopy, etc.). In addition, single crystals were grown for one representative analogue **6a** and subjected to single crystal X-ray diffraction. The molecular diagram of **6a** is depicted in Fig. 2.

Compound **6a** crystallized in the monoclinic system with a *C2*/*c* space group. The details of data collection, structure solution and refinement are listed in Table S2.

### Biological Studies and Structure-Activity relationships (SARs) Analysis

Antimalarial activity of all the listed Pht analogues was initially assessed on asynchronous cultures of *P. falciparum* 3D7 (*Pf*3D7) clone using the SYBR Green assay. The most active compounds were those with a mean 50% growth inhibitory concentration (IC_50_) < 5.14 µM, set as reference cut-off IC_50_ value based on the Pht (**I**) reference molecule mean IC_50._ The mean IC_50_ values are based on three separate experiments (Table 1). Four new analogues **6a**, **6 h**, **6 m** and **6 u** displayed antiplasmodial activity against the *Pf*3D7 strain with IC_50_ < 5.14 µM. In addition, all four compounds inhibited the growth of CQ resistant *Pf*W2 strain with IC_50_ concentrations below the reference cut-off IC_50_; **6a** IC_50_ = 0.7 (±0.01) µM, **6 h** IC_50_ = 1.3 (±0.11) µM, **6 m** IC_50_ = 3.8 (±0.34) µM and **6 u** IC_50_ = 0.9 (±0.6) µM. Twelve out of the 31 Pht analogues lacked a dose-dependent effect on parasite growth, hence the IC_50_ was unattainable (no dose response, NDR). CQ and DHA were tested alongside the Phts as quality assurance and control of the assay, and SYBR Green derived IC_50_ values for both standard antimalarials were as recommended by literature^30^.

**Table 1:** SAR Study of R′ Substituent: Aromatic Rings. Note: Illustration of Pht analogues, Pht (I), and chloroquine and dihydroartemisinin IC_50_ values.

Entry	Compound	R	R′	IC_50_ (SE) µM	IC_50_ (SE) µg/mL
1	**6a**	CH_2_CH(CH_3_)_2_		0.9 (±0.14)	0.7 (± 0.03)
2	**6b**	CH_2_CH(CH_3_)_2_		NDR	NDR
3	**6c**	CH_2_CH(CH_3_)_2_		17.3 (± 0.0)	8.8 (± 0.0)
4	**6d**	CH_2_CH(CH_3_)_2_		NDR	NDR
5	**6e**	CH_2_CH(CH_3_)_2_		25 (± 1.5)	13 (± 0.8)
6	**6f**	CH(CH_3_)CH_2_CH_3_		NDR	NDR
7	**6g**	CH(CH_3_)CH_2_CH_3_		NDR	NDR
8	**6h**	CH(CH_3_)CH_2_CH_3_		0.9 (± 0.0)	0.7 (± 0.0)
9	**6i**	CH(CH_3_)CH_2_CH_3_		40 (± 6.2)	22.5 (± 2.9)
10	**6j**	CH(CH_3_)CH_2_CH_3_		73 (± 0.0)	33.4 (± 0.0)
11	**6k**	CH(CH_3_)_2_		30.86 (±0.0)	16 (± 0.0)
12	**6l**	CH(CH_3_)_2_		NDR	NDR
13	**6m**	CH(CH_3_)_2_		3.5 (± 2.9)	1.8 (± 1.3)
14	**6n**	CH(CH_3_)_2_		9.6 (± 0.0)	5.5 (±0.0)
15	**6o**	CH(CH_3_)_2_		23.5 (±11.9)	17.5 (±5.7)
16	**I**			5.14 (±1.67)	0.8 (±0.25)
17	CQ			0.03 (±0.67)	0.015 (±0.33)
18	DHA			0.003 (±0.3)	0.0017 (±0.16)

IC_50_ value < 5.14 µM: active and IC_50_ value>5.14 µM: inactive.

The structure-activity relationship is centred at the various substitutions of triazole ring and amino acids (Tables 1 and 2). Functionalized Phts were synthesized and screened for antimalarial activity against cultured *Pf*3D7 and compared with unsubstituted Pht **I** (IC_50_ = 5.14 ± 1.67 μM), CQ (IC_50_ = 0.03 ± 0.67 μM) and DHA (IC_50_ = 0.003 ± 0.3 μM). During the design of the new molecules Pht, benzimidazole scaffolds were kept constant and its flanking sides, triazoles were diversified. It is evident from the screening results (Tables 1 and 2) that the variations of R or R′ substituents play an important role in the potency of the compounds against growth of the parasite.

**Table 2:** SAR of R′ Substituent Antimalarial Activity: Aliphatic and Glycoside Groups.

Entry	Compound	R	R′	IC_50_ (SE) µM	IC_50_ (SE) µg/mL
**1**	**6p**	CH_2_CH(CH_3_)_2_		62 (±2.5)	31 (±1.2)
**2**	**6q**	CH_2_CH(CH_3_)_2_		8.4 (±0.5)	4.1 (±0.3)
**3**	**6r**	CH_2_CH(CH_3_)_2_		7 (±0.7)	3.5 (±0.34)
**4**	**6s**	CH_2_CH(CH_3_)_2_		No IC_50_	No IC_50_
**5**	**6t**	CH_2_CH(CH_3_)_2_		28 (±0.7)	14 (±0.3)
**6**	**6u**	CH_2_CH(CH_3_)_2_	H	0.7 (±0.0)	0.3 (±0.0)
**7**	**6v**	CH(CH_3_)CH_2_CH_3_		No IC_50_	No IC_50_
**8**	**6w**	CH(CH_3_)CH_2_CH_3_		15.6 (± 0.0)	11.6 (± 0.0)
**9**	**6x**	CH(CH_3_)CH_2_CH_3_		No IC_50_	No IC_50_
**10**	**6y**	CH(CH_3_)CH_2_CH_3_		No IC_50_	No IC_50_
**11**	**6z**	CH(CH_3_)CH_2_CH_3_	H	No IC_50_	No IC_50_
**12**	**6a′**	CH(CH_3_)_2_		No IC_50_	No IC_50_
**13**	**6b′**	CH(CH_3_)_2_		7.2 (±0.0)	3.8 (±0.0)
**14**	**6c′**	CH(CH_3_)_2_		No IC_50_	No IC_50_
**15**	**6d′**	CH(CH_3_)_2_		48.3 (±0.6)	20 (±0.29)
**16**	**6e′**	CH(CH_3_)_2_		16.7 (±21)	6.9 (±10.1)

The antimalarial data demonstrates that the introduction of larger R groups (isobutyl or *sec*-butyl) increases the potency as noticed in case of Phts **6a**, **6h** and **6u** whereas relatively smaller groups lower the activity (i.e. **6m**). As shown in Table 1, the insertion of a phenyl substituent also influences the potency of the molecules. Pht analogues possessing 4-fluorophenyl ring on the triazoles moiety with R represented as butyl group (**6h**) was noticed as more active over the ananlogues containing substituted phenyl group at triazole moiety. This result, at least in part, appears to be due to the high electronegativity of fluoro group. Analogues with an unsubstituted aromatic ring also exhibited significant inhibition of the parasite growth, but only when R was replaced with an isobutyl group (e.g. **6a**). In the absence of a functional group on the triazole ring with R represented as an isobutyl group, we observed the highest potency against the *P. falciparum* 3D7 strain (i.e. **6u**).

#### *Stage-Specificity and Effects on Parasitemia Titres*

**Figure 3:**
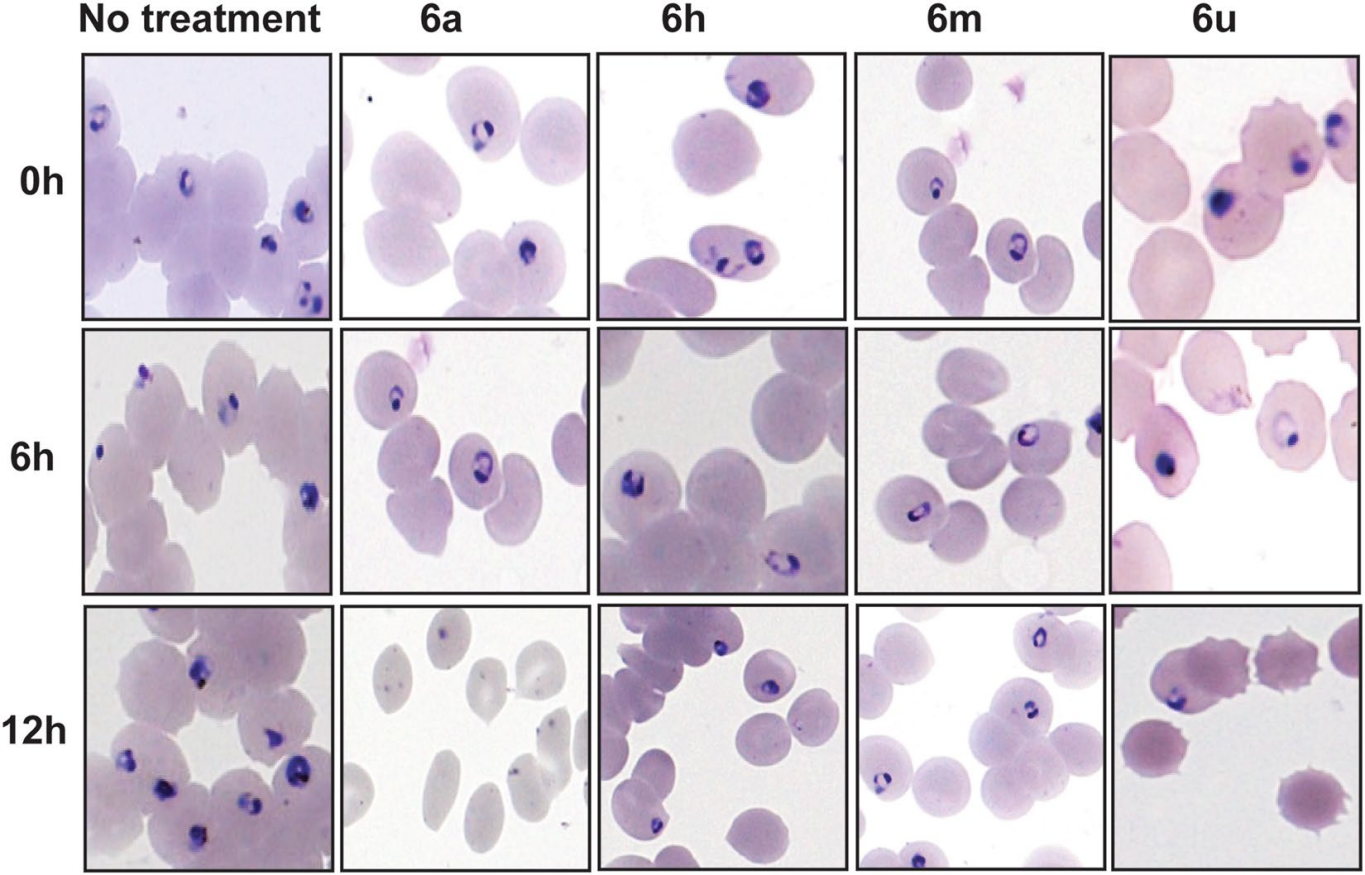
Micrographs of *P. falciparum* ring-stage forms treated with Pht analogues. Illustration of Pht analogues treatment effect on early erythrocytic parasite stage (rings). (All treatments were performed in parallel to a no treatment group).

**Figure 4:**
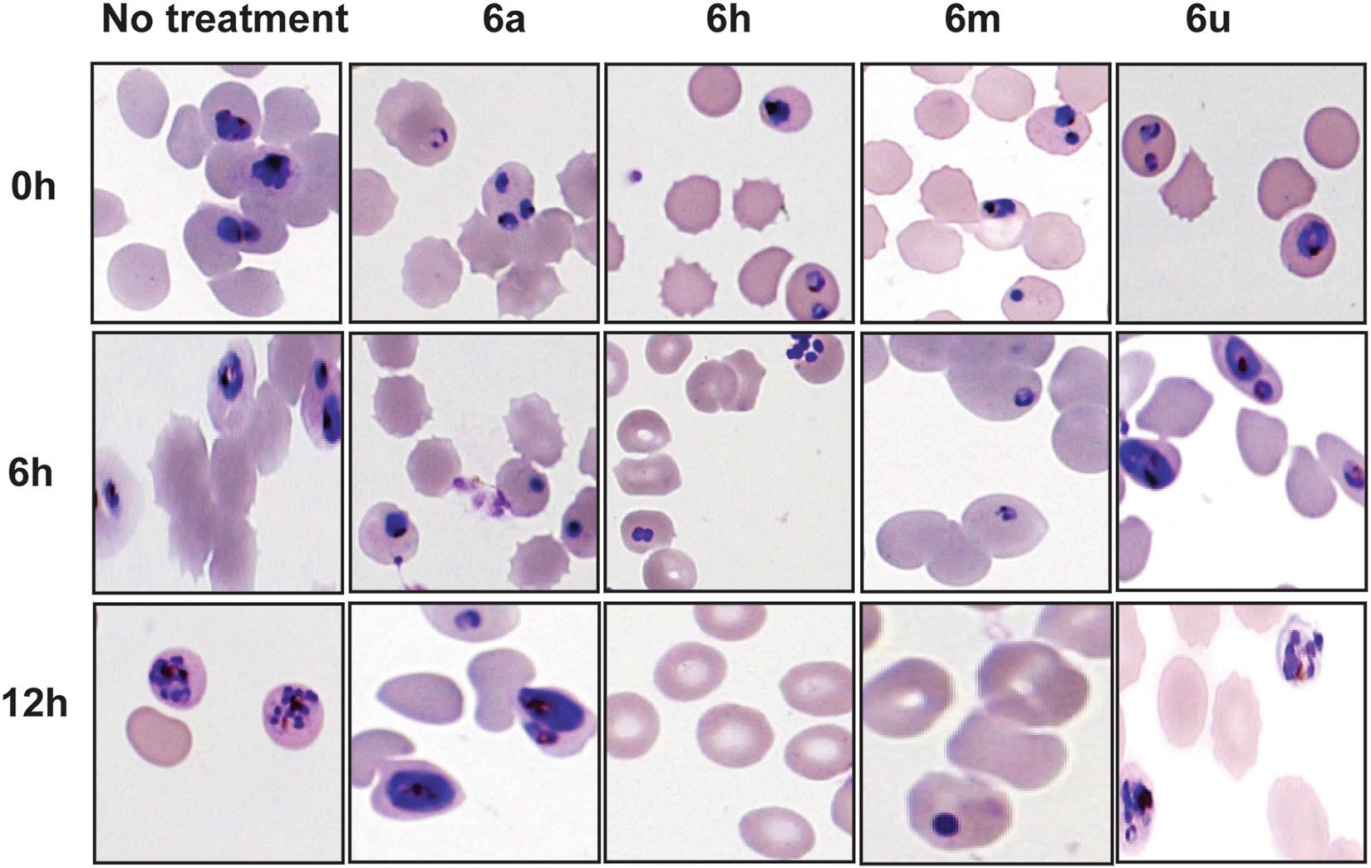
Micrographs of *P. falciparum* trophozoite stages treated with Pht analogues. Note: Illustration of Pht analogues treatment effect on early (rings) and mature (trophozoites and schizonts) parasite blood stages. (All treatments were performed in parallel to a no treatment group).

Next, we sought to determine stage-specificity of the antiplasmodial activity of the four active analogues (**6a**, **6h**, **6m** and **6u**) on synchronized *Pf*3D7 strain cultures at 2% hematocrit and 1% parasitemia, with concentrations corresponding to individual drug IC_50_. To determine the effect of the compounds on both early and late ring stage parasites, the treatment was conducted on newly synchronized rings for 12 hours, and drug effect on parasite growth and morphology was monitored at 6 and 12 hours after exposure. Similarly, early trophozoites were exposed to each compound and incubated for 16 hours, and monitored at 6 and 16 hours post-exposure. The effect of the compounds on parasite morphology and development was compared in exposed and unexposed drug wells (Figs 3 and 4). Compounds **6a** and **6u** were active against ring-stage forms as indicated by a marked reduction in parasite density and abnormal ring stage morphology at 6 hour and 12 hour post-exposure. However, compounds **6a** and **6u** did not interfere with development of parasite progression from trophozoites to schizonts when they were exposed to a separate culture of trophozoites. Treatment with **6h** and **6m** did not arrest ring-stage maturation as indicated by the marked increase in ring-stage growth at 12 hours, like the no treatment group. However, upon examination of both **6h** and **6m** treatment on mature blood-stages, **6h** resulted in complete destruction of trophozoites at 6 hours after drug exposure of early-stage trophozoites. Further, monitoring the effect of **6h** at 16 hours showed the presence of schizonts with abnormal morphology.

**Figure 5:**
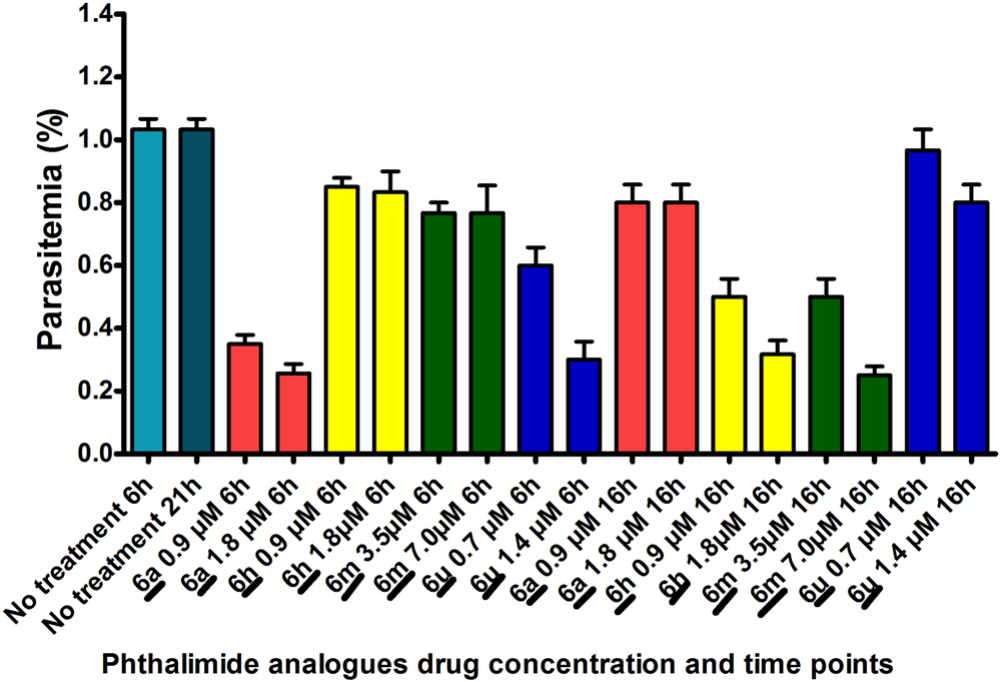
Effect of Pht analogues on parasite growth titres (Note: Graphical description of the inhibitory effect of select analogues on parasite growth titres 6 hours after incubation with ring-stages and 16 hours after drug incubation with trophozoite stage. Parasitemia percentage was derived by counting the number of infected erythrocytes from a total of 2,000 erythrocytes on Giemsa stained thin smears from each experiment. Bar diagrams represent the average of three different experiments).

Although, trophozoite growth did not appear to be affected by treatment with **6m** at 6 hours, the resultant schizonts appeared less granular and lacked distinguishable merozoites upon 16 hours exposure. The effect on the analogues on parasitemia counts was correlated with their stage-specificity. Analogues **6a** and **6u** caused a reduction in ring stage parasitemia at 6 hrs post exposure, while their effect on mature blood stage parasite titres at 16 hours was negligible (Fig. 5). Although, **6h** and **6m** did not affect parasite growth at 6 hours post exposure (i.e., ring-stage), both analogues caused a reduction in parasitemia at 16 hours (i.e., trophozoite stage, Fig. 5).

### Drug-Drug Interaction Assays

We then explored the synergistic drug inhibitory activities between the Pht analogues and CQ or DHA. Synergistic inhibitory activities were observed between three analogues (**6a**, **6h** and **6u**) in combination with both CQ and DHA against the 3D7 and W2 strains (Table S3). The determination of drug interactions between the analogues with CQ and DHA is necessary for identifying possible partner drugs to combat resistance to current antimalarial therapies. Recent reports on the emergence of CQ sensitive *P. falciparum* strains achieved in some malaria endemic regions is attributed to withdrawal of CQ pressure on parasite populations due to replacement with sulfadoxine-pyrimethamine (SP) and ACTs^31, 32^. CQ was an ideal antimalarial due to its pharmacokinetics, safety profile and low cost. Since *P. falciparum* resistance developed primarily because of administration as a monotherapy, identifying potential CQ partner drugs can beneficial in reducing development of historical parasite resistance to CQ monotherapy. Artemisinin (ART) and its derivatives are the current front line of defense against uncomplicated *P. falciparum* malaria and are administered as ACTs. Emergence of resistance to both ACT drug components warrants identification of ART replacement and possible combination chemotypes^33–35^. Notably, three Pht analogues (**6a**, **6h** and **6u**) showed synergistic activity when combined with CQ and DHA against both *Pf*3D7 and *Pf*W2, suggesting their potential for use in combination therapies.

### Antimalarial Effect of Pht Analogs Alone and in Combination with Artemisinin in Plasmodium berghei Infected Mice

**Figure 6:**
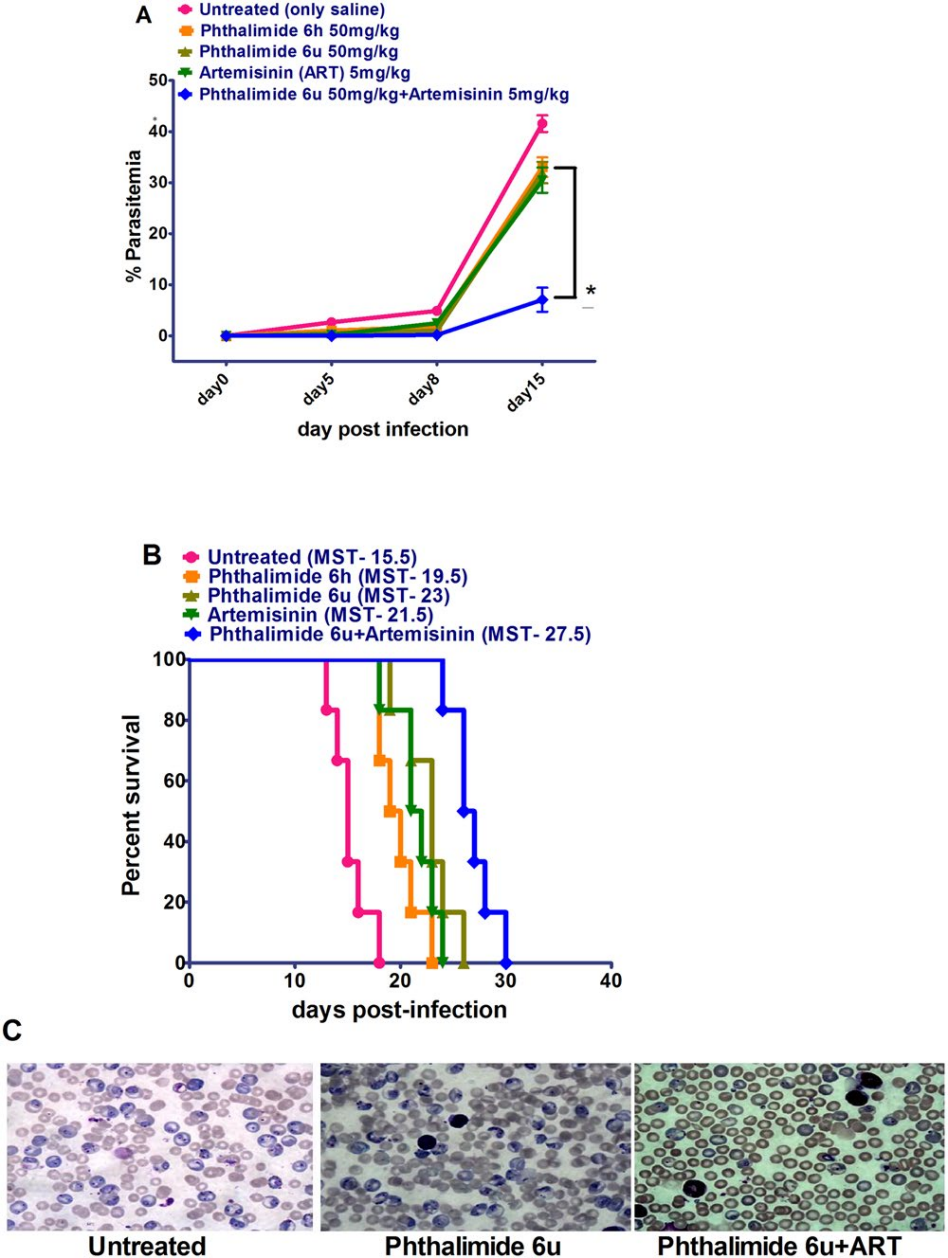
Antimalarial effect of Pht analogs (**6h** and **6u**) alone and in combination with Artemisinin (**6u** and ART) on parasitemia and survival in mice infected with *P. berghei* NK65. Mice were injected with 1 × 10^7^ *P. berghei* infected RBCs. After 48 hours mice were treated with control (PBS**:** untreated**), 6h** and **6u** (alone), and **6u** in combination with artemisinin (all injected subcutaneously) for four consecutive days. The % of parasitemia was determined for the groups on days 5, 8 and 15 by randomly selecting 10 different optical fields on blood smears. (**A**) Administration of **6h** and **6u** (alone), and **6 u** in combination with ART. Data are the mean ± SEM from six animals per treatment group. (**B**) Survival in the treatment groups (n = 6/group). (**C**) Photomicrograph of blood smears of untreated versus treatment groups at day 15 post infection (100x magnification). ART, Artemisinin; *P < 0.05.

The antimalarial effect of two active analogues, **6h** and **6u** (administered at 50 mg/kg of body weight), was determined in mice infected with *P. berghei* NK65, a strain, which results in high levels of blood-stage parasitemia. Administration of either **6h** or **6u** alone for four consecutive days caused suppression of the parasite load on days 5 and 8 of infection and improved survival as compared to untreated (Fig. 6A and B). However, the **6u** analogue had better antimalarial efficacy than the **6h** analogue (Fig. 6A). As such, we then evaluated the efficacy of **6u** (50 mg/kg) in combination with artemisinin (5 mg/kg of body weight) at reduced dosage in the murine malaria model. As shown in Fig. 6, neither of the compounds delivered as monotherapy conferred clearance of parasitemia, however, co-administration of the **6u** analogue with ART considerably enhanced the antimalarial efficacy by reducing the parasite load and extending survival (*P* < 0.05, Fig. 6A and B). The median survival times of animals treated with Pht **6u** alone, as well in combination with ART were 23 and 27.5 days, respectively (*P*<0.001). These results demonstrate the compound **6u** in combination with ART has the greatest therapeutic efficacy in the murine model of malaria.

#### Cytotoxicity Evaluation

As a final step in the investigational pipeline, we determined the cytotoxic effect of the four active analogues (**6a**, **6h**, **6m** and **6u**) in U937 cell lines by measuring 50% cytotoxic concentrations (CC_50_) and calculating selectivity indices (SIs) as a measure of toxicity in human cells.

Compounds **6a** and **6m** displayed potent CC_50_ values <1 µM of 0.91 ± 0.32 µM and 0.78 ± 0.32 µM, respectively, and were considered to possess higher selectivity for U937 cell lines vs. *P. falciparum* (SI values of 1.01 ± 1.5 µM and 1.11 ± 1.5 µM, respectively). Analogues **6h** and **6u** possessed less selectivity for U937 cell lines with CC_50_ values of 28.82 ± 0.67 µM and 2.08 ± 1.6 µM, respectively, and SI values of 41.2 ± 1.7 µM and 2.31 ± 0.76 μM, respectively, and were, therefore, considered less toxic to human cells. As such, additional chemical modifications of **6a** and **6m** may be required to produce analogues with less selectivity and toxicity for human cell lines in the context of retaining their antiplasmodial activity.

## Conclusion

In summary, we present simple and inexpensive chemical procedures with readily available starting materials considering the need for low-cost, novel antimalarial agents for use in malaria endemic areas. Results presented here investigated *in vitro* and *in vivo* antimalarial activity of novel Pht analogues blended with benzimidazole and triazoles, which have been synthesized by means of click reactions under microwave conditions. Amongst all active members, eight analogues displayed growth inhibition of *P. falciparum* in culture. The synergy of the lead compounds **6h** and **6u** with standard antimalarial drugs such as ART, demonstrate their suitability as combination regiments. Future studies will focus on lead optimization, pharmacokinetics and parasite target site(s) to advance Pht analogues as potential antimalarial candidates for clinical use.

## Methods

### Chemistry

Solvents and reagents were purchased from commercial sources and used without purification for the experiments. Homogeneity/purity of all the products was assayed by thin-layer chromatography (TLC) on alumina-coated plates (Merck). Product samples in chloroform (CHCl_3_) were loaded on TLC plates and developed in Ethyl acetate/Petroleum ether (1:1, v/v). When slight impurities were detected by iodine vapour/UV light visualization, compounds were further purified by chromatography on silica gel columns (100–200 mesh size, CDH). Reactions using microwave were run in a closed vial applying a dedicated CEM-Discover monomode microwave apparatus operating at a frequency of 2.45 GHz with continuous irradiation power from 0–300 W (CEM Corporation, P.O. Box 200, Matthews, NC 28106). Melting points were determined on Melting point machine M-560 (Buchi). Infrared (IR) spectra were recorded in KBr medium using a Perkin-Elmer Fourier Transform-IR spectrometer, whereas ^1^H and ^13^C nuclear magnetic resonance (NMR) spectra were recorded in CDCl_3_ DMSO and D_2_O medium on a JEOL ECX-400P NMR at 400 MHz and 100 MHz, respectively at USIC, University of Delhi, using TMS as an internal standard. Absorption frequencies (ν) are expressed in cm^−1^, chemical shifts in ppm (δ-scale) and coupling constants (*J*) in Hz. Splitting patterns are described as singlet (s), doublet (d), doublet of doublet (dd), triplet (t), quartet (q) and multiplet (m). The high-resolution mass spectral data was obtained using a Agilent Technology-6530, Accurate mass, Q-TOF LCMS spectrometer at USIC, University of Delhi. Compounds **1a**–**1c** were prepared following literature procedures^36^.

### General Procedure for Synthesis of Compounds **3a**–**3c**

In first step, respective compounds **1a–1c** (38 mmol) were dissolved in 250 mL of DMF and DIPEA (45 mmol) was added drop-wise at 0–5 °C. After 10 minutes the essential amount of TBTU (45 mmol) was added slowly and the reaction contents were stirred for 30 minutes at the same temperature. Thereafter, *o*-phenylenediamine (38 mmole) was added and the resulting mixture was stirred at 0–5 °C for 6 hours. After completion of the reaction as confirmed by TLC, the reaction mixture was quenched with ice cold water, which resulted in precipitate formation. The precipitate was filtered off, washed with excess of ice cold water and dissolved in appropriate amount of ethyl acetate. The resulting organic phase was washed with 1 N HCl followed by saturated solution of NaHCO_3_ and at last with water. The separated ethyl acetate layer was dried over Na_2_SO_4_ and concentrated under reduced pressure to acquire the crude product. Next, the crude product was dissolved in 150 mL glacial acetic acid and the resulting suspension was refluxed for 6 hours. After completion of the reaction as confirmed by TLC, the reaction mixture was cooled to room temperature, concentrated under reduced pressure and diluted with ice cooled water. The resulting solid was filtered off and washed thoroughly with ice cold water and saturated NaHCO_3_ solution to obtain the desired products. The products were purified by silica gel column chromatography eluting with 20% mixture of ethyl acetate in n-hexane and the final compounds **3a–3c** were isolated.

### General Procedure for Synthesis of Compounds **5a**-**5c**

In a RB flask, respective compounds **3a**–**3c** (15 mmol), Cs_2_CO_3_ (45 mmol) and appropriate amount of DMF were mixed and the contents were heated at 100 °C for 20 minutes and subsequently, propargyl bromide (**4**) (22 mmol) was added drop wise. This resulted in a turbid reaction mixture, which was stirred at 100 °C for next 8 hours. After completion of the reaction as indicated by TLC, the reaction mixture was cooled to attain room temperature and concentrated under *vacuo* to give residue. Thereafter, ice cooled water was added to the residue and the resulting precipitate was filtered and dried. The residue was purified by silica gel column chromatography eluting with 10% mixture of ethyl acetate in n-hexane to afford the titled compounds **5a–5c**.

### General Synthetic Procedure of New Analogues **6a**-**6e’**

A microwave vial was charged with respective compounds **5a–5c** (1.34 mmol) and azide (2.02 mmol) and THF:H_2_O (4:1, v/v, 5 mL). The required amount of CuSO_4_.5H_2_O (0.27 mmol) and sodium ascorbate (0.54 mmol) was added and the vial was sealed tightly, which was heated at 70 °C under microwave irradiation of 70 W for 20 minutes. The progress of the reaction was confirmed by TLC. After completion of the reaction, the reaction mixture was transferred into a RF flask and concentrated under *vacuo* to give a residue that was quenched with ammonia solution and filtered off. Thus, obtained residue was purified by silica gel column chromatography eluting with appropriate mixture (~25%) of ethyl acetate in petroleum ether to afford the titled compounds **6a–6e′**. The spectral data of all new compounds is described in supporting information.

### Biological Evaluation

#### *In vitro* cultivation of P. falciparum

The laboratory-adapted *P. falciparum* strains 3D7 (Africa; CQ sensitive) and W2 (Africa; CQ resistant), were acquired from Malaria Research and Reference Reagent Resource Center (MR4) (Manassas, VA, USA) and maintained with type O +ve erythrocytes suspended in continuous complete culture medium as described^37^. Complete culture media consisted of 10.43 g/litre of RPMI 1640-HEPES supplemented with 10% (vol/vol) human AB serum, 92.6 mg/litre L-glutamine, 50 mg/liter hypoxanthine, 2 g/litre sodium bicarbonate (Sigma-Aldrich, St. Louis, MO). Incubation of cultures was at 37 °C and maintained in a low oxygen atmosphere (5% O_2,_ 5% CO_2_, 90% N). The levels of parasitemia in the cultures were maintained at between 2 and 10%, with 5% hematocrit. Media was changed after every 24 hours and parasitemia monitored every 48 hours. Synchronous cultures were prepared by sorbitol lysis^38^. Stock solutions of the Pht, Pht analogues, and DHA were prepared in absolute dimethyl sulfoxide (DMSO) while CQ was prepared in distilled water. The prepared drug stock solutions were used immediately or stored at −80 °C for not longer than one month before use. Stock solutions were further diluted in serum free RPMI 1640 media (stock solution solvent final conc. 0.05%) before performing a 2-fold serial dilution to achieve dose ranges of 0.2 µM to 100 µM (Pht and derivatives), 7.8 to 2,000 nM (CQ) and 0.17 to 87.5 nM (DHA). 25 μL of the drug diluents were aliquoted into 96 well plates and used immediately. Alternatively, the stock solutions were diluted in distilled water, 2-fold serial dilution performed on a 96 well plate and allowed to air dry in a laminar flow hood after which dried plates were stored at 4 °C until use. SYBR Green I assay technique with additional modification was used for drug susceptibility testing of the parasites. Parasite cultures of >1% parasitemia were diluted to 1% parasitemia and 2% hematocrit and 200 μL were transferred onto drug pre-dosed plates and incubated at 37 °C for 90 hours^39, 40^. Drug exposure was terminated by freezing the drug plates at −80 °C for 24 hours after which lysis buffer containing (per liter) 100 mM Tris-HCl, 10 mM EDTA, 0.016% Saponin, 1.6% triton X-100 and 20X SYBR Green I dye was added and the sample incubated for 2 hours in the dark. Relative fluorescence units (RFU) were read using the Perkin Elmer Wallac 1420 fluorescence plate reader, with excitation and emission wavelengths of 485 nm and 535 nm respectively. The readouts values were aligned with corresponding drug doses and analysis performed by Graph-pad Prism software. The drug concentrations (x value) were transformed using X = Log[X] and plotted against the counts (y values) and the data analysed by non-linear regression (sigmoid dose-response/variable slope equation) to yield the IC_50._

#### Drug interaction combination assays and analysis

A modified fixed-ratio isobologram method was used to assess interaction between CQ/DHA and Pht analogues^41^. Briefly, a total of 5 solutions containing fixed-ratio mixtures of Pht derivatives with either CQ or DHA were prepared in the following ratios: 1:1, 2:1, 3:2, 2:3, and 1:4. The starting concentrations for ten 2 fold-serial dilutions across the microtiter plate were assigned so that the IC_50_ of each drug would be in the 5^th^ serial dilution of the plate. Each drug was tested alone and at fixed ratios of its IC_50_. The assessment of drug interaction was based on the calculation of the fractional inhibitory concentration (FIC) of two drugs in the 4 combination ratios. FIC was calculated for each association by dividing the IC_50_ of the drug in the combination by the IC_50_ of the drug alone. The sum of these two FIC (∑FIC); (∑FIC = 1) indicates an additive effect between drug A and drug B, (∑FIC < 1), suggesting a synergistic effect and (∑FIC > 1) indicates antagonism.

#### Measurement of cytotoxic activity in U937 cells

Cytotoxicity of the 4 select Pht compounds on human cells was evaluated by assessing cell viability of the U937, a human acute monocyte leukemia cell lines by use of the MTT assay^42^. U937 was acquired from American Type Culture Collection (ATCC, Rockville, MD) and maintained in RPMI 1640 medium supplemented with 10% fetal bovine serum, and Penicillin-streptomycin (1% v/v) (Gibco, UK) at 37 °C. Robust U937 cells at a concentration of 80,000 cells/ml were plated into 96-well plates and incubated for 24 hours. Six concentrations of the Pht compounds were added in a two-fold dilution from starting concentration of 50 to 1.56 µM in triplicate and incubated with the cells for 24 hours. This was followed by addition of 10 µL MTT solution (5 mg/mL) into each well, incubated for 4 hour at 37 °C followed by addition of 50 µL DMSO to dissolve the formazan precipitate according to manufacturer’s protocol. Aliquots were drawn from each well and color intensity was measured spectrophotometrically in an ELISA plate reader (Biotek, ELx800) at 540 nm. The cell viability ratio was calculated by the following formula: % cytotoxicity = [Mean OD of test cells] − [Mean OD of control cells]/[Mean OD of control cells] × 100. Viability counts were then plotted against corresponding drug concentrations to yield cytotoxicity (CC_50_) through non-linear regression (sigmoid dose-response/variable slope equation). Drug CC_50_ values were then used to define selectivity indices, which is a measure of drug safety in human relative to parasitic cells and was calculated as the ratio of the CC_50_ value determined on the U937 cells (cytotoxicity) and the IC_50_ value determined on parasite growth inhibition 3D7 (anti-plasmodial activity). In this study, we set SI > 2 as cut-off as an indicative of low drug cytotoxicity.

#### *In vivo* experiments

All animal experiments were performed in female Swiss albino mice (4 to 5 weeks old, weighing 25 to 30 g). The animals were housed under standard controlled conditions at 25 °C with a 12-h light-dark cycle and access to sterilized food pellets and water. All experiments were carried out in accordance with the standard procedures approved by the Animal Ethics Committee of the University of Delhi South Campus, under the Control and Supervision of Experiments on Animals (CPCSEA), Ministry of Social Justice and Empowerment, Government of India. To examine the therapeutic efficacy of most potent Pht analogues 6h and 6u, a murine model of malaria was developed by intraperitoneal i.p. administration of standard inoculum of rodent strain of *P. berghei* Nk65 carrying 1 × 10^7^ parasitized erythrocytes per 200 µl volume to each experimental Swiss albino mouse. The antimalarial activity was carried out in accordance with a slightly modified version of the Peter’s 4-day suppressive test^43^. The animals were assigned to each group (n = 6). Subsequently, after 48 hour of postinfection, the parasitemia level reached 1 to 2%, and all the groups of mice were treated by subcutaneous (s.c.) injection with compounds **6h** and **6u** alone as well in combination with artemisinin solubilized in DMSO. One group was kept as a control and treated with physiological saline. The efficacy of the treatment was monitored by measuring the parasitemia and survival on days 5, 8 and 15 posttreatments by obtaining thin smears of blood withdrawn from the tail vein of infected mice and staining with 10% Giemsa. The level of parasitemia was determined by counting infected and noninfected erythrocytes from 10 to 15 randomly selected optical fields at 100x magnification and expressed as the number of infected erythrocytes per 100 erythrocytes. The survival of mice was recorded and observed for external symptoms, such as change in body weight, ruffled fur, lethargy and paralysis, until 30 or 40 days posttreatment. The reduction in the level of parasitemia was taken as the index for the curative activities of the drugs. The percentage of parasitemia was calculated manually with the Cell Counting Aid software^44^ using the formula (total no. of parasitized RBCs)/(total no. of RBCs) × 100.

### Statistical analysis

For *in-vivo* experiments, statistical differences between two groups were determined by Student’s t test and between multiple groups using one-way analysis of variance (ANOVA), with *P* values of <0.05, by GraphPad Prism (version 5.01; GraphPad Software, Inc., CA). The survival of the mice was followed up to day 30 or 40 postinfection using Kaplan-Meier survival analysis, and statistical differences in animal survival were analyzed by a log rank test.

## Electronic supplementary material

Supporting Information
