# Author Correction: Synergistic blending of high-valued heterocycles inhibits growth of *Plasmodium falciparum* in culture and *P. berghei* infection in mouse model

**DOI:** 10.1038/s41598-020-70608-8

**Published:** 2020-08-24

**Authors:** Prashant Kumar, Angela O. Achieng, Vinoth Rajendran, Prahlad C. Ghosh, Brajendra K. Singh, Manmeet Rawat, Douglas J. Perkins, Prakasha Kempaiah, Brijesh Rathi

**Affiliations:** 1 Bio-Organic Research Laboratory, Department of Chemistry, University of Delhi, Delhi, 110007 India; 2 Department of Internal Medicine, Center for Global Health, University of New Mexico Health Sciences Center, Albuquerque, NM USA; 3 Department of Biomedical Sciences and Technology, School of Public Health and Community Development, Maseno University, Maseno, Kenya; 4 Department of Biochemistry, University of Delhi South Campus, New Delhi, 110021 India; 5 Department of Chemistry, Hansraj College University Enclave, University of Delhi, Delhi, 110007 India; 6 Department of Chemistry, Massachusetts Institute of Technology, Cambridge, MA-02139 United States of America

Correction to: *Scientific Reports* 10.1038/s41598-017-06097-z, published online 27 July 2017

This Article contains an incorrect version of Figure 3 where identical images were used to represent different compounds’ anti-parasitic activities in images 6a/0h, 6m/12h, 6a/6h and 6m/0h. The correct version of Figure 3 appears below as Figure 1.

**Figure 1:**
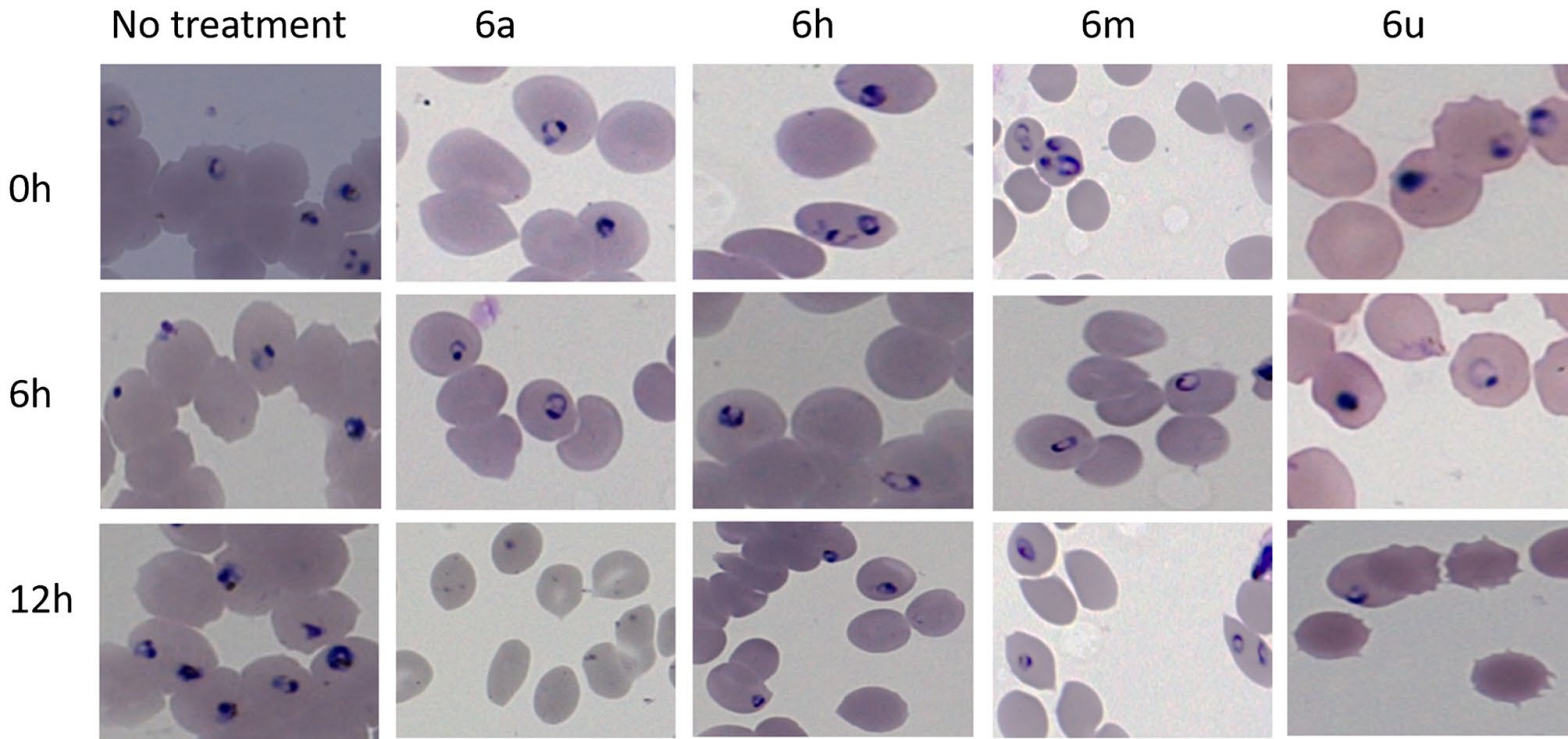
.

