# Supplementary material for: Synergistic blending of high-valued heterocycles inhibits growth of Plasmodium falciparum in culture and P. berghei infection in mouse model
Source: Sci Rep. 2017 Jul 27;7:6724. doi: 10.1038/s41598-017-06097-z (PMC5532363; doi:10.1038/s41598-017-06097-z)
Supplement: Supplementary file 1 — Supporting Information [file 41598_2017_6097_MOESM1_ESM.pdf]

Supporting Information

Synergistic blending of high-valued heterocycles inhibits growth of *Plasmodium falciparum* in culture and *P. berghei* infection in mouse model

Prashant Kumar[1], Angela O. Achieng[2,3], Vinoth Rajendran[4], Prahlad C. Ghosh[4], Brajendra K. Singh[1], Manmeet Rawat[2], Douglas J. Perkins[2], Prakasha Kempaiah[2*], Brijesh Rathi[5,6*]

[1]Bio-Organic Research Laboratory, Department of Chemistry, [5]Hansraj College University Enclave, University of Delhi, Delhi-110007 India

[2]Center for Global Health, Department of Internal Medicine, University of New Mexico Health Sciences Center, Albuquerque, NM, United States of America

[3]Department of Biomedical Sciences and Technology, School of Public Health and Community Development, Maseno University, Maseno, Kenya

[4]Department of Biochemistry, University of Delhi South Campus, New Delhi-110021 India

[6]Department of Chemistry, Massachusetts Institute of Technology, Cambridge, MA-02139 Unites States of America

***Corresponding Author**: BR (email: brijeshrathi@hrc.du.ac.in)

PK (email: Pkempaiah@salud.unm.edu)

Table S1. Scope of Various Azides for Synthesis of New Pht Analogues (**6a-6e'**).

Entry	Compound	R	R'	Isolated Yield (%)
1	**6a**	$CH_2CH(CH_3)_2$	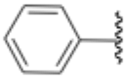	76
2	**6b**	$CH_2CH(CH_3)_2$	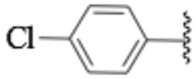	72
3	**6c**	$CH_2CH(CH_3)_2$	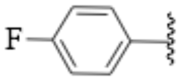	54
4	**6d**	$CH_2CH(CH_3)_2$	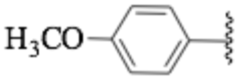	71
5	**6e**	$CH_2CH(CH_3)_2$	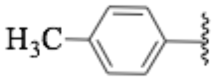	85
6	**6f**	$CH(CH_3)CH_2CH_3$	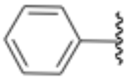	51
7	**6g**	$CH(CH_3)CH_2CH_3$	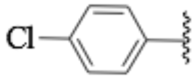	72
8	**6h**	$CH(CH_3)CH_2CH_3$	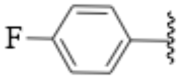	61
9	**6i**	$CH(CH_3)CH_2CH_3$	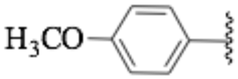	77
10	**6j**	$CH(CH_3)CH_2CH_3$	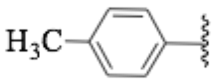	68

11	**6k**	$CH(CH_3)_2$	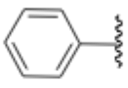	54
12	**6l**	$CH(CH_3)_2$	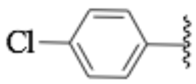	68
13	**6m**	$CH(CH_3)_2$	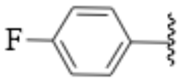	77
14	**6n**	$CH(CH_3)_2$	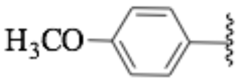	42
15	**6o**	$CH(CH_3)_2$	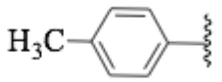	37
16	**6p**	$CH_2CH(CH_3)_2$	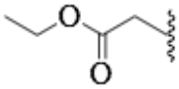	74
17	**6q**	$CH_2CH(CH_3)_2$	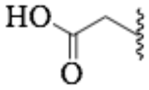	60
18	**6r**	$CH_2CH(CH_3)_2$	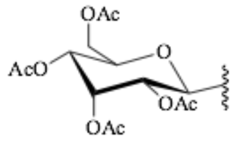	76
19	**6s**	$CH_2CH(CH_3)_2$	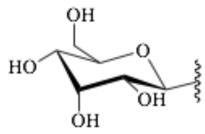	62
20	**6t**	$CH_2CH(CH_3)_2$	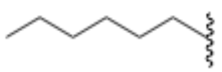	32
21	**6u**	$CH_2CH(CH_3)_2$	H	64
22	**6v**	$CH(CH_3)CH_2CH_3$	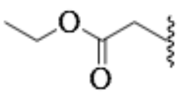	72

23	**6w**	$CH(CH_3)CH_2CH_3$	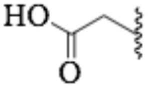	54
24	**6x**	$CH(CH_3)CH_2CH_3$	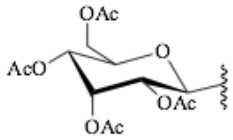	85
25	**6y**	$CH(CH_3)CH_2CH_3$	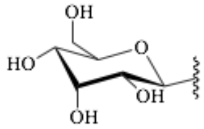	70
26	**6z**	$CH(CH_3)CH_2CH_3$	H	57
27	**6a'**	$CH(CH_3)_2$	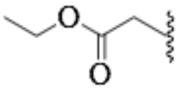	81
28	**6b'**	$CH(CH_3)_2$	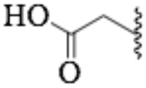	74
29	**6c'**	$CH(CH_3)_2$	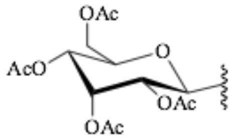	64
30	**6d'**	$CH(CH_3)_2$	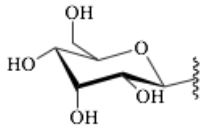	55
31	**6e'**	$CH(CH_3)_2$	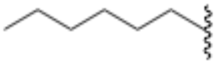	75

Crystallographic data collection details. Intensity data of suitably sized crystal was collected on Oxford Xcalibur S diffractometer (4-circle kappa goniometer, Sapphire−3 CCD detector, omega scans, graphite monochromator, and a single wavelength Enhance X-ray source with MoKα radiation)[S1]. Pre-experiment, data collection, data reduction and absorption corrections were performed with the CrysAlisPro software suite[S2]. The frames were collected by ω, ϕ and 2θ rotation at 10 s per frame with SMART. The measured intensities were reduced to F^2 and corrected for absorption with SADABS[S3]. The structures were solved by direct methods using SIR 92[S4] which revealed the atomic positions, and refined using the SHELX-97 program package[S5] and SHELXL 97[S6] (within the WinGX program package)[S7]. Non-hydrogen atoms were refined anisotropically. C-H hydrogen atoms were placed in geometrically calculated positions by using a riding model. The molecular structures were created with a Diamond program[S8].

Table S2. Crystal data collection and structure refinement parameters for **6a** (CCDC: 1529503).

Empirical formula	$C_{32}H_{26}N_6O_2$
Formula weight	526.59
Temperature	293(2) K
Wavelength	0.71073 Å
Crystal system	Monoclinic
Space group	*C 2/c*
A	30.119(5) Å
B	8.937(5) Å
C	23.764(5) Å
α	90°
β	119.371(11)°
γ	90°
Volume	5574.4(3) $Å^3$
Z	8
Density (calculated)	1.255 Mg/m^3
Absorption coefficient	0.081 mm^{-1}
F(000)	2208
Crystal size	0.24 x 0.22 x 0.18 mm^3
Theta range for data collection	3.065 to 25.00°
Index ranges	$-33 \leq h \leq 35$, $-10 \leq k \leq 10$, $-28 \leq l \leq 28$
Reflections collected	24417
Independent reflections	4914 [*R*(int) = 0.0999]
Completeness to theta = 25.00°	99.8 %
Absorption correction	Multi-scan
Max. and min. transmission	0.9855 and 0.9807
Refinement method	Full-matrix least-squares on F^2
Data / restraints / parameters	4914 / 0 / 363
Goodness-of-fit on F^2	1.128
Final *R* indices [I>2sigma(I)][a, b]	R_1 = 0.0947, wR_2 = 0.2247
R indices (all data)	R_1 = 0.1245, wR_2 = 0.2449
Largest diff. peak and hole	0.390 and -0.367 e.$Å^{-3}$

[a]$R = \sum(\|F_o| - |F_c\|)/\sum|F_o|$; [b]$wR = \{\sum[w(F_o^2 - F_c^2)^2]/\sum[w(F_o^2)^2]\}^{1/2}$

Table S3. Phthalimide Analogues and Standard Antimalarial Combination Interactions.

***P. falciparum* strain**	**Drug Combination**	**Mean FIC (95% confidence interval (CI))**	**Interpretation**
3D7	Chloroquine-**6a**	0.65 (0.23 - 0.88)	Synergy
	Chloroquine -**6h**	0.92 (0.47 - 1.34)	Synergy
	Chloroquine -**6u**	0.69 (0.23 - 0.97)	Synergy
	Dihydroartemisinin-**6a**	0.34 (0.18 - 0.67)	Synergy
	Dihydroartemisinin-**6h**	0.96 (0.54 - 1.2)	Synergy
	Dihydroartemisinin-**6u**	0.86 (0.36 - 1.32)	Synergy
W2	Chloroquine-**6a**	0.51 (0.33 - 1.25)	Synergy
	Chloroquine -**6h**	0.78 (0.23 – 1.1)	Synergy
	Chloroquine -**6u**	0.9 (0.5 -1.3)	Synergy
	Dihydroartemisinin-**6a**	0.41 (0.2 – 0.77)	Synergy
	Dihydroartemisinin-**6h**	0.87 (0.32 – 1.2)	Synergy
	Dihydroartemisinin-**6u**	0.63 (0.5 – 0.9)	Synergy

Note: FIC =1 indicates additivity; FIC<1 synergy and FIC>1 indicates antagonistic effect.

The details of spectroscopic data of compounds 3a, 5a-c and 6a-e' are as follows.

(*R*)-2-(1-(1*H*-benzo[*d*]imidazol-2-yl)-3-methylbutyl)isoindoline-1,3-dione (3a).

It was obtained as a white solid. Mp: 222-224 °C; 76% yield; ^{1}H NMR ($CDCl_3$, 400 MHz): δ 0.97-1.00 (6H, m), 1.48-1.52 (1H, m), 2.06-2.14 (1H, m), 2.39-2.45 (1H, m), 5.91-5.95 (1H, m), 7.23-7.28 (2H, m), 7.58-7.60 (2H, m), 7.73-7.75 (2H, m), 7.84-7.87 (2H, m); ^{13}C NMR ($CDCl_3$, 100 MHz): δ 21.70, 22.67, 25.18, 40.47, 48.34, 122.91, 123.61, 131.41, 134.42, 151.82, 168.36; **HRMS** calculated for $C_{20}H_{20}N_3O_2$: 334.1556; found $[M+H]^+$: 334.1515.

(*R*)-2-(3-methyl-1-(1-(prop-2-yn-1-yl)-1H-benzo[d]imidazol-2-yl)butyl)isoindoline-1,3-dione (5a).

It was obtained as a white solid. mp: 150-152 °C; 76% yield; ^{1}H NMR ($CDCl_3$, 400 MHz): δ 1.02 (3H, d, *J* = 6.87 Hz), 1.06 (3H, d, *J* = 6.10 Hz), 1.62-1.69 (1H, m), 1.74 (1H, t, *J* = 2.29 Hz), 2.32-2.39 (1H, m), 2.89-2.94 (1H, m), 4.88-4.89 (2H, m), 5.73 (1H, dd, *J* = 4.58 Hz, *J* = 10.68 Hz), 7.25-7.30 (2H, m), 7.33-7.36 (1H, m), 7.67-7.71 (2H, m), 7.80-7.83 (2H, m), 7.84-7.87 (1H, m); ^{13}C NMR ($CDCl_3$, 100 MHz): δ 21.62, 23.21, 24.78, 32.89, 38.37, 44.92, 72.95, 109.11, 120.47, 122.50, 123.32, 123.37, 131.64, 124.06, 134.82, 141.96, 150.49, 167.54; HRMS calculated for $C_{23}H_{21}N_3O_2$: 372.1704; found $[M+H]^+$: 372.1698.

2-((1*R*,2*R*)-2-methyl-1-(1-(prop-2-yn-1-yl)-1*H*-benzo[*d*]imidazol-2-yl)butyl)isoindoline-1,3-dione (5b).

It was obtained as a white solid. mp: 118-120 °C; 47% yield; ^{1}H NMR ($CDCl_3$, 400 MHz): δ 0.94-1.27 (7H, m), 1.53-1.71 (1H, m), 1.92-1.93 (1H, m), 3.42-3.48 (1H, m), 4.90-5.12 (2H, m), 5.25-5.29 (1H, m), 7.27-7.30 (2H, m), 7.36-7.38 (1H, m), 7.67-7.69 (2H, m), 7.80-7.82 (2H, m), 7.86-7.88 (1H, m); ^{13}C NMR ($CDCl_3$, 100 MHz): δ 10.49, 10.93, 15.34, 16.94, 25.37, 26.96, 32.83, 33.62, 34.08, 39.51, 51.70, 73.08, 109.39, 120.59, 122.54, 123.28, 123.44, 131.49, 134.08, 134.31, 134.41, 142.27, 149.74, 149.92, 167.63; HRMS calculated for $C_{23}H_{21}N_3O_2$: 372.1704; found $[M+H]^+$: 372.1690.

(*R*)-2-(2-methyl-1-(1-(prop-2-yn-1-yl)-1*H*-benzo[*d*]imidazol-2-yl)propyl)isoindoline-1,3-dione (5c).

It was obtained as a white solid. Mp: 170-172 °C; 72% yield; ^{1}H NMR ($CDCl_3$, 400 MHz): δ 1.04 (3H, d, *J* = 6.41 Hz), 1.16 (3H, d, *J* = 6.41 Hz), 1.90 (1H, t, *J* = 2.29 Hz, *J* = 2.75 Hz), 3.63-3.69 (1H, m), 4.91-5.08 (2H, m), 5.19 (1H, d, *J* = 10.99 Hz), 7.28-7.34 (2H, m), 7.38-7.40 (1H, m), 7.68-7.72 (2H, m), 7.80-7.83 (2H, m), 7.92-7.94 (1H, m); ^{13}C NMR ($CDCl_3$, 100 MHz): δ 19.49, 21.12, 28.24, 33.13, 52.92, 73.41, 109.63, 120.47, 123.64, 123.75, 131.55, 134.32, 167.66; HRMS calculated for $C_{22}H_{19}N_3O_2$: 358.1547; found $[M+H]^+$: 358.1542.

(*R*)-2-(3-methyl-1-(1-((1-phenyl-1*H*-1,2,3-triazol-4-yl)methyl)-1*H*-benzo[*d*]imidazol-2-yl)butyl)isoindoline-1,3-dione (6a).

It was obtained as light brown solid. mp: 209-211°C; 76% yield; ^{1}H NMR (400 MHz, $CDCl_3$): δ 0.98 (3H, d, *J* = 6.71 Hz), 1.05 (3H, d, *J* = 6.71 Hz), 1.56-1.63 (1H, m), 2.32-2.40 (1H, m), 2.80-2.87 (1H, m), 5.51 (2H, s), 5.81 (1H, dd, *J* = 4.27 Hz, *J* = 11.00 Hz), 7.27-7.32 (5H, m), 7.34-7.40 (4H, m), 7.47-7.50 (2H, m), 7.54-7.58 (2H, m), 7.91 (1H, d, *J* = 9.77 Hz); ^{13}C NMR (100 MHz, $CDCl_3$,): δ 21.62, 23.28, 24.78, 38.74, 39.97, 45.10, 109.34, 119.09, 119.98, 120.37, 123.02, 123.79, 128.79, 129.51, 130.87, 134.69, 136.69, 136.21, 150.60, 167.38; HRMS calculated for $C_{29}H_{26}N_6O_2$: 491.2191; found $[M+H]^+$: 491.2199.

(*R*)-2-(1-(1-((1-(4-chlorophenyl)-1*H*-1,2,3-triazol-4-yl)methyl)-1*H*-benzo[*d*]imidazol-2-yl)-3-methylbutyl)isoindoline-1,3-dione (6b).

It was obtained as off white solid. mp: 213-215 °C; 62% yield; ^{1}H NMR (400 MHz, $CDCl_3$): δ 0.99 (3H, d, *J* = 6.87 Hz), 1.05 (3H, d, *J* = 6.87 Hz), 1.56-1.62 (1H, m), 2.31-2.39 (1H, m), 2.79-2.87 (1H, m), 5.50 (2H, s), 5.81 (1H, dd, *J* = 4.58 Hz), 7.17-7.32 (6H, m), 7.35-7.37 (2H, m), 7.50-7.51 (2H, m), 7.56-7.57 (2H, m), 7.91 (1H, d, *J* = 7.63 Hz); ^{13}C NMR (100 MHz, $CDCl_3$): δ 21.61, 23.32, 24.76, 38.77, 39.71, 45.25, 109.03, 118.85, 120.72, 121.07, 122.67, 123.16, 123.56, 129.67, 130.88, 133.98, 134.53, 134.71, 135.31, 142.28, 144.25, 151.02, 167.40; HRMS calculated for $C_{29}H_{25}ClN_6O_2$: 525.1802; found $[M+H]^+$: 525.1802.

(*R*)-2-(1-(1-((1-(4-fluorophenyl)-1*H*-1,2,3-triazol-4-yl)methyl)-1*H*-benzo[*d*]imidazol-2-yl)-3-methylbutyl)isoindoline-1,3-dione (6c).

It was obtained an off white solid. mp: 193-195 °C; 54% yield; ^{1}H NMR (400 MHz, $CDCl_3$): δ 0.98 (3H, d, *J* = 6.10 Hz), 1.05 (3H, d, *J* = 6.10 Hz), 1.56-1.62 (1H, m), 2.31-2.38 (1H, m,), 2.80-2.87 (1H, m), 5.50 (2H, s), 5.82 (1H, dd, *J* = 4.58 Hz, *J* = 10.68 Hz), 7.06-7.14 (3H, m), 7.23-7.31 (5H, m), 7.49-7.53 (2H, m), 7.56-7.59 (2H, m), 7.90 (1H, d, *J* = 9.92 Hz); ^{13}C NMR (100 MHz, $CDCl_3$): δ 21.59, 23.32, 24.75, 38.74, 39.68, 45.21, 109.04, 116.57, 119.07, 120.67, 121.91, 122.64, 123.15, 123.53, 130.87, 132.47, 133.97, 135.29, 142.24, 144.13, 151.00, 167.40; HRMS calculated for $C_{29}H_{25}FN_6O_2$:509.2097; found $[M+H]^+$: 509.2081.

(*R*)-2-(1-(1-((1-(4-methoxyphenyl)-1*H*-1,2,3-triazol-4-yl)methyl)-1*H*-benzo[*d*]imidazol-2-yl)-3-methylbutyl)isoindoline-1,3-dione (6d).

It was obtained as pink solid. mp: 189-191 °C; 71% yield; ^{1}H NMR (400 MHz, $CDCl_3$): δ 1.02 (3H, d, *J* = 6.59 Hz), 1.08 (3H, d, *J* = 6.59 Hz), 1.57-1.66 (1H, m), 2.43-2.50 (1H, m), 2.86-2.94 (1H, m), 3.83 (3H, s), 5.58 (2H, s), 5.93 (1H, dd, *J* = 4.39 Hz, *J* = 10.98 Hz), 6.90 (2H, d, *J* = 9.52 Hz), 7.26-7.28 (2H, m), 7.33-7.47 (4H, m), 7.56-7.58 (2H, m), 7.63-7.65 (2H, m), 8.09 (1H, d, *J* = 6.59 Hz); ^{13}C NMR (100 MHz, $CDCl_3$): δ 21.55, 23.25, 24.72, 38.64, 39.84, 44.96, 55.54, 109.37, 114.44, 119.12, 120.22, 121.52, 122.91, 123.22, 123.81, 129.62, 130.83, 134.03, 135.08, 143.07, 150.82, 159.70, 167.35; HRMS calculated for $C_{30}H_{28}N_6O_3$: 521.2297; found $[M+H]^+$: 521.2283.

(*R*)-2-(3-methyl-1-(1-((1-(*p*-tolyl)-1*H*-1,2,3-triazol-4-yl)methyl)-1*H*-benzo[*d*]imidazol-2-yl)butyl)isoindoline-1,3-dione (6e).

It was obtained as off white solid. mp: 212-214 °C; 85% yield; ^{1}H NMR (400 MHz, $CDCl_3$): δ 1.11 (3H, d, *J* = 6.71 Hz), 1.17 (3H, d, *J* = 6.10 Hz), 1.66-1.75 (1H, m), 2.43-2.52 (4H, m,), 2.92-2.99 (1H, m), 5.62 (2H, s), 5.95 (1H, dd, *J* = 4.27 Hz, *J* = 10.38 Hz), 7.27-7.30 (4H, m), 7.38-7.43 (4H,

m), 7.61-7.64 (2H, m), 7.69-7.70 (2H, m), 8.02-8.04 (1H, m); ^{13}C NMR (100 MHz, $CDCl_3$): δ 21.01, 21.62, 23.32, 24.76, 38.77, 39.79, 45.21, 109.15, 118.90, 119.86, 120.62, 122.62, 123.18, 123.52, 129.95, 130.88, 133.96, 135.35, 138.87, 142.21, 143.75, 151.06, 167.44; HRMS calculated for $C_{30}H_{28}N_6O_2$: 505.2348; found $[M+H]^+$: 505.2347.

2-((1*R*,2*R*)-2-methyl-1-(1-((1-phenyl-1*H*-1,2,3-triazol-4-yl)methyl)-1*H*-benzo[*d*]imidazol-2-yl)butyl)isoindoline-1,3-dione (6f).

It was obtained as white solid. mp. 211-213 °C; 51% yield; ^{1}H NMR (400 MHz, $CDCl_3$): δ 0.90-1.09 (6H, m), 1.11-1.26 (1H, m), 1.44-1.75 (1H, m), 3.35-3.40 (1H, m), 5.37-5.42 (1H, m), 5.49-5.67 (2H, m), 7.21-7.28 (3H, m), 7.31-7.38 (6H, m), 7.49-7.51 (2H, m), 7.61-7.63 (2H, m), 7.89 (1H, d, *J* = 8.39 Hz); ^{13}C NMR (100MHz, $CDCl_3$): δ 10.63, 11.02, 15.27, 17.10, 25.30, 27.09, 33.92, 34.39, 39.47, 51.32, 51.82, 109.47, 119.20, 120.05, 120.70, 122.58, 123.29, 123.41, 128.75, 129.49, 130.94, 133.99, 142.43, 143.98, 144.03, 150.07, 150.19, 167.44, 167.50; HRMS calculated for $C_{29}H_{26}N_6O_2$: 491.2191; found $[M+H]^+$: 491.2198.

2-((1*R*,2*R*)-1-(1-((1-(4-chlorophenyl)-1*H*-1,2,3-triazol-4-yl)methyl)-1*H*-benzo[*d*]imidazol-2-yl)-2-methylbutyl)isoindoline-1,3-dione (6g).

It was obtained as white solid. mp: 194-196 °C; 72% yield; ^{1}H NMR (400 MHz, $CDCl_3$): δ 0.91-1.10 (6H, m), 1.12-1.23 (1H, m), 1.45-1.72 (1H, m), 3.36-3.41 (1H, m), 5.37-5.41 (1H, m), 5.49-5.67 (2H, m), 7.22-7.37 (8H, m), 7.52-7.54 (2H, m), 7.62-7.65 (2H, m), 7.90 (1H, d, *J* = 7.63 Hz); ^{13}C NMR (100 MHz, $CDCl_3$): δ 10.62, 11.02, 15.28, 17.10, 25.31, 27.09, 33.95, 34.42, 39.41, 51.36, 51.85, 109.41, 119.31, 120.75, 121.19, 122.58, 123.27, 123.43, 129.67, 130.96, 134.02, 134.54, 134.77, 142.46, 144.26, 150.05, 167.41; HRMS calculated for $C_{29}H_{25}ClN_6O_2$: 525.1802; found $[M+H]^+$: 525.1820.

2-((1*R*,2*R*)-1-(1-((1-(4-fluorophenyl)-1*H*-1,2,3-triazol-4-yl)methyl)-1*H*-benzo[*d*]imidazol-2-yl)-2-methylbutyl)isoindoline-1,3-dione (6h).

It was obtained as white solid. mp:182-184 °C; 61% yield; ^{1}H NMR (400 MHz, $CDCl_3$): δ 0.92-1.10 (6H, m), 1.13-1.26 (1H, m), 1.45-1.74 (1H, m), 3.37-3.44 (1H, m), 5.38-5.43 (1H, m), 5.50-5.69 (2H, m), 7.06-7.11 (2H, m), 7.23-7.29 (3H, m), 7.30-7.36 (3H, m), 7.54-7.56 (2H, m), 7.65-7.67 (2H, m), 7.91 (1H, d, *J* = 7.63 Hz); ^{13}C NMR (100 MHz, $CDCl_3$): δ 10.62, 11.03, 15.28, 17.09, 25.32, 27.09, 33.94, 34.41, 39.41, 51.36, 51.85, 109.39, 109.43, 116.37, 116.61, 120.74, 122.00, 122.08, 123.29, 130.99, 134.01, 134.72, 142.47, 144.16, 144.21, 150.18, 167.43, 167.49; HRMS calculated for $C_{29}H_{25}FN_6O_2$: 509.2097; found $[M+H]^+$: 509.2093.

2-((1*R*,2*R*)-1-(1-((1-(4-methoxyphenyl)-1*H*-1,2,3-triazol-4-yl)methyl)-1*H*-benzo[*d*]imidazol-2-yl)-2-methylbutyl)isoindoline-1,3-dione (6i).

It was obtained as white solid. mp: 135-137 °C; 77% yield; ^{1}H NMR (400 MHz, $CDCl_3$): δ 0.92-1.10 (6H, m), 1.15-1.26 (1H, m), 1.47-1.74 (1H, m), 3.38-3.43 (1H, m), 3.81 (3H, s), 5.38-5.43 (1H, m), 5.49-5.68 (2H, m), 6.86-6.88 (2H, m), 7.20-7.28 (5H, m), 7.34-7.36 (1H, m), 7.54-7.56 (2H, m), 7.64-7.67 (2H, m), 7.90-7.92 (1H, m); ^{13}C NMR (100 MHz, $CDCl_3$): δ 10.62, 11.02, 15.27, 17.08, 25.30, 27.07, 33.91, 34.39, 39.47, 51.32, 51.80, 55.55, 109.51, 114.46, 119.25, 119.32, 120.64,

121.68, 122.55, 123.30, 123.38, 130.96, 134.00, 134.75, 142.41, 143.79, 150.20, 159.72, 167.45, 167.52; HRMS calculated for $C_{30}H_{28}N_6O_3$: 521.2297; found $[M+H]^+$: 521.2319.

2-((1*R*,2*R*)-2-methyl-1-(1-((1-(*p*-tolyl)-1*H*-1,2,3-triazol-4-yl)methyl)-1*H*-benzo[*d*]imidazol-2-yl)butyl)isoindoline-1,3-dione (6j).

It was obtained as white solid. mp: 198-201 °C; 68% yield; ^{1}H NMR (400 MHz, $CDCl_3$): δ 0.89-1.08 (6H, m), 1.13-1.21 (1H, m), 1.44-1.71 (1H, m), 2.33 (3H, s), 3.35-3.43 (1H, m), 5.37-5.41 (1H, m), 5.46-5.66 (2H, m), 7.13-7.27 (7H, m), 7.32-7.34 (1H, m), 7.50-7.53 (2H, m), 7.61-7.63 (2H, m), 7.87-7.89 (1H, m); ^{13}C NMR (100 MHz, $CDCl_3$): δ 10.63, 11.03, 15.28, 17.10, 21.02, 25.31, 27.09, 33.93, 34.40, 39.48, 51.32, 51.82, 109.51, 119.99, 120.68, 123.30, 123.37, 129.97, 130.96, 134.00, 134.77, 138.90, 142.45, 143.79, 143.86, 150.21, 167.45, 167.52; HRMS calculated for $C_{30}H_{28}N_6O_2$: 505.2348; found $[M+H]^+$: 505.2333.

(*R*)-2-(2-methyl-1-(1-((1-phenyl-1*H*-1,2,3-triazol-4-yl)methyl)-1*H*-benzo[*d*]imidazol-2-yl)propyl)isoindoline-1,3-dione (6k).

It was obtained as white solid. mp: 221-223; 54% yield; ^{1}H NMR (400 MHz, $CDCl_3$): δ 1.01 (3H, d, *J* = 6.87 Hz), 1.18 (3H, d, *J* = 6.10 Hz), 3.53-3.62 (1H, m), 5.32 (1H, d, *J* = 10.68 Hz), 5.56 (2H, dd, *J* = 16.78 Hz, *J* = 39.72 Hz), 7.23-7.28 (9H, m), 7.31-7.41 (6H, m) 7.50-7.54 (2H, m), 7.61-7.64 (2H, m), 7.91-7.93 (1H, m); ^{13}C NMR (100 MHz, $CDCl_3$): δ 19.26, 21.14, 28.41, 39.51, 52.89, 109.38, 119.04, 120.02, 120.70, 122.59, 123.27, 123.46, 128.76, 129.48, 130.87, 134.00, 134.83, 136.24, 142.41, 144.03, 149.95, 167.42; HRMS calculated for $C_{28}H_{24}N_6O_2$: 477.2035; found $[M+H]^+$: 477.2056.

(*R*)-2-(1-(1-((1-(4-chlorophenyl)-1*H*-1,2,3-triazol-4-yl)methyl)-1*H*-benzo[*d*]imidazol-2-yl)-2-methylpropyl)isoindoline-1,3-dione (6l).

It was obtained as off white solid. mp: 182-184 °C; 68% yield; ^{1}H NMR (400 MHz, $CDCl_3$): δ 1.08 (3H, d, *J* = 6.10 Hz), 1.18 (3H, d, *J* = 6.87 Hz), 3.51-3.61 (1H, m), 5.29-5.32 (1H, m), 5.59 (2H, dd, *J* = 16.78 Hz, *J* = 17.55 Hz, *J* = 36.62 Hz), 7.21 (1H, s), 7.27-7.32 (5H, m), 7.34-7.37 (2H, m), 7.52-7.54 (2H, m), 7.61-7.64 (2H, m), 7.90-7.92 (1H, m); ^{1}H NMR (100 MHz, $CDCl_3$): δ 19.25, 21.13, 28.43, 39.43, 52.89, 109.32, 118.97, 120.73, 121.14, 122.63, 123.26, 123.49, 129.66, 130.86, 134.03, 134.53, 134.71, 134.78, 142.41, 144.30, 149.92, 167.38; HRMS calculated for $C_{28}H_{23}ClN_6O_2$: 511.1645; found $[M+H]^+$: 511.1647.

(*R*)-2-(1-(1-((1-(4-fluorophenyl)-1*H*-1,2,3-triazol-4-yl)methyl)-1*H*-benzo[*d*]imidazol-2-yl)-2-methylpropyl)isoindoline-1,3-dione (6m).

It was obtained as off white solid. mp: 173-175 °C; 77% yield; ^{1}H NMR (400 MHz, $CDCl_3$): δ 0.94 (3H, d, *J* = 6.87 Hz), 1.11 (3H, d, *J* = 6.10 Hz), 3.46-3.54 (1H, m), 5.25 (1H, d, *J* = 10.68 Hz), 5.53 (2H, dd, *J* = 16.78 Hz, *J* = 17.55 Hz, *J* = 38.92 Hz), 6.99-7.03 (2H, m), 7.14-7.28 (6H, m), 7.46-7.49 (2H, m), 7.55-7.58 (2H, m), 7.83-7.85 (1H, m); ^{13}C NMR (100 MHz, $CDCl_3$): 19.26, 21.12, 28.42, 39.44, 52.90, 109.35, 116.35, 116.59, 119.22, 120.72, 121.94, 122.03, 122.62, 123.27, 123.47, 130.90, 134.01, 134.78, 142.41, 144.19, 149.93, 167.40; HRMS calculated for $C_{28}H_{23}FN_6O_2$: 495.1941; found $[M+H]^+$: 495.1948.

(*R*)-2-(1-(1-((1-(4-methoxyphenyl)-1*H*-1,2,3-triazol-4-yl)methyl)-1*H*-benzo[*d*]imidazol-2-yl)-2-methylpropyl)isoindoline-1,3-dione (6n).

It was obtained as pink solid. mp: 125-127 ^oC; 42% yield; ^1H NMR (400 MHz, $CDCl_3$): δ 1.10 (3H, d, *J* = 6.71 Hz), 1.27 (3H, d, *J* = 6.10 Hz), 3.62-3.71 (1H, m), 3.91 (3H, s), 5.42 (1H, d, *J* = 10.99 Hz), 5.68 (2H, dd, *J* = 16.48 Hz, *J* = 17.09 Hz, *J* = 40.89 Hz), 6.95-6.98 (3H, m), 7.31-7.45 (5H, m), 7.63-7.65 (2H, m), 7.73-7.75 (2H, m), 8.01 (1H, d, *J* = 9.61 Hz); ^{13}C NMR (100 MHz, $CDCl_3$): δ 19.25, 21.12, 28.41, 39.51, 52.89, 55.55, 109.42, 115.07, 118.98, 120.30, 121.82, 122.38, 123.32, 129.73, 130.88, 134.00, 134.83, 142.39, 143.78, 149.96, 159.73, 167.43; HRMS calculated for $C_{29}H_{26}N_6O_3$: 507.2140; found $[M+H]^+$: 507.2122.

(*R*)-2-(2-methyl-1-(1-((1-(*p*-tolyl)-1*H*-1,2,3-triazol-4-yl)methyl)-1*H*-benzo[*d*]imidazol-2-yl)propyl)isoindoline-1,3-dione (6o).

It was obtained as light brown solid. mp: 169-171 ^oC; 37% yield; ^1H NMR (400 MHz, $CDCl_3$): δ 1.11 (3H, d, *J* = 6.71 Hz), 1.28 (3H, d, *J* = 6.71 Hz), 2.46 (3H, s), 3.62-3.72 (1H, m), 5.42 (1H, d, *J* = 10.38 Hz), 5.60-5.75 (2H, m), 7.26-7.31 (4H, m), 7.36-7.45 (4H, m), 7.62-7.64 (2H, m), 7.73-7.75 (2H, m), 8.02 (1H, d, *J* = 7.32 Hz); ^{13}C NMR (100 MHz, $CDCl_3$): δ 19.26, 21.25, 28.43, 39.54, 52.91, 109.42, 119.04, 119.26, 120.69, 122.57, 123.29, 123.43, 129.96, 130.90, 134.01, 138.90, 143.85, 149.98, 167.44; HRMS calculated for $C_{29}H_{26}N_6O_2$: 491.2191; found $[M+H]^+$: 491.2192.

Ethyl-(*R*)-2-(4-((2-(1-(1,3-dioxoisoindolin-2-yl)-3-methylbutyl)-1*H*-benzo[*d*]imidazol-1-yl)methyl)-1*H*-1,2,3-triazol-1-yl)acetate (6p).

It was obtained as off white solid. mp: 147-149 ^oC; 74% yield; ^1H NMR (400 MHz, $CDCl_3$): δ 0.98 (3H, d, *J* = 6.87 Hz), 1.03 (3H, d, *J* = 6.10 Hz), 1.21 (3H, t, *J* = 6.10 Hz, *J* = 8.39 Hz), 1.57-1.64 (1H, m), 2.25-2.32 (1H, m), 2.83-2.90 (1H, m), 4.11-4.17 (2H, m), 4.61-4.81 (2H, m), 5.48 (2H, s), 5.82 (1H, dd, *J* = 4.58 Hz, *J* = 11.44 Hz), 7.13(1H, s), 7.21-7.31 (3H, m), 7.67-7.69 (2H, m), 7.73-7.75 (2H, m), 7.85-7.87 (1H, m); ^{13}C NMR (100 MHz, $CDCl_3$): δ 13.93, 21.59, 23.22, 24.83, 38.75, 39.48, 45.21, 50.52, 62.37, 109.34, 120.50, 122.49, 122.68, 123.26, 131.33, 134.01, 135.13, 142.18, 143.40, 150.97, 165.55, 167.49; HRMS calculated for $C_{27}H_{28}N_6O_4$: 501.2246; found $[M+H]^+$: 501.2265.

(*R*)-2-(4-((2-(1-(1,3-dioxoisoindolin-2-yl)-3-methylbutyl)-1*H*-benzo[*d*]imidazol-1-yl)methyl)-1*H*-1,2,3-triazol-1-yl)acetic acid (6q).

It was obtained as off white solid. mp: > 300 ^oC; 65% yield; ^1H NMR (400 MHz, DMSO): δ 0.91 (6H, s), 1.73-1.78 (1H, m), 1.85-1.90 (1H, m), 2.00-2.06 (1H, m), 4.65 (2H, s), 5.49-5.54 (1H, m), 5.69-5.71 (1H, m), 5.81-5.85 (1H, m), 7.14-7.18 (2H, m), 7.34-7.38 (2H, m), 7.50-7.51 (1H, m), 7.58-7.62 (3H, m), 7.98 (1H, s), 10.14 (1H, s); ^{13}C NMR (100 MHz, $CDCl_3$): δ 21.75, 22.09, 22.94, 24.44, 42.38, 43.91, 44.73, 53.63, 110.92, 118.96, 119.33, 121.55, 122.07, 123.07, 123.73, 124.53, 127.46, 128.55, 129.17, 129.54, 131.20, 134.51, 134.84, 135.45, 141.03, 141.70, 142.02, 142.16, 151.14, 154.98, 167.51, 172.69; HRMS calculated for $C_{25}H_{24}N_6O_4$: 473.1929; found $[M+H]^+$: 473.1952.

(2*S*,4*R*,5*S*)-2-(acetoxymethyl)-6-(4-((2-((*R*)-1-(1,3-dioxoisoindolin-2-yl)-3-methylbutyl)-1*H*-benzo[*d*]imidazol-1-yl)methyl)-1*H*-1,2,3-triazol-1-yl)tetrahydro-2*H*-pyran-3,4,5-triyl triacetate (6r).

It was obtained as off white solid. mp: 115-117 °C; 76% yield; ^{1}H NMR (400 MHz, $CDCl_3$): δ 0.97-1.00 (3H, m), 1.02-1.05 (3H, m), 1.57-1.66 (1H, m) 1.74-1.76 (3H, m), 1.99-2.10 (9H, m), 2.22-2.33 (1H, m), 2.83-2.98 (1H, m), 3.87-3.91 (1H, m), 4.06-4.25 (2H, m), 5.12-5.17 (2H, m), 5.24-5.34 (1H, m), 5.43-5.49 (2H, m), 5.55-5.66 (1H, m), 5.80-5.89 (1H, m), 7.23-7.26 (1H, m), 7.27-7.30 (2H, m), 7.42-7.55 (1H, m), 7.67-7.70 (1H, m), 7.72-7.78 (2H, m), 7.81-7.89 (1H, m); ^{13}C NMR (100 MHz, $CDCl_3$): δ 19.97, 20.04, 20.44, 20.46, 20.65, 21.56, 21.61, 23.14, 23.18, 24.80, 24.93, 38.74, 38.89, 39.18, 45.18, 61.43, 61.56, 67.48, 67.59, 69.81, 70.14, 72.19, 72.47, 74.92, 75.07, 85.36, 85.57, 109.51, 109.58, 120.06, 120.43, 120.59, 122.46, 122.50, 123.29, 123.41, 123.47, 131.35, 131.64, 133.98, 134.11, 134.81, 135.03, 142.16, 143.03, 143.52, 150.95, 167.56, 167.84, 168.60, 168.74, 169.23, 169.32, 169.80, 170.42, 170.46; HRMS calculated for $C_{37}H_{40}N_6O_{11}$: 745.2829; found $[M+H]^+$: 745.2844

2-((1*R*)-3-methyl-1-(1-((1-((3*S*,4*R*,6*S*)-3,4,5-trihydroxy-6-(hydroxymethyl)tetrahydro-2*H*-pyran-2-yl)-1*H*-1,2,3-triazol-4-yl)methyl)-1*H*-benzo[*d*]imidazol-2-yl)butyl)isoindoline-1,3-dione (6s).

It was obtained as off white solid. mp: 123-125 °C; 62% yield; ^{1}H NMR (400 MHz, D_2O): δ 0.69-0.73 (6H, m), 1.47-1.55 (1H, m), 1.67-1.72 (1H, m), 1.85-1.94 (1H, m), 3.39-3.44 (1H, m), 3.49-3.59 (3H, m), 3.65-3.76 (2H, m), 5.32-5.38 (1H, m), 5.49-5.52 (1H, m), 5.55-5.73 (1H, m), 7.03-7.09 (1H, m), 7.19-7.26 (3H, m), 7.35-7.43 (3H, m), 7.54-7.59 (1H, m), 7.96-8.01 (1H, m); 13C NMR (100 MHz, D_2O): δ 21.31, 21.93, 23.24, 24.35, 41.60, 44.77, 60.27, 68.84, 72.18, 75.89, 78.72, 87.47, 110.79, 118.34, 123.01, 123.09, 123.20, 123.76, 127.17, 127.09, 129.24, 130.56, 133.33, 134.52, 140.60, 143.60, 154.45, 171.81, 175.62, 181.41; HRMS calculated for $C_{29}H_{32}N_6O_7$: 577.2406; found $[M+H]^+$: 577.2406.

(*R*)-2-(1-(1-((1-hexyl-1*H*-1,2,3-triazol-4-yl)methyl)-1*H*-benzo[*d*]imidazol-2-yl)-3-methylbutyl)isoindoline-1,3-dione (6t).

It was obtained as white solid. mp: 146-148 °C; 32% yield; ^{1}H NMR (400 MHz, $CDCl_3$): δ 0.82 (3H, t, J = 6.87 Hz), 0.96 (3H, d, J = 6.87 Hz), 1.02 (3H, d, J = 6.87 Hz), 1.12-1.23 (6H, m), 1.52-1.60 (3H, m), 2.24-2.31 (1H, m), 2.80-2.88 (1H, m), 3.82-3.94 (2H, m), 5.43 (2H, s), 5.78 (1H, dd, J = 4.58 Hz, J = 10.68 Hz), 6.85 (1H, s), 7.20-7.27 (3H, m), 7.65-7.68 (2H, m), 7.70-7.73 (2H, m), 7.85-7.87 (1H, m); ^{13}C NMR (100MHz, $CDCl_3$): δ 13.82, 21.58, 22.26, 23.20, 24.78, 25.90, 29.87, 30.92, 38.71, 39.65, 45.16, 50.11, 109.30, 120.46, 120.78, 123.22, 123.31, 131.22, 134.03, 135.18, 142.16, 142.94, 150.94, 167.41; HRMS calculated for $C_{29}H_{34}N_6O_2$: 499.2817; found $[M+H]^+$: 499.2820.

(*R*)-2-(1-(1-((1*H*-1,2,3-triazol-4-yl)methyl)-1*H*-benzo[*d*]imidazol-2-yl)-3-methylbutyl)isoindoline-1,3-dione (6u).

It was obtained as white solid. mp: 193-195 °C; 64% yield; ^{1}H NMR (400 MHz, $CDCl_3$): δ 0.86 (3H, d, J = 6.87 Hz), 0.95 (3H, d, J = 6.10 Hz), 1.50-1.57 (1H, m), 2.20-2.27 (1H, m), 2.79-2.86 (1H, m), 5.50 (2H, s), 5.83 (1H, dd, J = 4.58 Hz, J = 10.68 Hz), 7.25-7.33 (4H, m), 7.42-7.44 (2H, m), 7.61-7.63 (2H, m), 7.84-7.87 (1H, m); ^{13}C NMR (100 MHz, $CDCl_3$): δ 14.10, 21.52, 22.63, 23.07, 24.76, 31.55, 38.66, 39.24, 44.91, 109.45, 120.40, 122.68, 123.29, 123.50, 131.14, 134.03, 135.11, 141.77, 150.99, 167.57; HRMS calculated for $C_{23}H_{22}N_6O_2$: 415.1877; found $[M+H]^+$: 415.1890.

Ethyl-2-(4-((2-((1*R*,2*R*)-1-(1,3-dioxoisoindolin-2-yl)-2-methylbutyl)-1*H*-benzo[*d*]imidazol-1-yl)methyl)-1*H*-1,2,3-triazol-1-yl)acetate (6v).

It was obtained as off white solid. mp:. 180-182 °C; 72% yield; ^{1}H NMR (400 MHz, $CDCl_3$): δ 0.92-1.24 (10H, m), 1.50-157 (1H, m), 3.37-3.48 (1H, m), 4.18 (2H, q, *J* = 6.87 Hz, *J* = 14.50 Hz), 4.75-4.91 (2H, m) 5.39 (1H, d, *J* = 10.68 Hz), 5.46-5.73 (2H, m), 7.22-7.26 (3H, m), 7.34-7.36 (1H, m), 7.67-7.69 (2H, m), 7.76-7.79 (2H, m), 7.86-7.88 (1H, m); ^{13}C NMR (100 MHz, $CDCl_3$): δ 10.54, 11.04, 13.94, 15.33, 16.83, 25.38, 26.93, 33.84, 34.34, 39.22, 50.60, 51.58, 51.83, 62.39, 109.69, 120.57, 122.92, 123.29, 123.39, 131.32, 134.05, 142.42, 143.61, 150.32, 165.65, 167.57; HRMS calculated for $C_{27}H_{28}N_6O_4$: 501.2246; found $[M+H]^+$: 501.2260.

2-(4-((2-((1*R*,2*R*)-1-(1,3-dioxoisoindolin-2-yl)-2-methylbutyl)-1*H*-benzo[*d*]imidazol-1-yl)methyl)-1*H*-1,2,3-triazol-1-yl)acetic acid (6w).

It was obtained as off white solid. mp: > 300 °C; 54% yield; ^{1}H NMR (400 MHz, D_2O): δ 0.60-0.98 (6H, m), 1.00-1.11 (1H, m), 1.23-1.62 (1H, m), 2.05-2.19 (1H, m), 4.89 (2H, s), 5.29-5.36 (1H, m), 5.83-6.02 (2H, m), 7.24-7.29 (1H, m), 7.47-7.48 (2H, m), 7.50-7.52 (2H, m), 7.69-7.77 (3H, m), 7.96-7.98 (1H, m); ^{13}C NMR (100 MHz, DMSO): δ 10.68, 11.27, 15.29, 15.89, 24.70, 25.99, 36.55, 37.43, 37.96, 49.38, 51.12, 64.95, 110.83, 118.92, 121.61, 122.05, 122.09, 124.72, 127.75, 127.80, 129.13, 129.21, 130.76, 131.13, 134.64, 134.77, 138.02, 142.13, 142.61, 154.10, 154.33, 167.77, 168.53; HRMS calculated for $C_{25}H_{24}N_6O_4$: 473.1933; found $[M+H]^+$: 473.1934

(2*S*,4*R*,5*S*)-2-(acetoxymethyl)-6-(4-((2-((1*R*,2*R*)-1-(1,3-dioxoisoindolin-2-yl)-2-methylbutyl)-1*H*-benzo[*d*]imidazol-1-yl)methyl)-1*H*-1,2,3-triazol-1-yl)tetrahydro-2*H*-pyran-3,4,5-triyltriacetate (6x).

It was obtained as off white solid. mp: 129-13 °C; 85% yield; ^{1}H NMR (400 MHz, $CDCl_3$): δ 0.86-0.98 (6H, m), 1.01-120 (1H, m), 1.44-1.52 (1H, m), 1.72-1.74 (3H, m), 1.96-2.08 (9H, m) 3.37-3.48 (1H, m), 4.18 (2H, q, *J* = 6.87 Hz, *J* = 14.50 Hz), 4.75-4.91 (2H, m) 5.39 (1H, d, *J* = 10.68 Hz), 5.46-5.73 (2H, m), 7.22-7.26 (3H, m), 7.34-7.36 (1H, m), 7.67-7.69 (2H, m), 7.76-7.79 (2H, m), 7.86-7.88 (1H, m); ^{13}C NMR (100 MHz, $CDCl_3$): δ 10.39, 11.03, 15.35, 16.71, 19.96, 20.05, 20.44, 20.47, 20.63, 25.36, 26.77, 33.76, 34.27, 38.77, 38.93, 51.65, 51.74, 61.47, 67.49, 67.52, 69.90, 70.03, 72.32, 72.48, 74.97, 75.02, 85.49, 85.56, 109.77, 109.84, 120.28, 120.51, 120.57, 122.47, 122.49, 123.26, 123.27, 123.49, 123.50, 131.36, 131.51, 134.07, 134.14, 134.43, 142.37, 143.39, 143.17, 150.19, 150.34, 167.67, 167.85, 168.66, 168.81, 169.28, 169.34, 169.82, 170.42; HRMS calculated for $C_{37}H_{40}N_6O_{11}$: 745.2829; found $[M+H]^+$: 745.2836.

2-((1*R*,2*R*)-2-methyl-1-(1-((1-((3*S*,4*R*,6*S*)-3,4,5-trihydroxy-6-(hydroxymethyl)tetrahydro-2*H*-pyran-2-yl)-1*H*-1,2,3-triazol-4-yl)methyl)-1*H*-benzo[*d*]imidazol-2-yl)butyl)isoindoline-1,3-dione (6y).

It was obtained as white solid. mp: >300 °C; 63% yield; ^{1}H NMR (400 MHz, DMSO): δ 0.65-0.90 (4H, m), 0.99-1.08 (2H, m), 1.32-1.39 (1H, m), 1.71-1.80 (1H, m), 2.18-2.27 (1H, m), 3.16-3.21 (1H, m), 3.29-3.41 (3H, m), 3.58-3.63 (1H, m), 3.67-3.74 (1H, m), 3.77-3.86 (4H, m), 5.36-5.48 (2H, m), 5.55-5.81 (2H, m), 7.15-7.22 (2H, m), 7.26-7.37 (2H, m), 7.40-7.44 (1H, m), 7.53-7.57 (2H, m), 7.60-7.70 (1H, m), 8.35-8.44 (1H, m); ^{13}C NMR (100 MHz, DMSO): δ 10.69, 11.58, 15.22, 15.38,

24.79, 25.91, 37.98, 38.20, 48.85, 50.09, 60.63, 69.67, 69.88, 70.59, 71.93, 76.91, 77.03, 79.80, 80.08, 87.43, 87.56, 110.76, 111.10, 118.68, 121.64, 121.75, 121.93, 122.01, 122.61, 122.79, 126.73, 126.97, 128.11, 128.44, 128.76, 129.00, 129.28, 129.64, 131.65, 134.07, 134.49, 141.74, 142.11, 142.21, 142.36, 142.71, 154.53, 154.82, 167.77, 168.56, 172.39, 172.65; HRMS calculated for $C_{29}H_{32}N_6O_7$: 577.2402; found $[M+H]^+$: 577.2390.

2-((1*R*,2*R*)-1-(1-((1*H*-1,2,3-triazol-4-yl)methyl)-1*H*-benzo[*d*]imidazol-2-yl)-2-methylbutyl)isoindoline-1,3-dione (6z).

It was obtained as white solid. mp: 117-119 °C; 57% yield; ^{1}H NMR (400 MHz, $CDCl_3$): δ 0.77-0.93 (6H, m), 1.03-1.14 (1H, m), 1.43-1.52 (1H, m), 2.59 (1H, s), 3.33-3.44 (1H, m), 5.38-5.54 (2H, m), 5.65-5.74 (1H, m), 7.22-7.23 (2H, m), 7.34-7.36 (1H, m), 7.42-7.44 (1H, m), 7.55-7.58 (2H, m), 7.67-7.70 (2H, m), 7.86 (1H, s); ^{13}C NMR (100 MHz, $CDCl_3$): δ 10.38, 10.89, 15.30, 16.70, 25.21, 26.74, 33.69, 34.16, 39.08, 40.70, 51.36, 51.54, 109.85, 120.39, 122.67, 123.42, 131.20, 134.10, 134.51, 142.11, 150.20, 150.34, 167.63; HRMS calculated for $C_{23}H_{22}N_6O_2$: 415.1870; found $[M+H]^+$: 415.1892.

Ethyl-(*R*)-2-(4-((2-(1-(1,3-dioxoisoindolin-2-yl)-2-methylpropyl)-1*H*-benzo[*d*]imidazol-1-yl)methyl)-1*H*-1,2,3-triazol-1-yl)acetate (6a').

It was obtained as brown solid. mp: 176-178 °C; 81% yield; ^{1}H NMR (400 MHz, $CDCl_3$): δ 1.05 (3H, d, *J* = 6.10 Hz), 1.10 (3H, d, *J* = 6.87 Hz), 1.26 (3H, t, *J* = 6.87 Hz, *J* = 7.63 Hz), 3.60-3.69 (1H, m), 4.20 (2H, q, *J* = 6.87 Hz, *J* = 14.50 Hz), 4.86 (2H, dd, *J* = 17.55 Hz, *J* = 47.30 Hz), 5.34 (1H, d, *J* = 10.68 Hz), 5.61 (2H, dd, *J* = 16.78 Hz, *J* = 66.38 Hz), 7.27-7.32 (3H, m), 7.37-7.39 (1H, m), 7.71-7.73 (2H, m), 7.79-7.82 (2H, m), 7.90-7.92 (1H, m); ^{13}C NMR (100 MHz, $CDCl_3$): δ 13.93, 19.35, 20.87, 28.33, 39.24, 50.58, 53.04, 62.37, 109.62, 120.55, 122.48, 122.85, 123.36, 131.28, 134.05, 134.63, 142.38, 143.57, 150.09, 165.63, 167.42; HRMS calculated for $C_{26}H_{26}N_6O_4$: 487.2090; found $[M+H]^+$: 487.2102.

(*R*)-2-(4-((2-(1-(1,3-dioxoisoindolin-2-yl)-2-methylpropyl)-1*H*-benzo[d]imidazol-1-yl)methyl)-1*H*-1,2,3-triazol-1-yl)acetic acid (6b').

It was obtained as off white solid. mp: > 300 °C; 74% yield; ^{1}H NMR (400 MHz, D_2O): δ 0.58 (3H, d, *J* = 6.10 Hz), 1.02 (3H, d, *J* = 6.87 Hz), 2.27-2.36 (1H, m), 4.78 (2H, s), 5.04 (1H, d, *J* = 9.16 Hz) 5.66 (2H, dd, *J* = 16.87 Hz, *J* = 58.75 Hz) 7.09 (1H, d, *J* = 7.63 Hz), 7.27-7.29 (3H, m), 7.35-7.43 (2H, m), 7.50-7.53 (1H, m), 7.60 (1H, d, *J* = 8.39 Hz), 7.76 (1H, m); ^{13}C NMR (100 MHz, D_2O): δ 18.46, 18.87, 31.58, 38.95, 52.59, 53.12, 111.10, 118.23, 123.29, 123.73, 124.99, 127.24, 127.76, 129.08, 130.65, 133.18, 134.38, 140.56, 143.15, 154.23, 171.98, 172.98; HRMS calculated for $C_{24}H_{22}N_6O_4$: 459.1777; found $[M+H]^+$: 459.1777.

(2*S*,4*R*,5*S*)-2-(acetoxymethyl)-6-(4-((2-((*R*)-1-(1,3-dioxoisoindolin-2-yl)-2-methylpropyl)-1*H*-benzo[*d*]imidazol-1-yl)methyl)-1*H*-1,2,3-triazol-1-yl)tetrahydro-2*H*-pyran-3,4,5-triyl triacetate (6c').

It was obtained as white solid. mp: 135-137 °C; 64% yield; ^{1}H NMR (400 MHz, $CDCl_3$): δ 0.99-1.05 (6H, m), 1.74 (3H, s), 1.98-2.06 (9H, m), 3.58-3.69 (1H, m) 3.87-3.91 (1H, m), 4.05-4.11 (1H, m), 4.19-4.24 (1H, m), 5.11-5.21 (2H, m), 5.28-5.33 (2H, m), 5.41-5.49 (1H, m), 5.59-5.71 (2H, m),

7.23-7.27 (3H, m), 7.32-7.35 (1H, m), 7.48-7.60 (1H, m), 7.67-7.72 (2H, m), 7.77-7.88 (2H, m); ^{13}C NMR (100 MHz, $CDCl_3$): δ 15.21, 19.36, 19.38, 19.95, 20.03, 20.43, 20.46, 20.63, 20.67, 28.25, 28.34, 31.52, 38.72, 38.95, 53.03, 53.06, 61.48, 65.80, 67.52, 69.88, 70.03, 72.29, 72.47, 74.95, 75.02, 85.46, 85.54, 109.73, 109.82, 120.22, 120.58, 122.46, 122.49, 123.34, 123.59, 131.31, 131.49, 134.06, 134.15, 134.46, 134.54, 142.35, 143.39, 143.66, 150.08, 167.62, 167.80, 168.63, 168.76, 169.26, 169.30, 169.80, 170.41; HRMS calculated for $C_{36}H_{38}N_6O_{11}$: 731.2673; found $[M+H]^+$: 731.2683.

2-((1*R*)-2-methyl-1-(1-((1-((3*S*,4*R*,6*S*)-3,4,5-trihydroxy-6-(hydroxymethyl)tetrahydro-2*H*-pyran-2-yl)-1*H*-1,2,3-triazol-4-yl)methyl)-1*H*-benzo[d]imidazol-2-yl)propyl)isoindoline-1,3-dione (6d').

It was obtained as off white solid. mp: 297-299 °C; 55% yield; ^{1}H NMR (400 MHz, D_2O): δ 0.56-0.59 (3H, m), 1.00-1.02 (3H, m), 2.27-2.34 (1H, m) 3.38-3.44 (1H, m), 3.48-3.59 (3H, m), 3.67-3.75 (2H, m), 5.04 (1H, t, *J* = 9.16 Hz), 5.52 (1H, d, *J* = 9.16 Hz), 5.75 (2H, dd, *J* = 16.78 Hz, *J* = 42.72 Hz), 7.13-7.16 (1H, m), 7.27-7.31 (2H, m), 7.35-7.39 (1H, m), 7.43-7.45 (1H, m,), 7.49-7.54 (1H, m), 7.58-7.63 (1H, m), 8.08 (1H, d, *J* = 22.89 Hz); ^{13}C NMR (100 MHz, D_2O): δ 18.46, 18.75, 23.07, 31.80, 52.48, 60.35, 68.86, 72.21, 75.88, 78.79, 87.45, 110.98, 111.02, 118.13, 123.27, 123.44, 123.84, 127.25, 127.88, 129.20, 130.64, 133.30, 133.33, 134.15, 140.28, 154.21, 172.13; HRMS calculated for $C_{28}H_{30}N_6O_7$: 563.2250; found $[M+H]^+$: 563.2255.

(*R*)-2-(1-(1-((1-hexyl-1*H*-1,2,3-triazol-4-yl)methyl)-1*H*-benzo[*d*]imidazol-2-yl)-2-methylpropyl)isoindoline-1,3-dione (6e').

It was obtained as off white solid. mp: 99-101 °C; 75% yield; ^{1}H NMR(400 MHz, $CDCl_3$): δ 0.77 (3H, t, *J* = 6.10 Hz, *J* = 6.87 Hz), 0.94 (3H, d, *J* = 6.10 Hz), 1.01 (3H, d, *J* = 6.10 Hz), 1.10-1.18 (6H, m), 1.49-1.56 (2H, m), 3.48-3.58 (1H, m), 3.82-3.95 (2H, m), 5.22 (1H, d, *J* = 10.68 Hz), 5.47 (2H, dd, *J* = 16.78 Hz, *J* = 52.64 Hz), 7.15-7.26 (4H, m), 7.60-7.62 (2H, m), 7.67-7.70 (2H, m), 7.81-7.83 (1H, m); ^{13}C NMR (100 MHz, $CDCl_3$): δ 13.86, 19.35, 20.95, 22.30, 25.95, 28.33, 29.96, 30.97, 39.48, 50.18, 53.02, 109.62, 120.59, 120.96, 122.47, 123.29, 131.24, 134.09, 134.56, 142.45, 143.18, 150.04, 167.46; HRMS calculated for $C_{28}H_{32}N_6O_2$: 485.2661; found $[M+H]^+$: 485.2658.

Copies of ^{1}H NMR, ^{13}C NMR and HRMS spectra

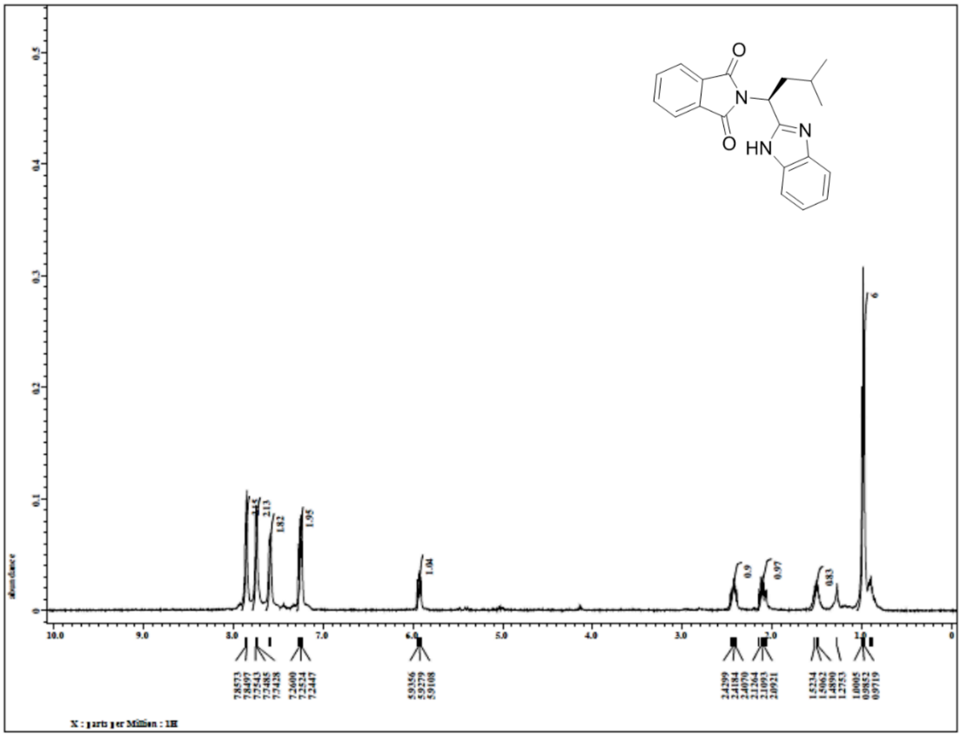

^{1}H NMR spectrum of compound **3a** ($CDCl_3$, 400MHz)

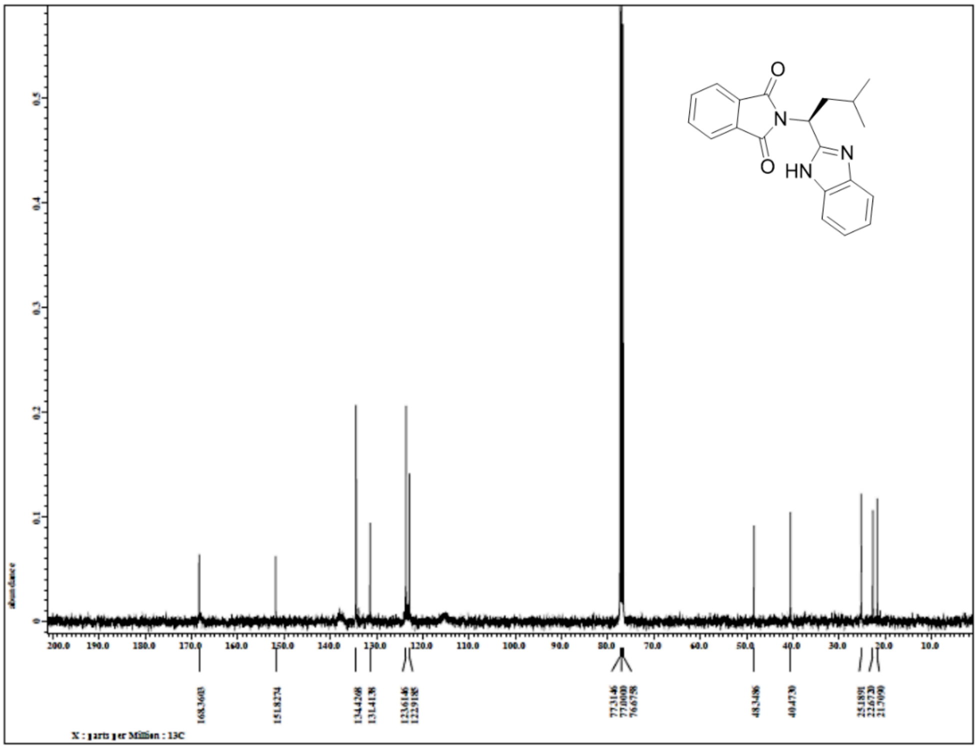

^{13}C NMR spectrum of compound **3a** ($CDCl_3$, 100MHz)

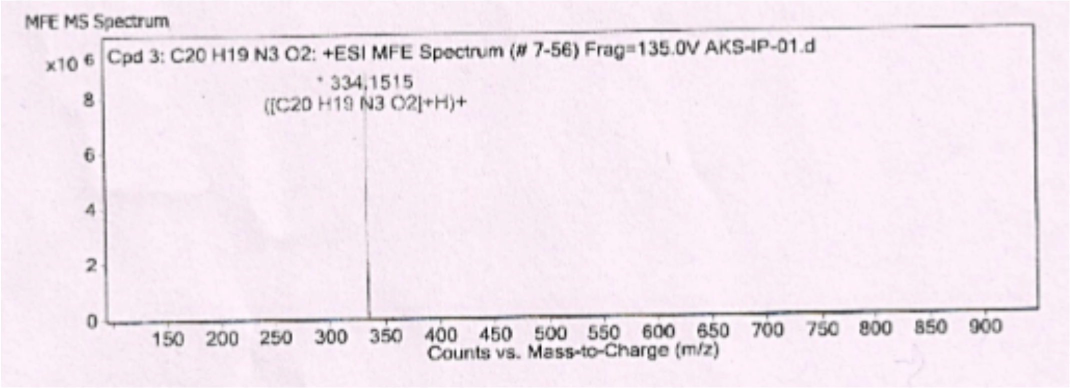

HRMS spectrum of compound **3a**

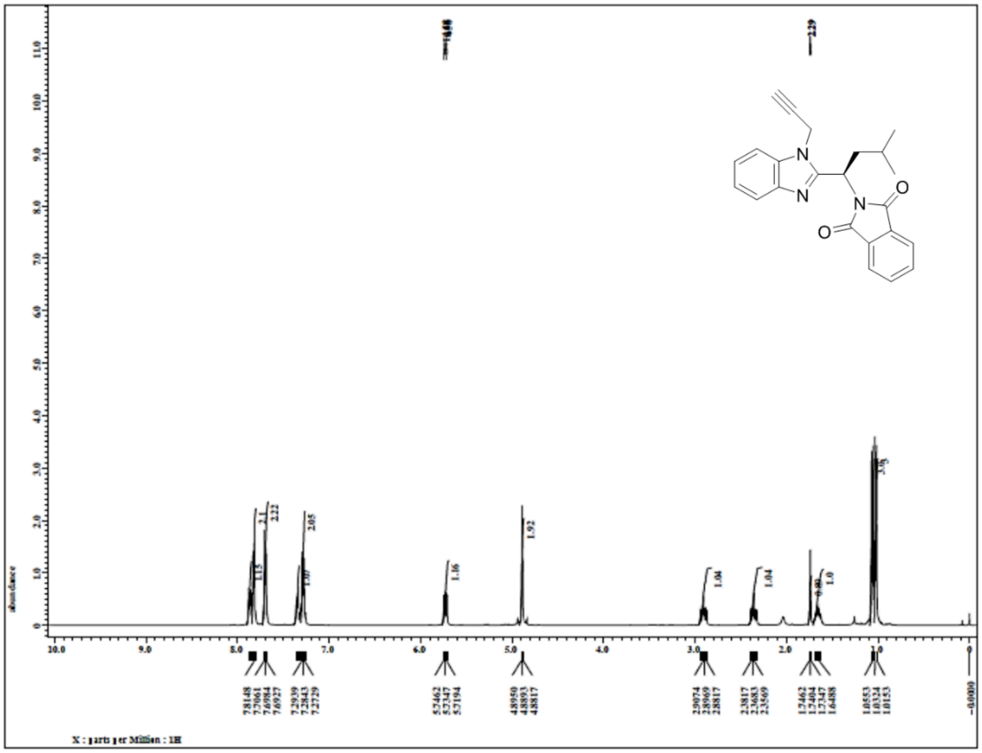

^{1}H NMR spectrum of compound **5a** ($CDCl_3$, 400MHz)

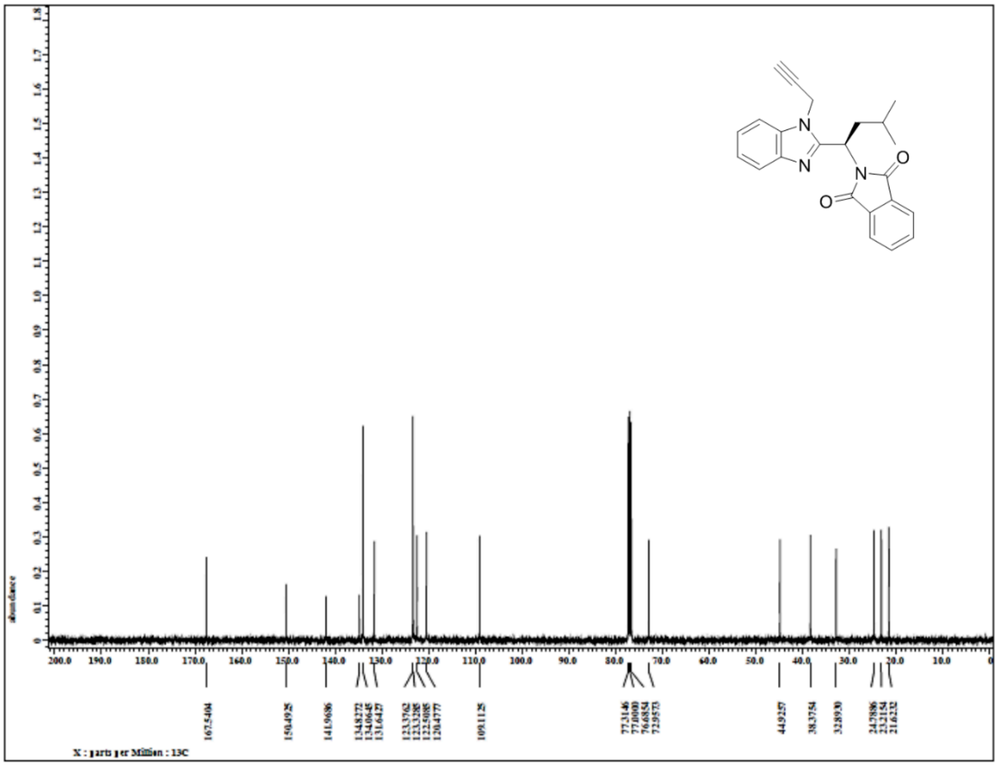

^{13}C NMR spectrum of compound **5a** ($CDCl_3$, 100MHz)

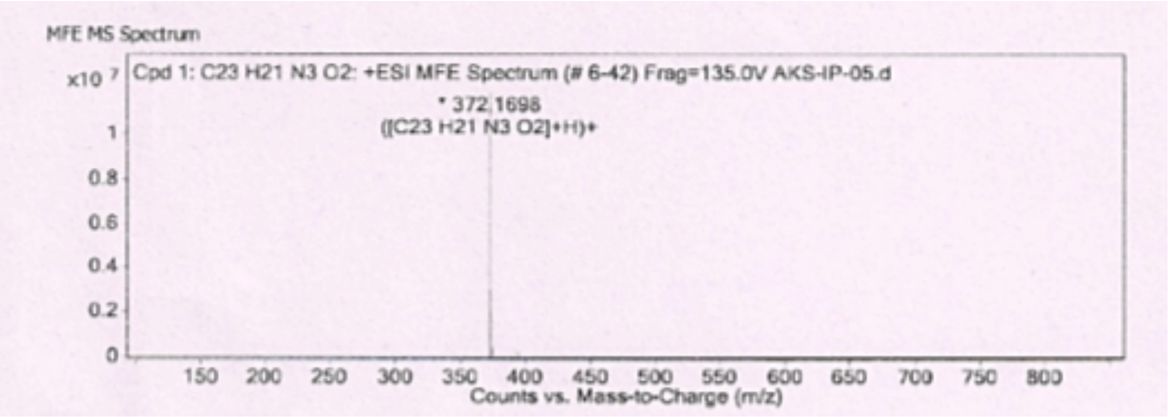

HRMS spectrum of compound **5a**

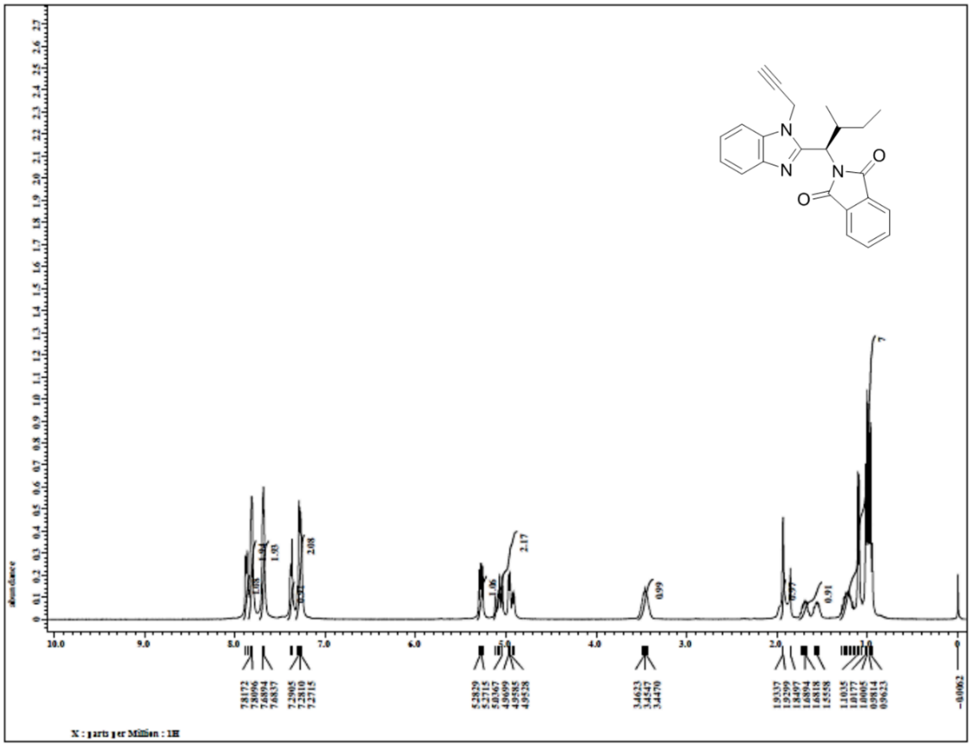

^{1}H NMR spectrum of compound **5b** ($CDCl_3$, 400MHz)

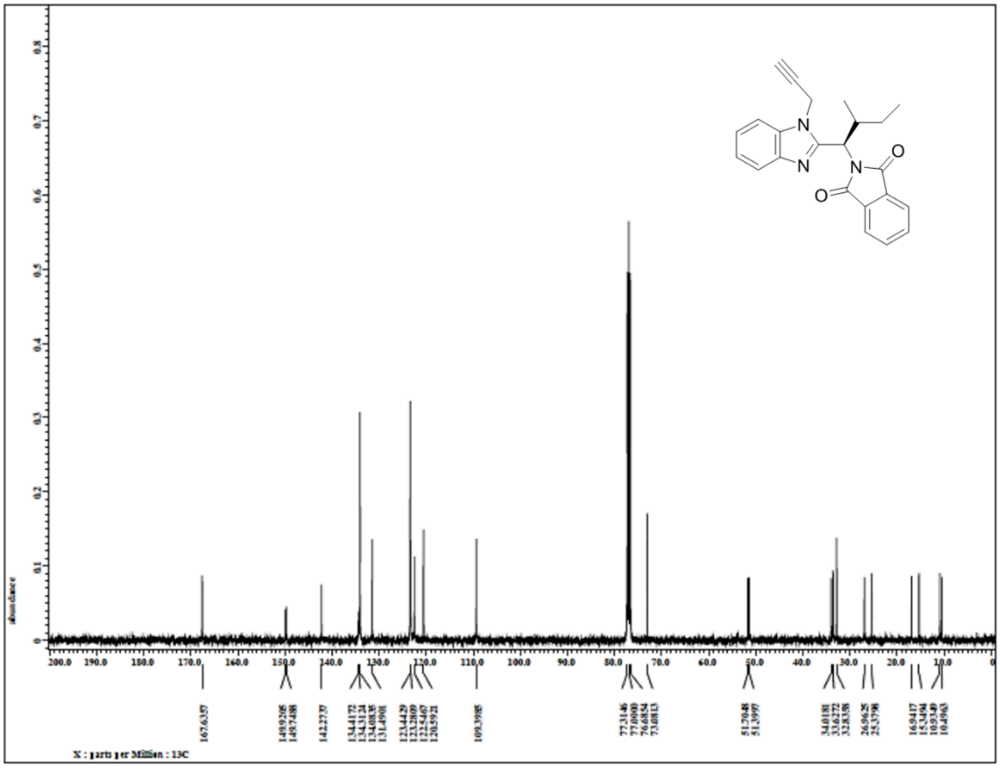

^{13}C NMR spectrum of compound **5b** ($CDCl_3$, 100MHz)

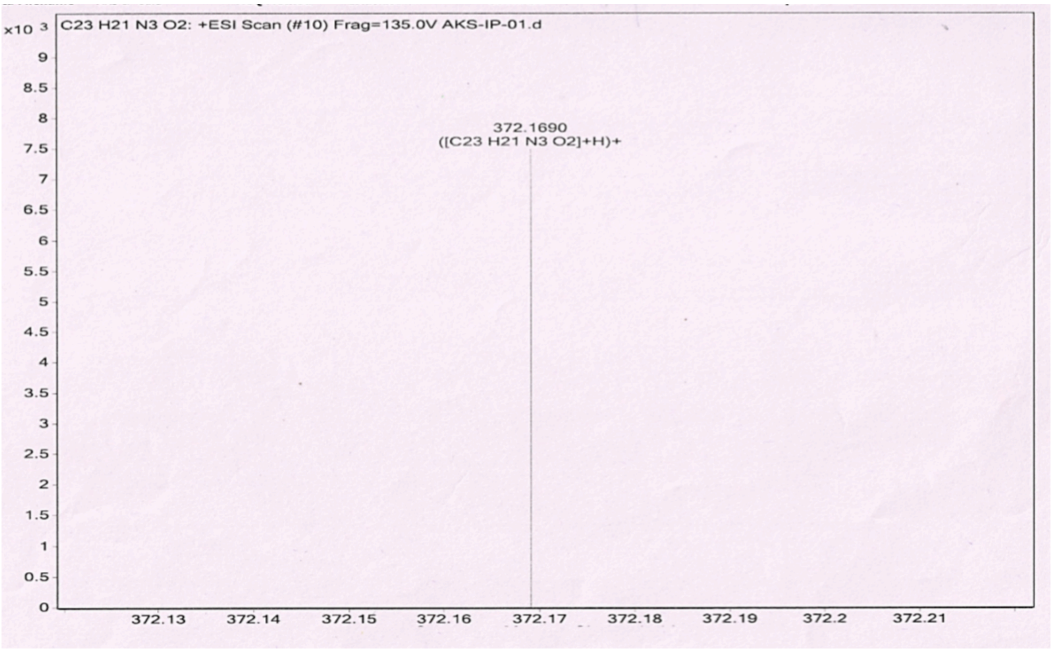

HRMS spectrum of compound **5b**

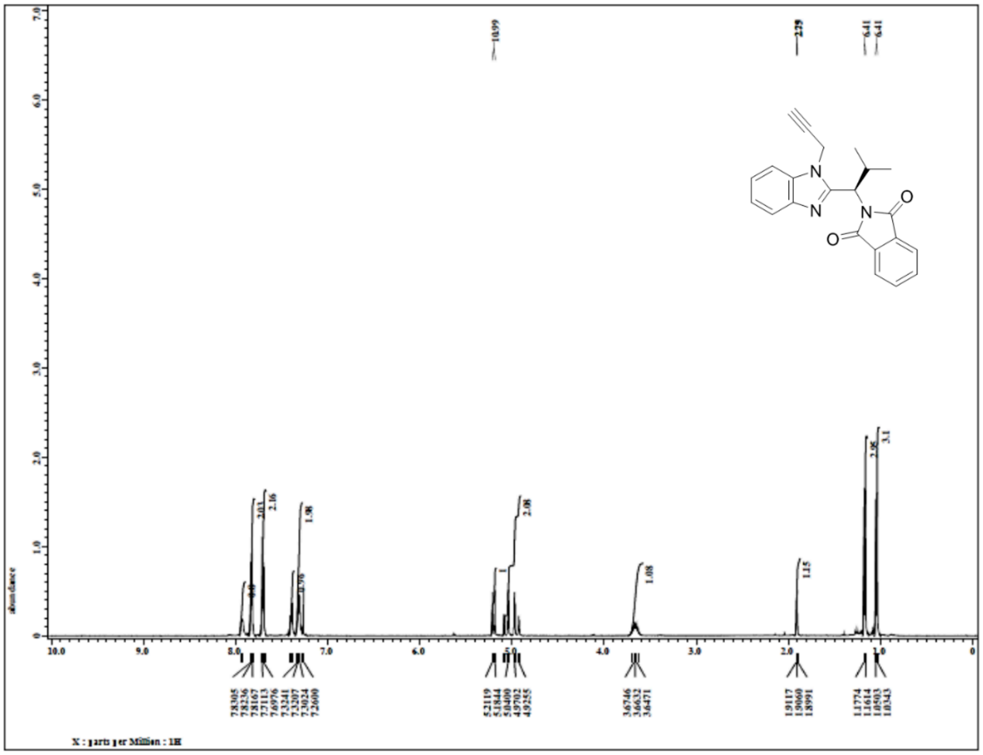

^{1}H NMR spectrum of compound **5c** ($CDCl_3$, 400MHz)

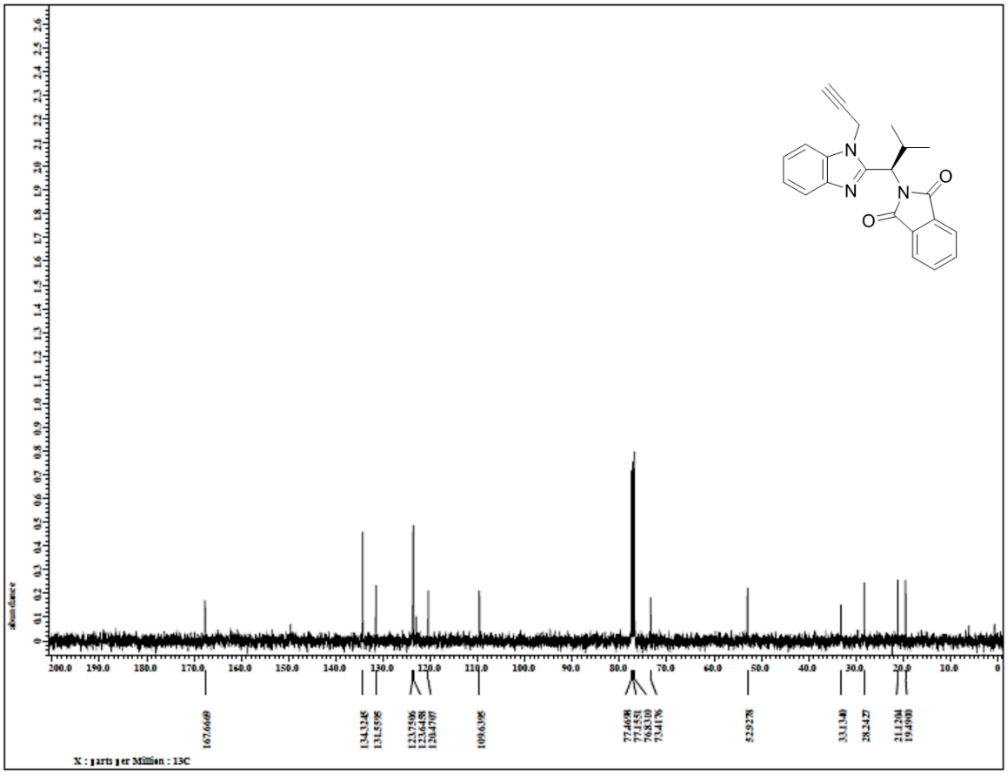

^{13}C NMR spectrum of compound **5c** ($CDCl_3$, 100MHz)

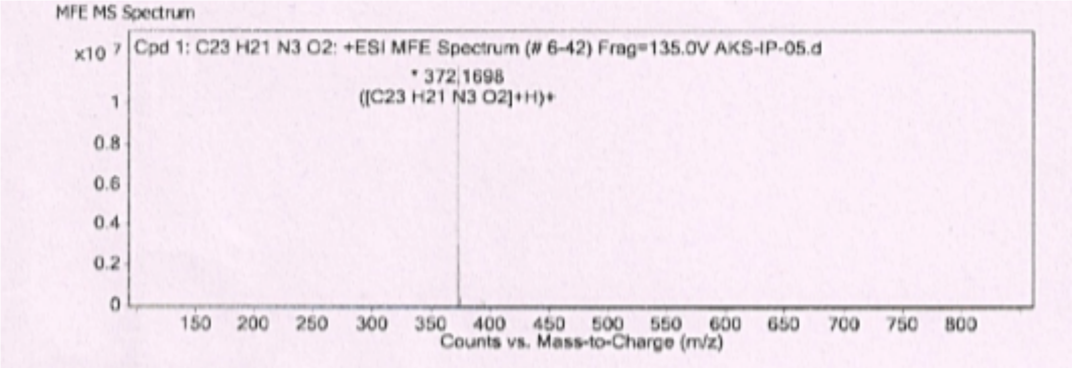

HRMS spectrum of compound **5c**

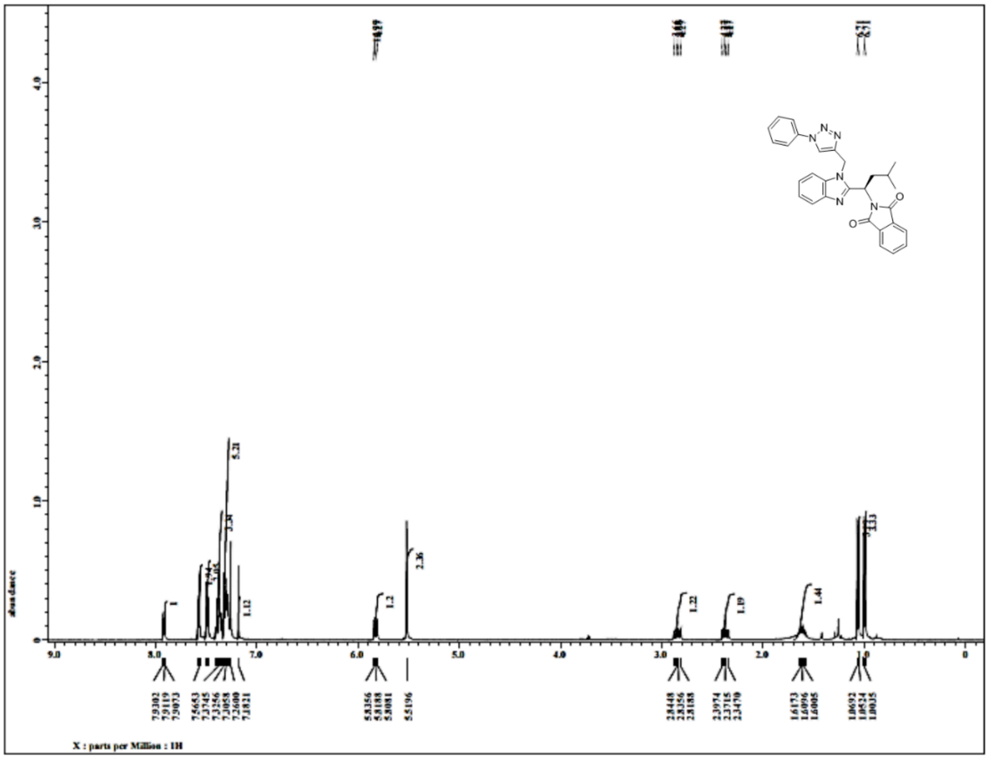

^{1}H NMR spectrum of compound **6a** ($CDCl_3$, 400MHz)

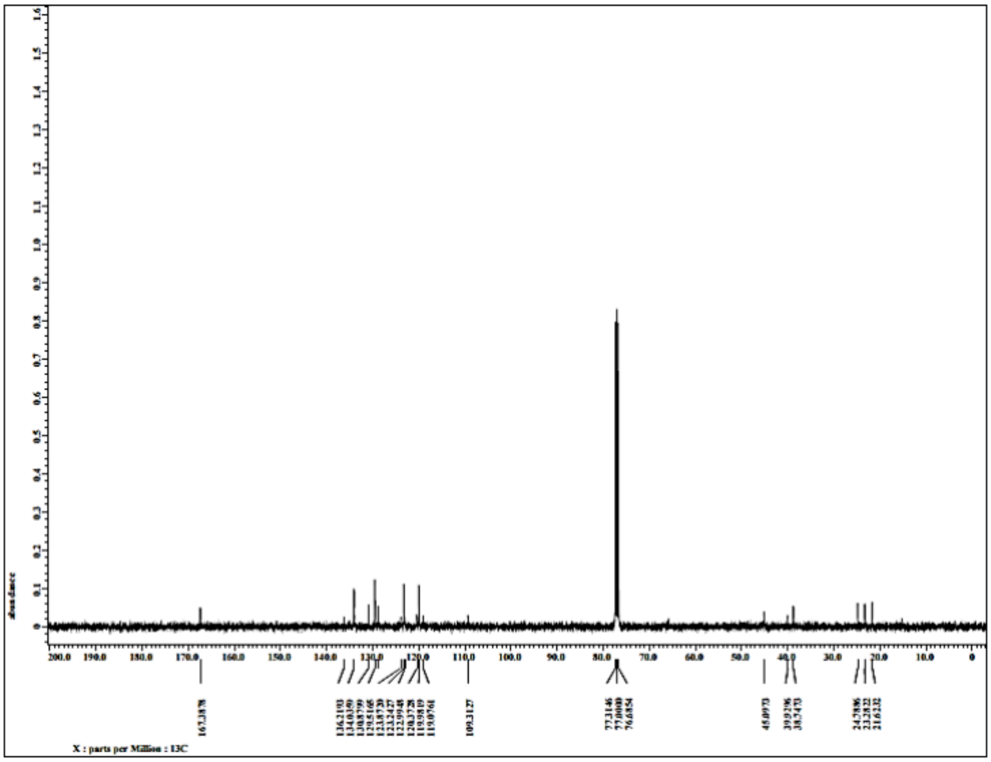

^{13}C NMR spectrum of compound **6a** ($CDCl_3$, 100MHz)

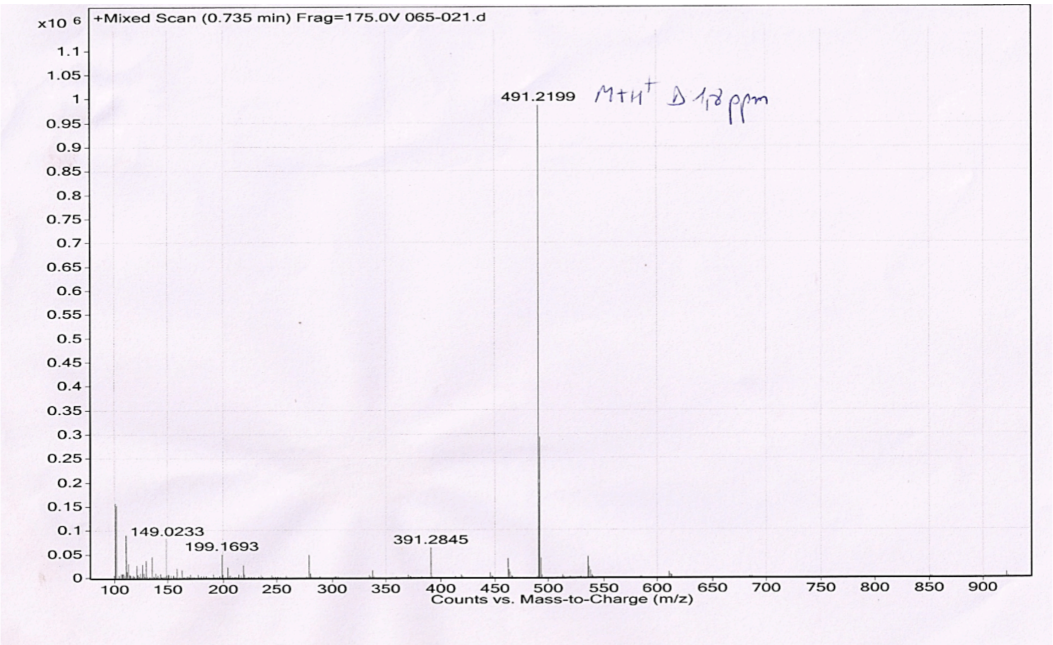

HRMS spectrum of compound **6a**

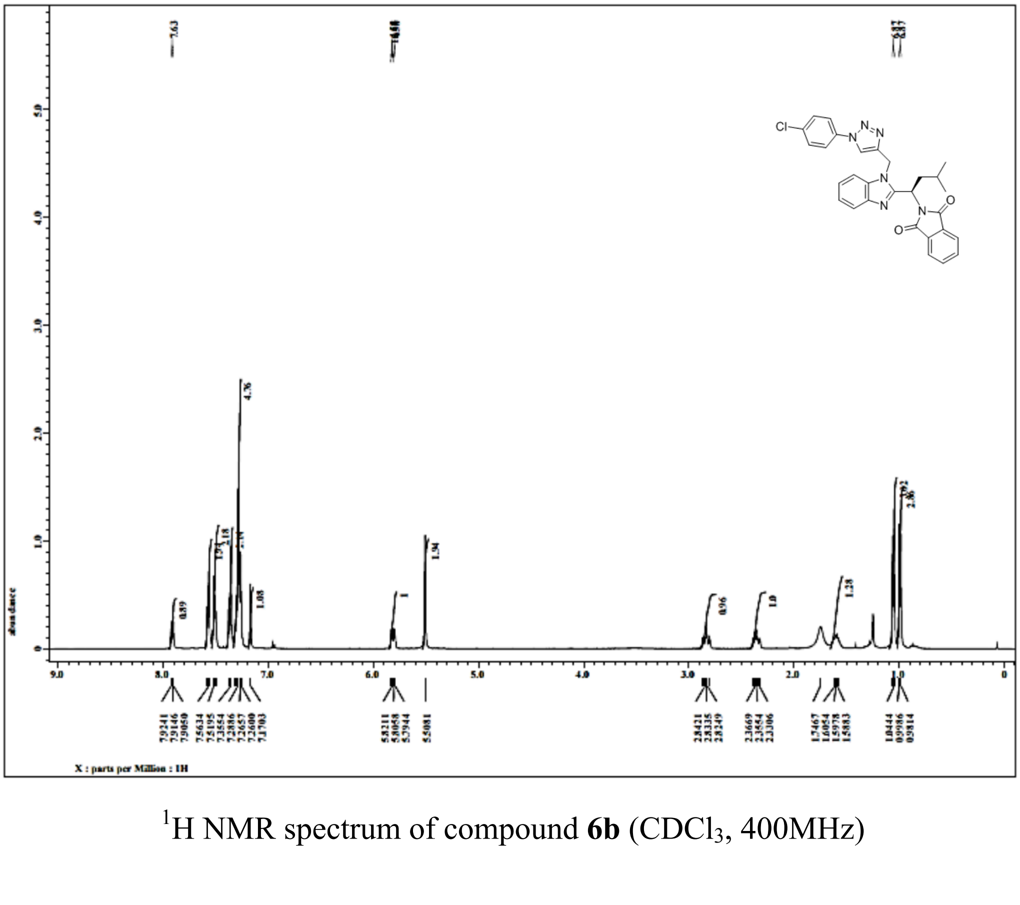

^{1}H NMR spectrum of compound **6b** ($CDCl_3$, 400MHz)

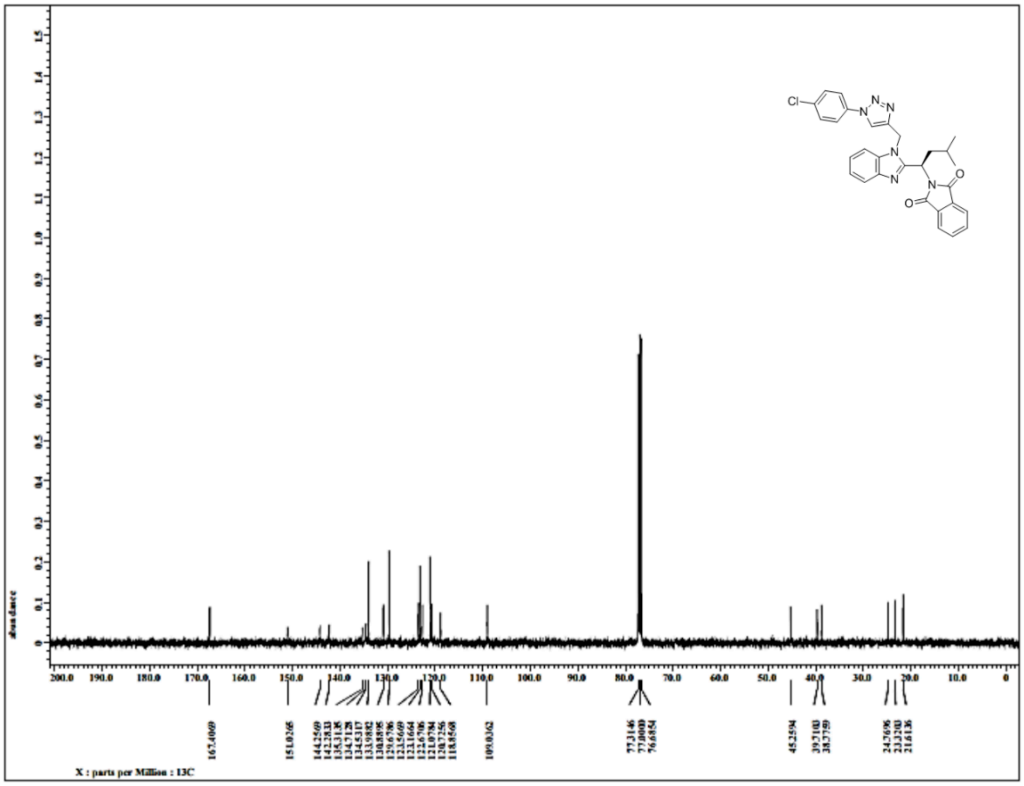

^{13}C NMR spectrum of compound **6b** ($CDCl_3$, 100MHz)

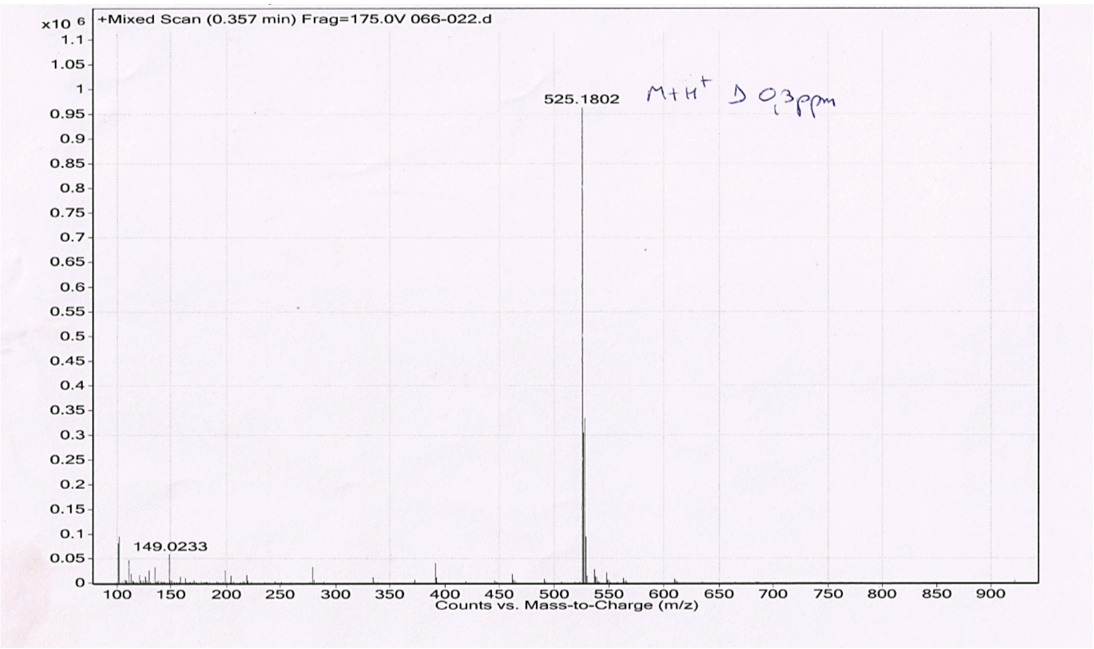

HRMS spectrum of compound **6b**

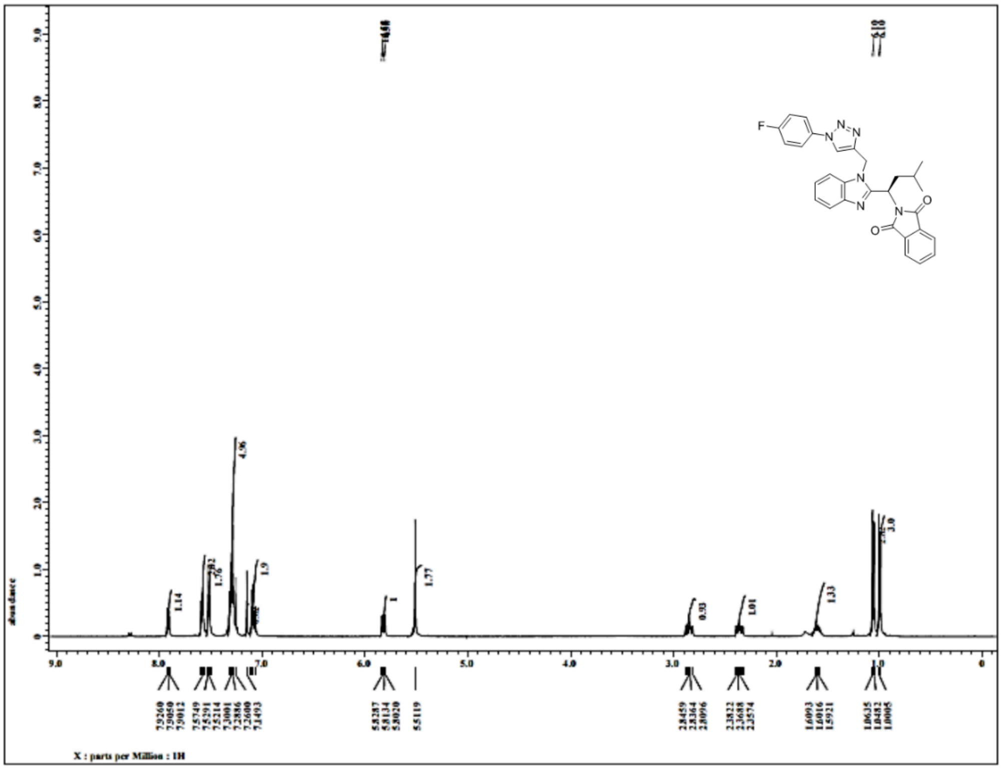

^{1}H NMR spectrum of compound **6c** ($CDCl_3$, 400MHz)

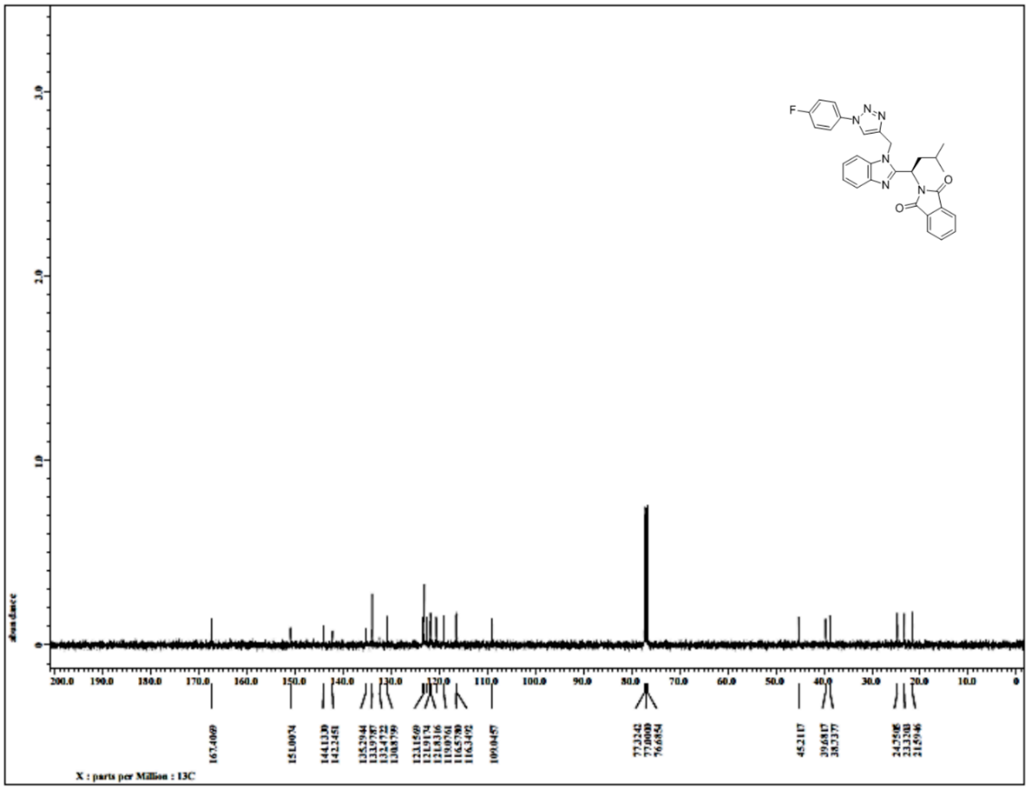

^{13}C NMR spectrum of compound **6c** ($CDCl_3$, 100MHz)

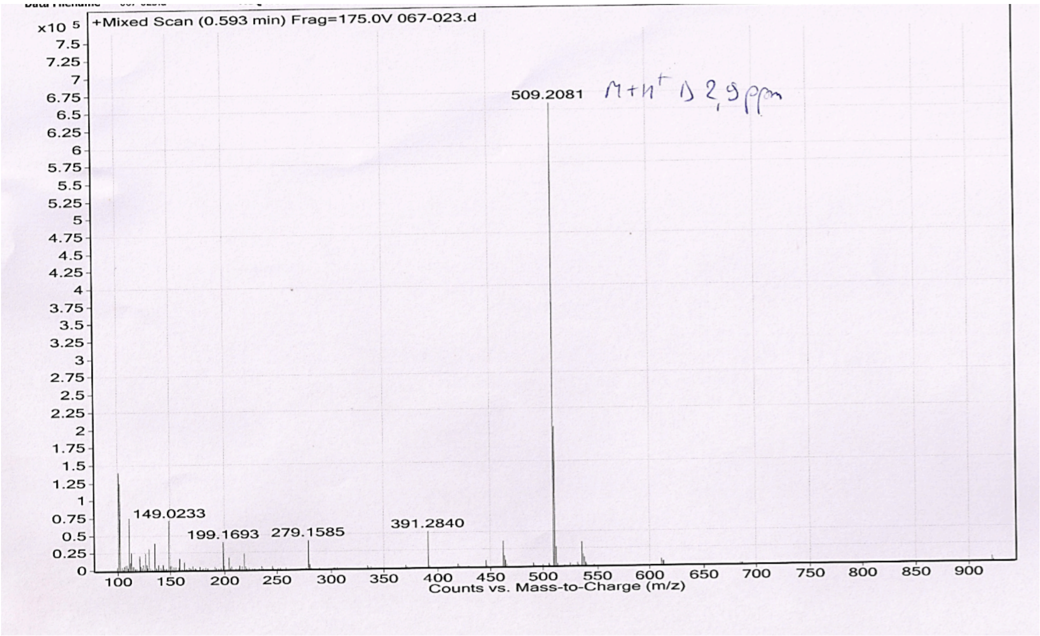

HRMS spectrum of compound **6c**

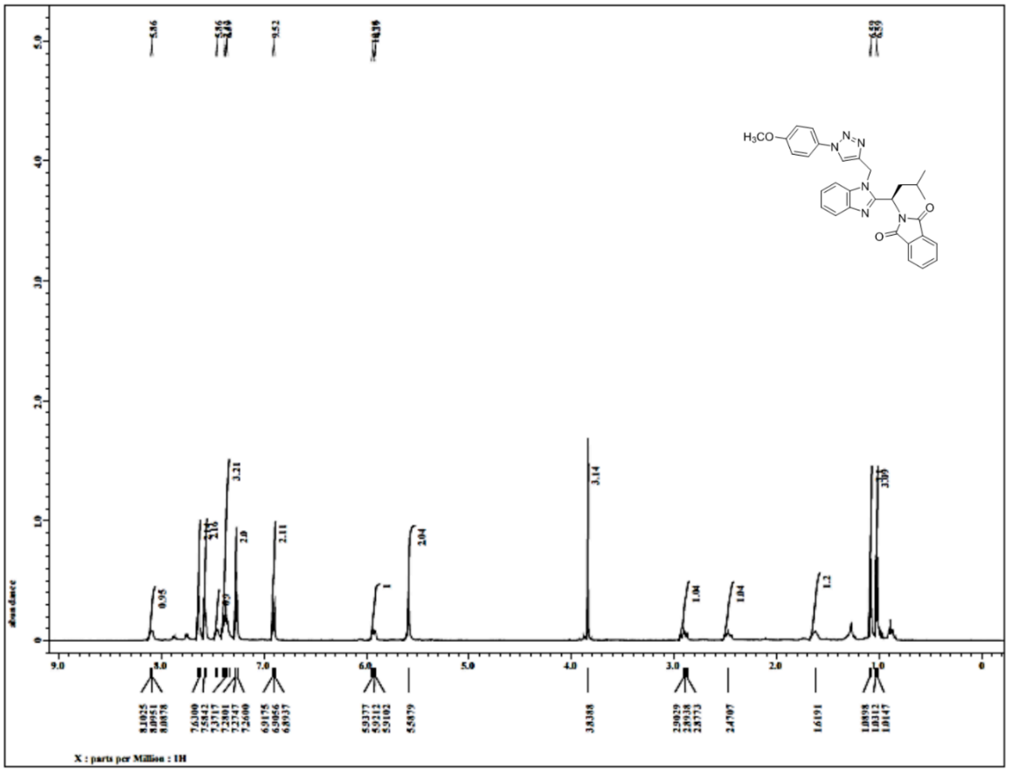

^{1}H NMR spectrum of compound **6d** ($CDCl_3$, 400MHz)

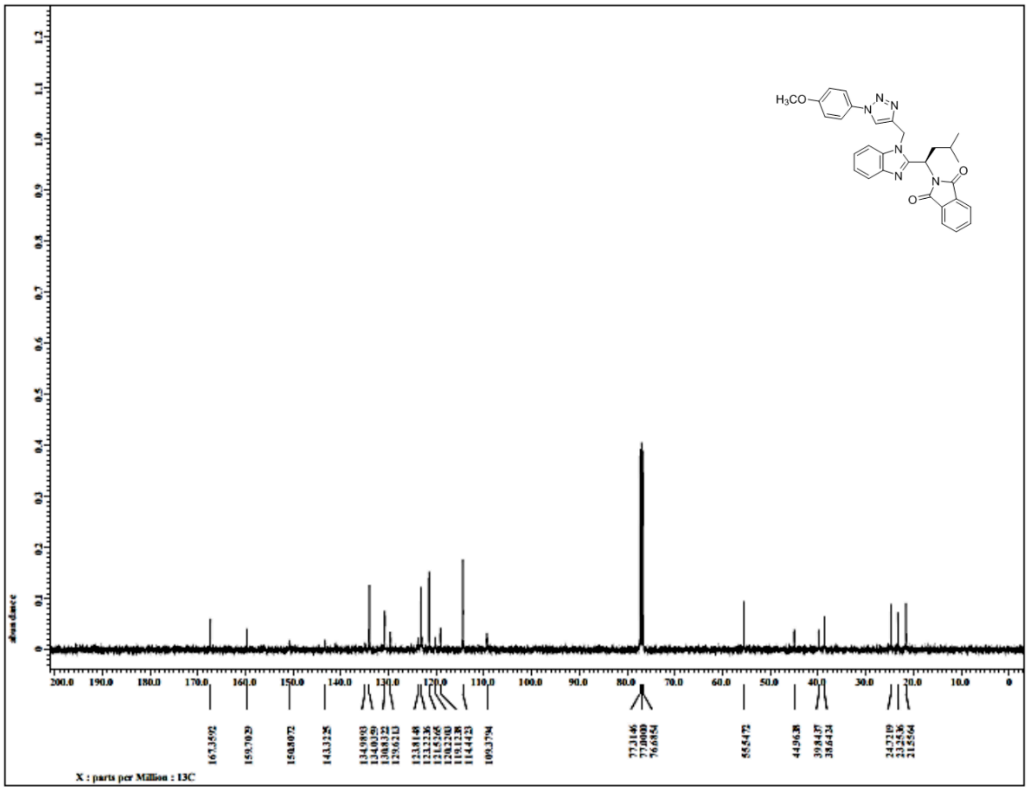

^{13}C NMR spectrum of compound **6d** ($CDCl_3$, 100MHz)

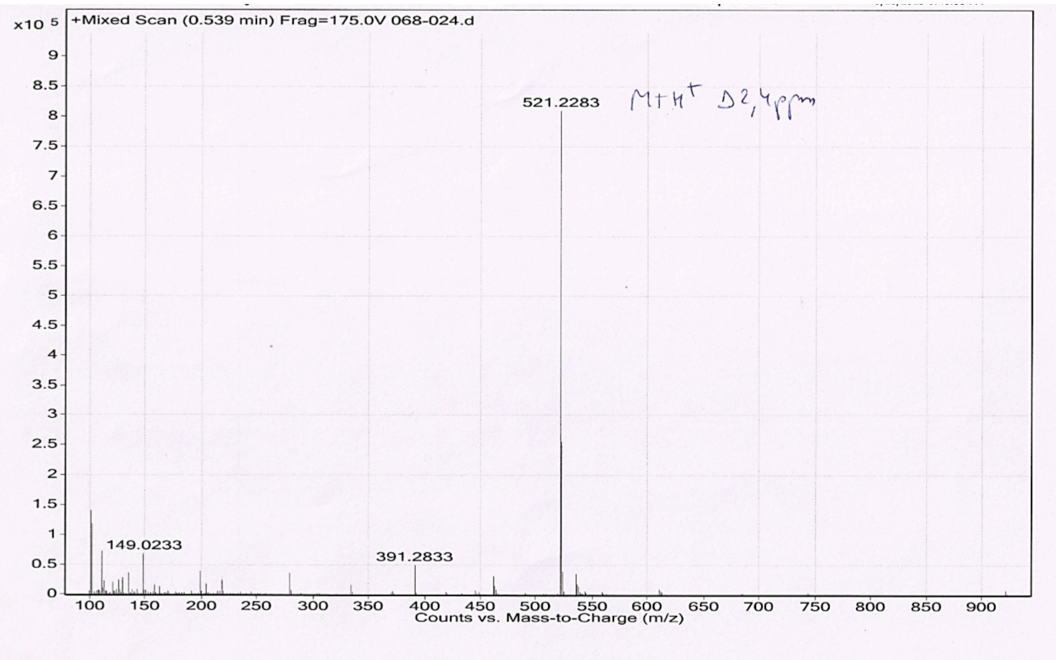

HRMS spectrum of compound **6d**

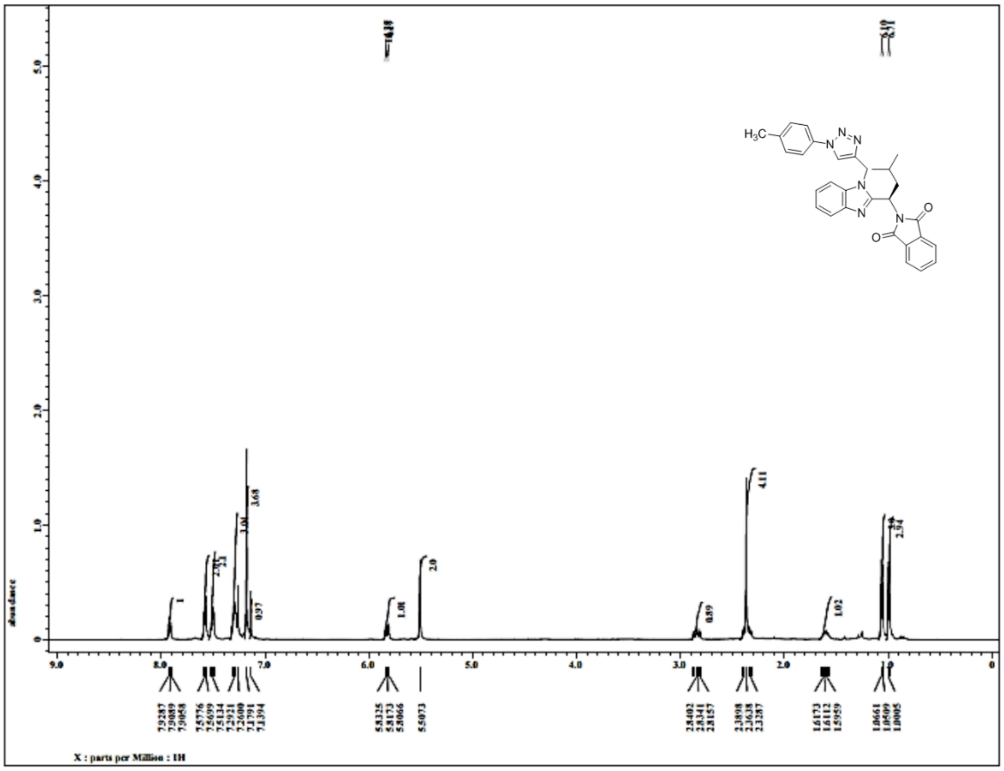

^{1}H NMR spectrum of compound **6e** ($CDCl_3$, 400MHz)

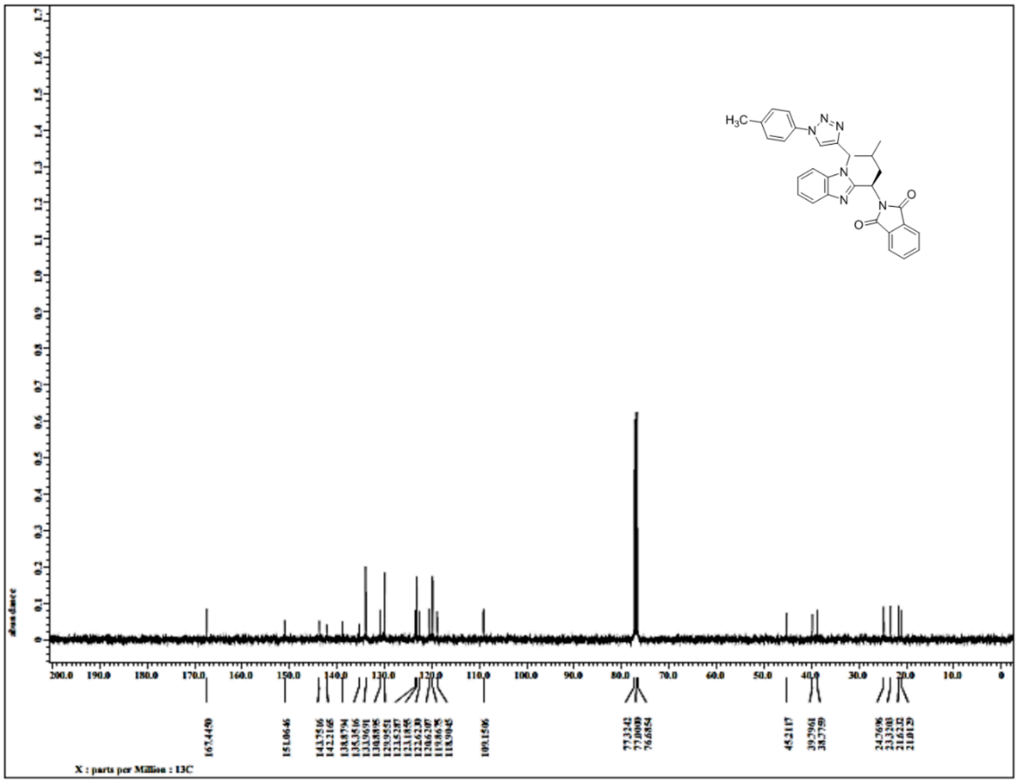

^{13}C NMR spectrum of compound **6e** ($CDCl_3$, 100MHz)

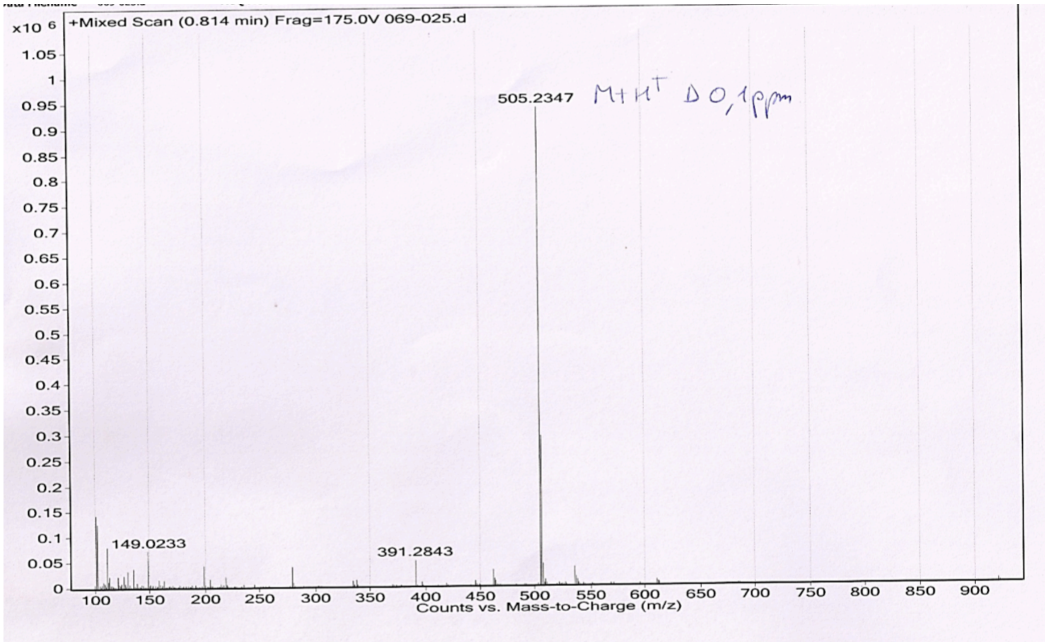

HRMS spectrum of compound **6e**

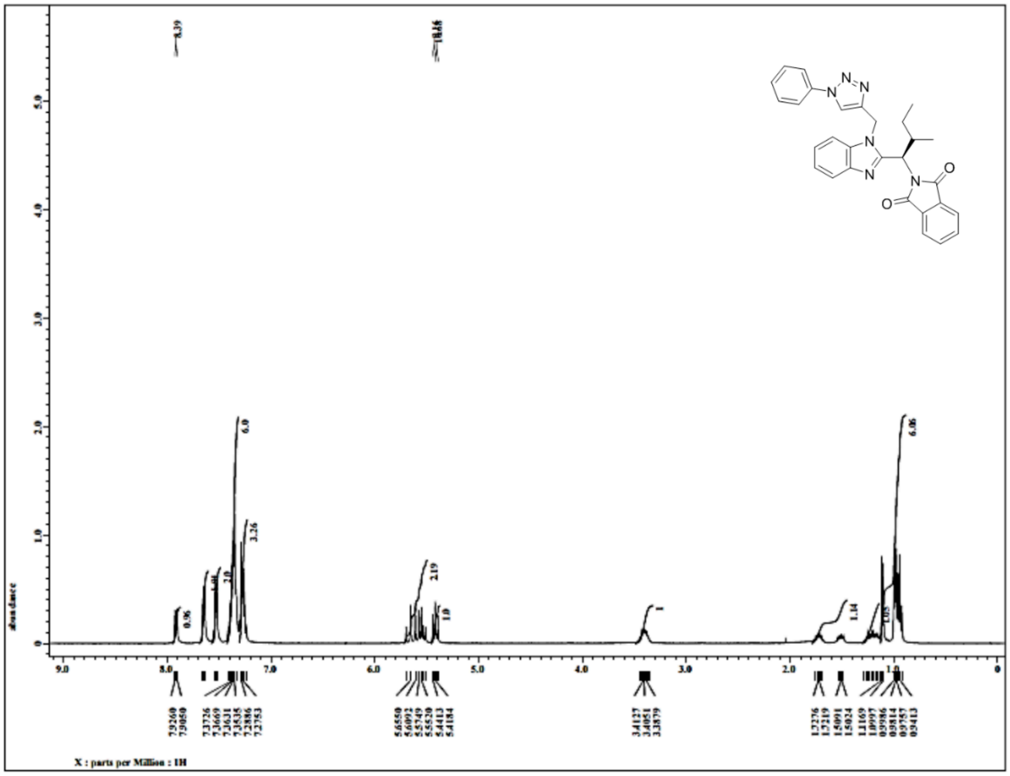

^{1}H NMR spectrum of compound **6f** ($CDCl_3$, 400MHz)

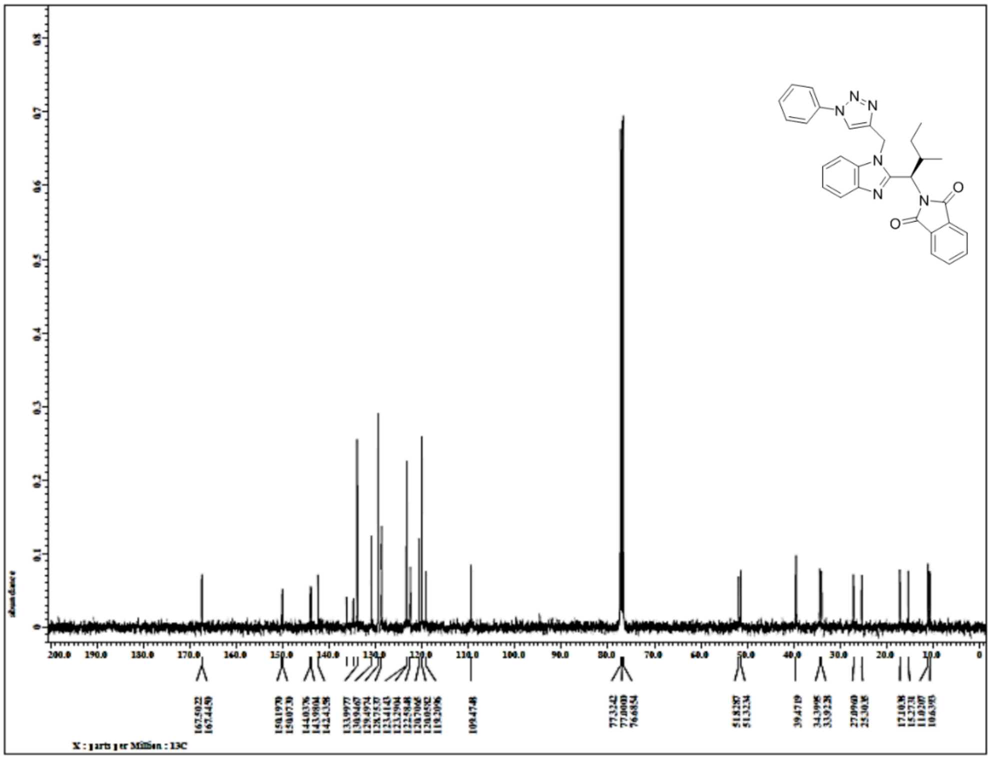

^{13}C NMR spectrum of compound **6f** ($CDCl_3$, 100MHz)

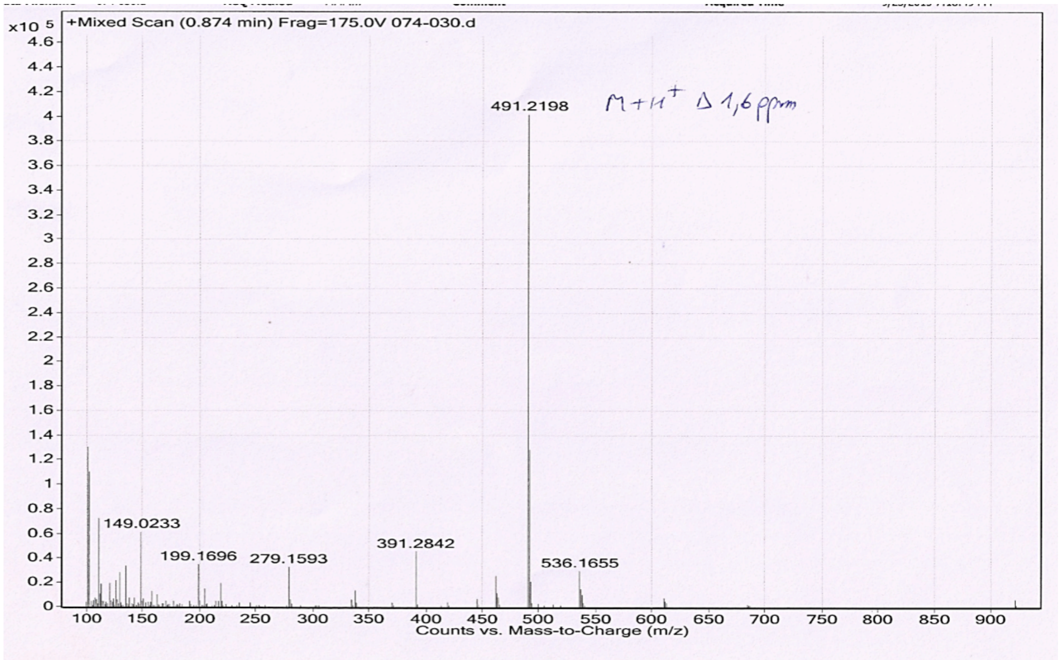

HRMS spectrum of compound **6f**

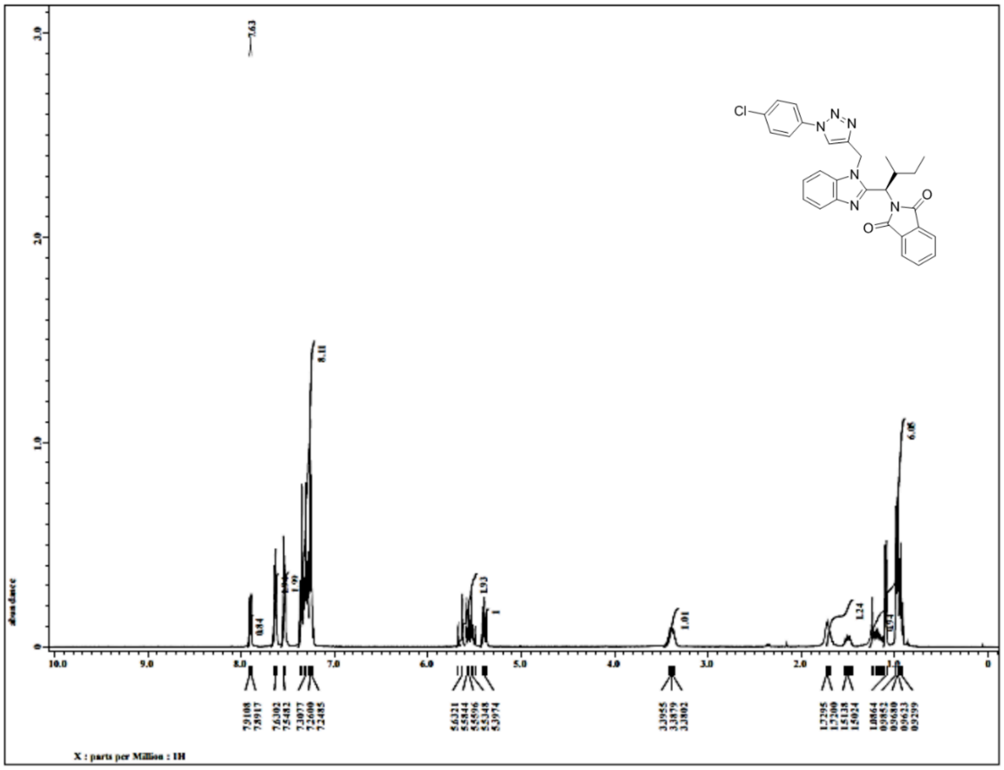

^{1}H NMR spectrum of compound **6g** ($CDCl_3$, 400MHz)

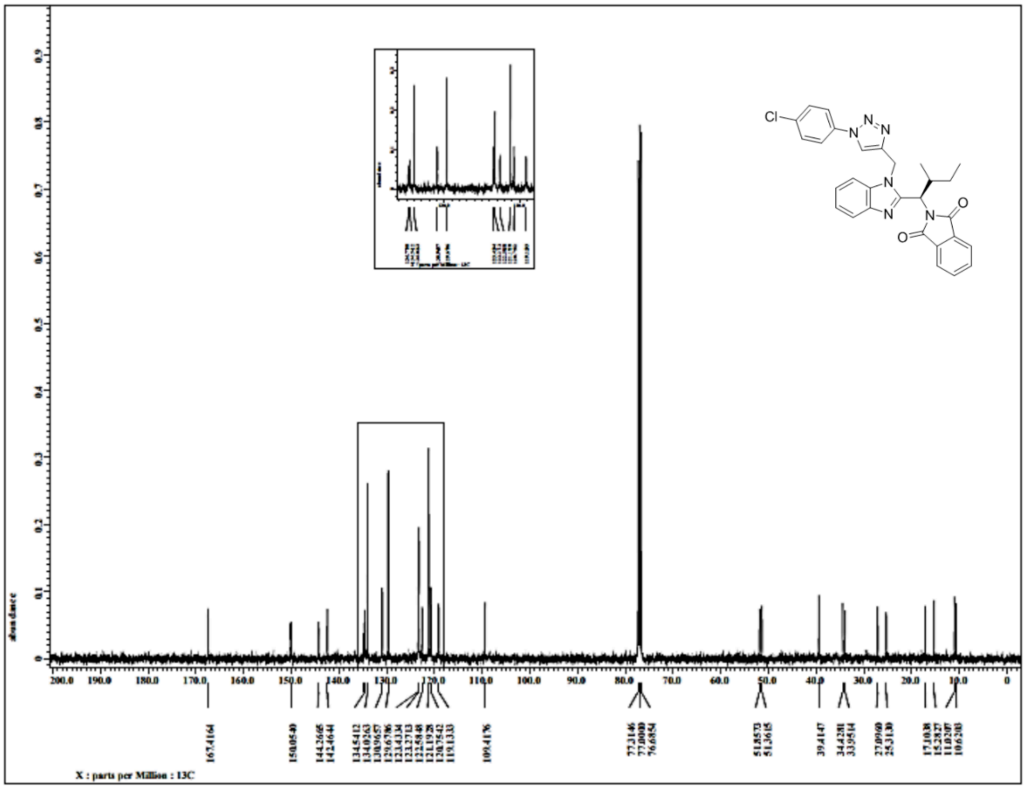

^{13}C NMR spectrum of compound **6g** ($CDCl_3$, 100MHz)

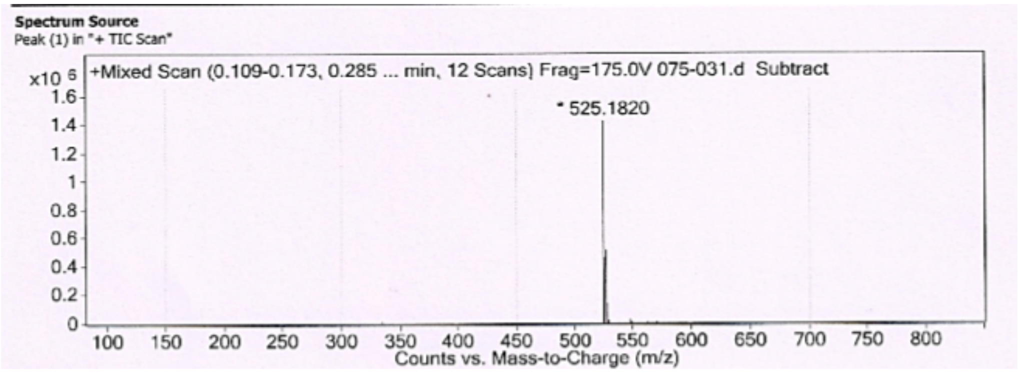

HRMS spectrum of compound **6g**

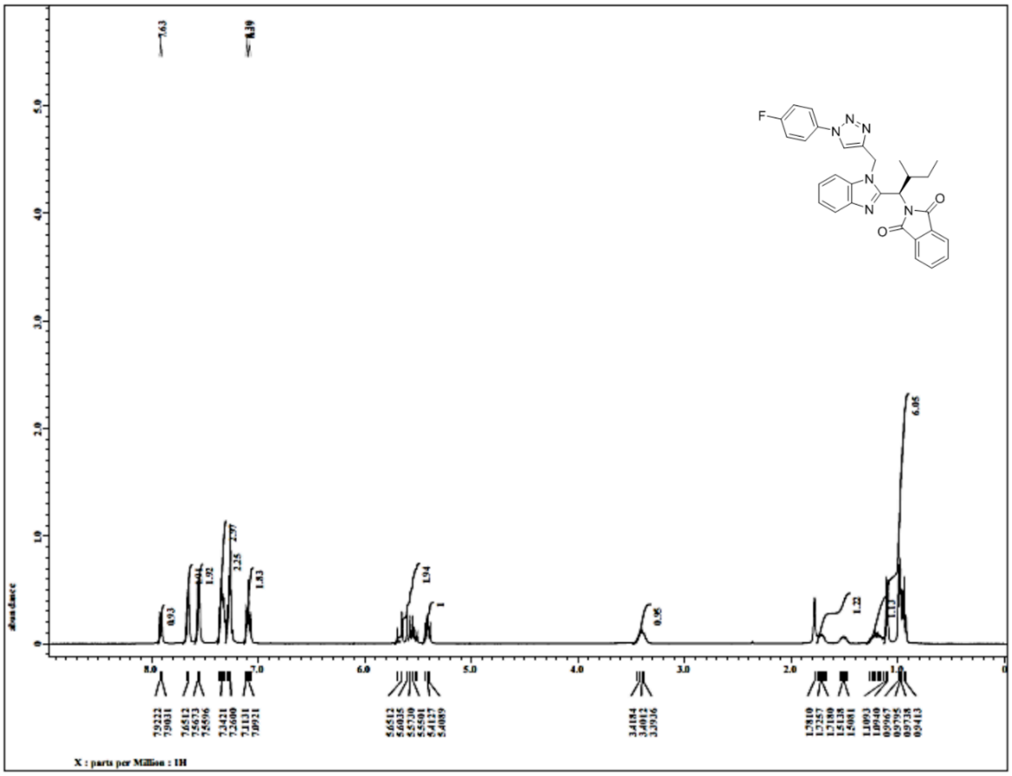

^{1}H NMR spectrum of compound **6h** ($CDCl_3$, 400MHz)

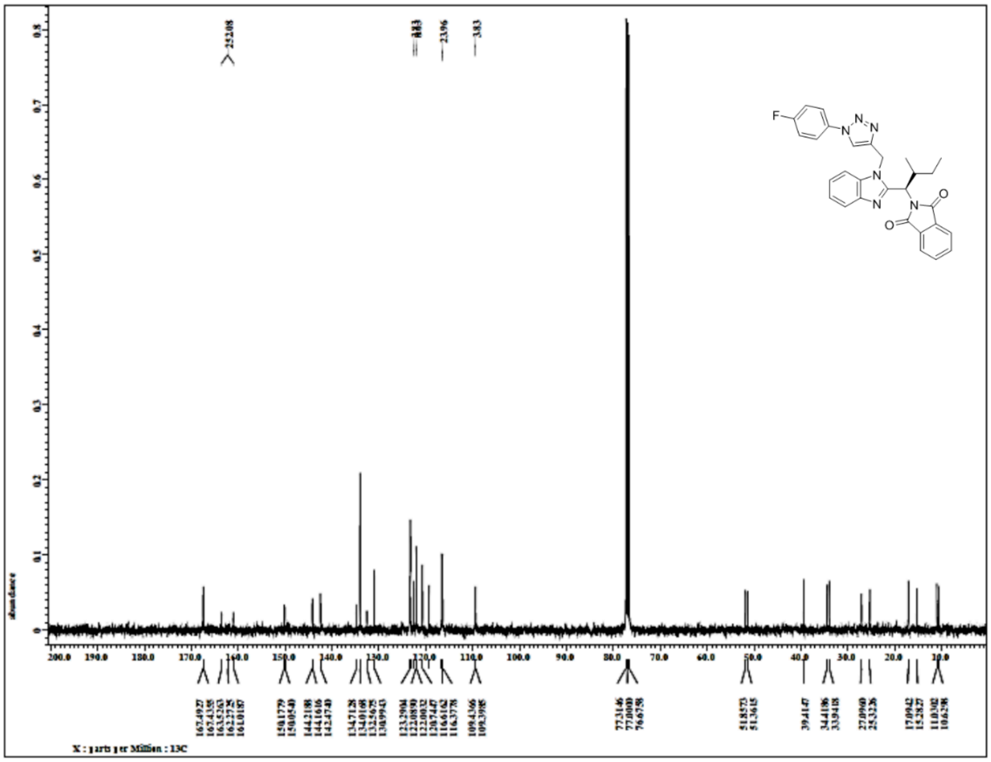

^{13}C NMR spectrum of compound **6h** ($CDCl_3$, 100MHz)

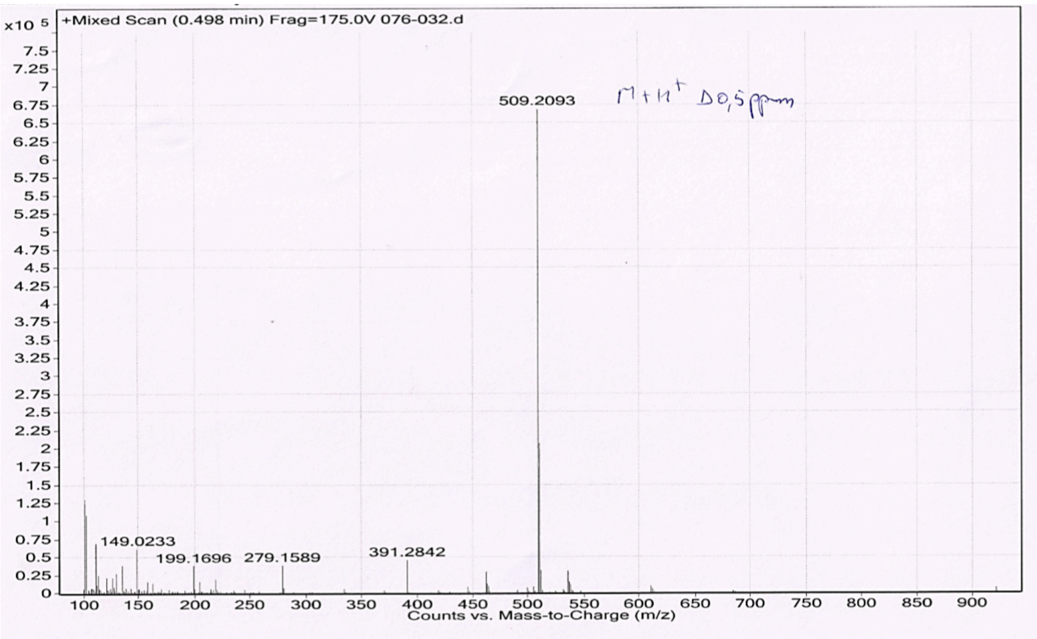

HRMS spectrum of compound **6h**

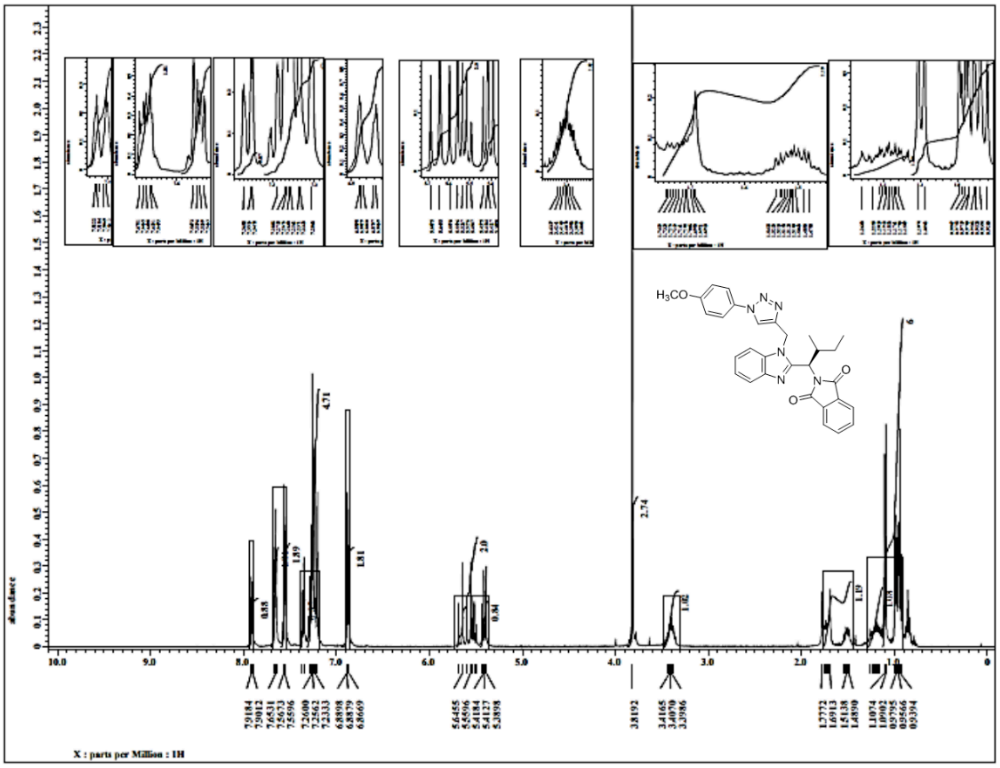

^{1}H NMR spectrum of compound **6i** ($CDCl_3$, 400MHz)

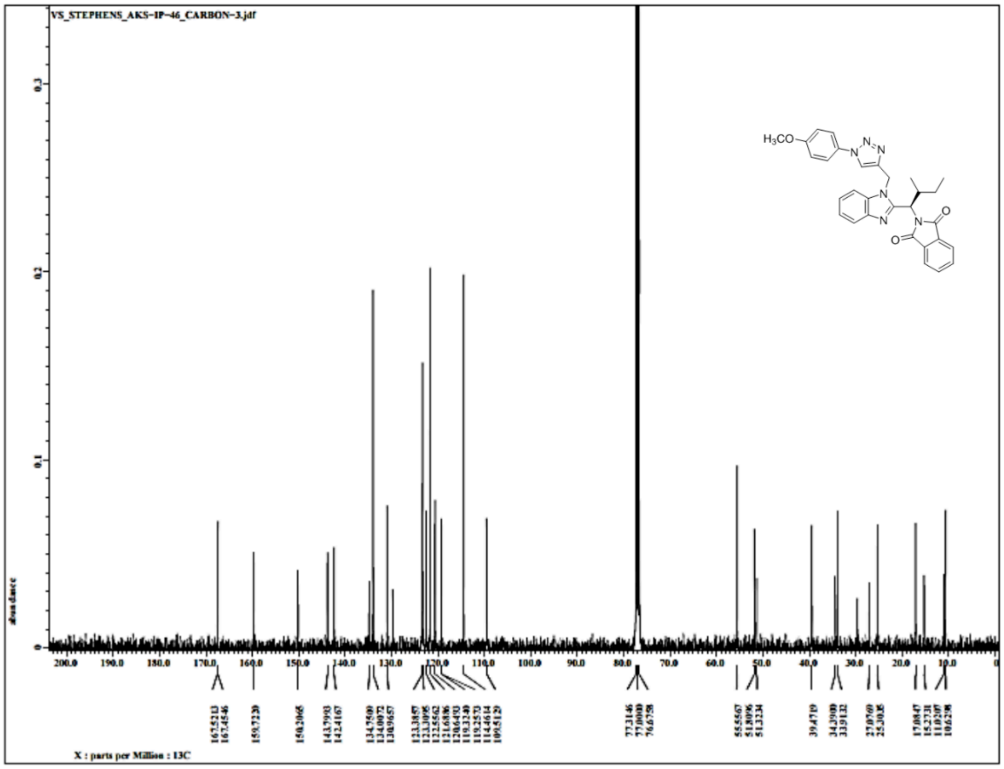

^{13}C NMR spectrum of compound **6i** ($CDCl_3$, 100MHz)

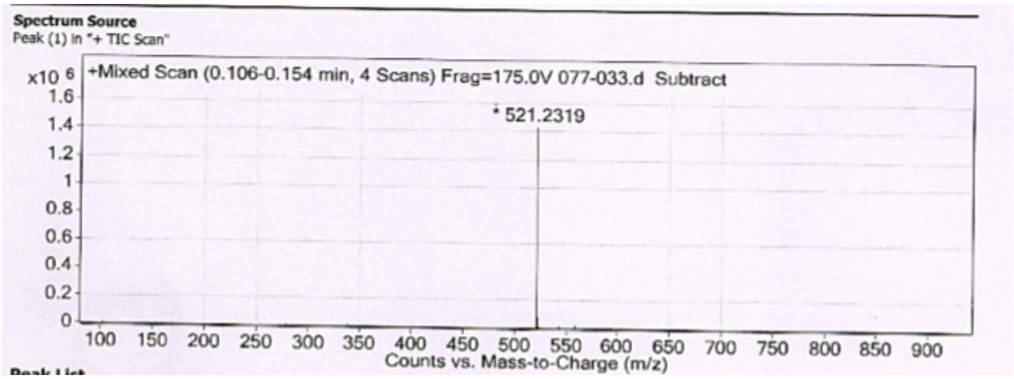

HRMS spectrum of compound **6i**

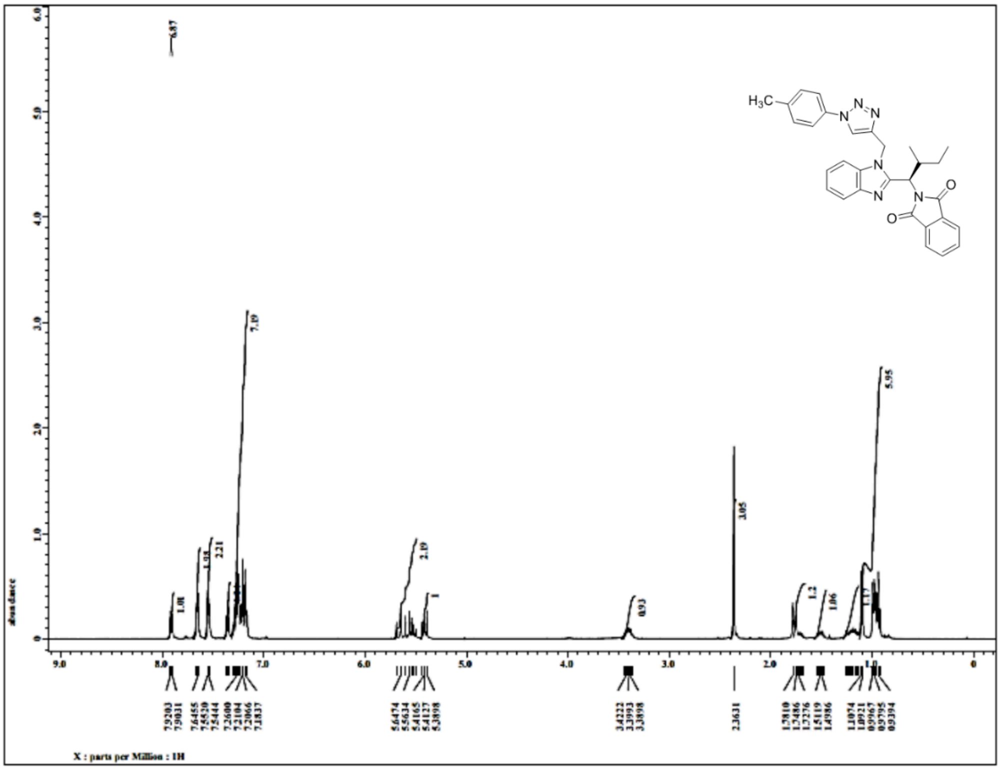

^{1}H NMR spectrum of compound **6j** ($CDCl_3$, 400MHz)

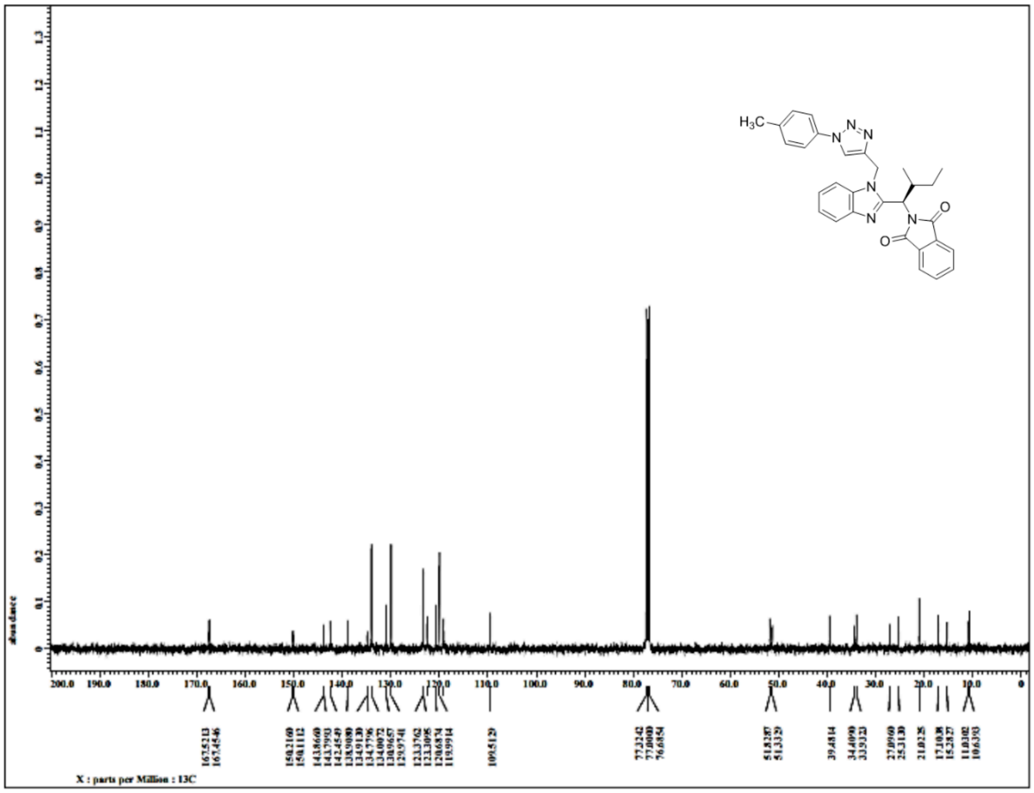

^{13}C NMR spectrum of compound **6j** ($CDCl_3$, 100MHz)

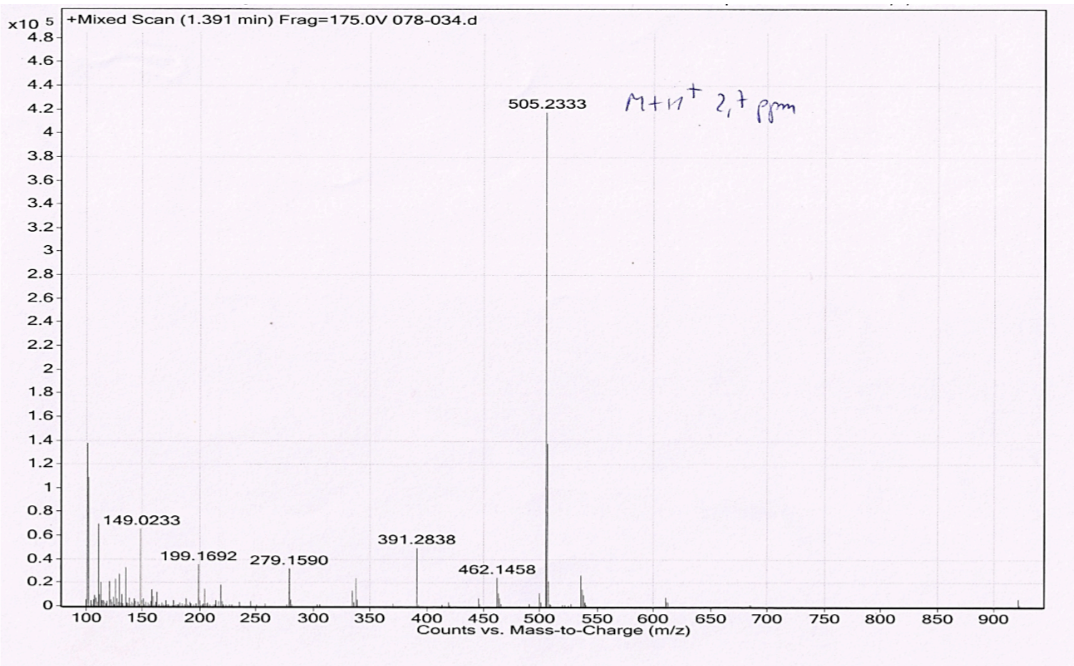

HRMS spectrum of compound **6j**

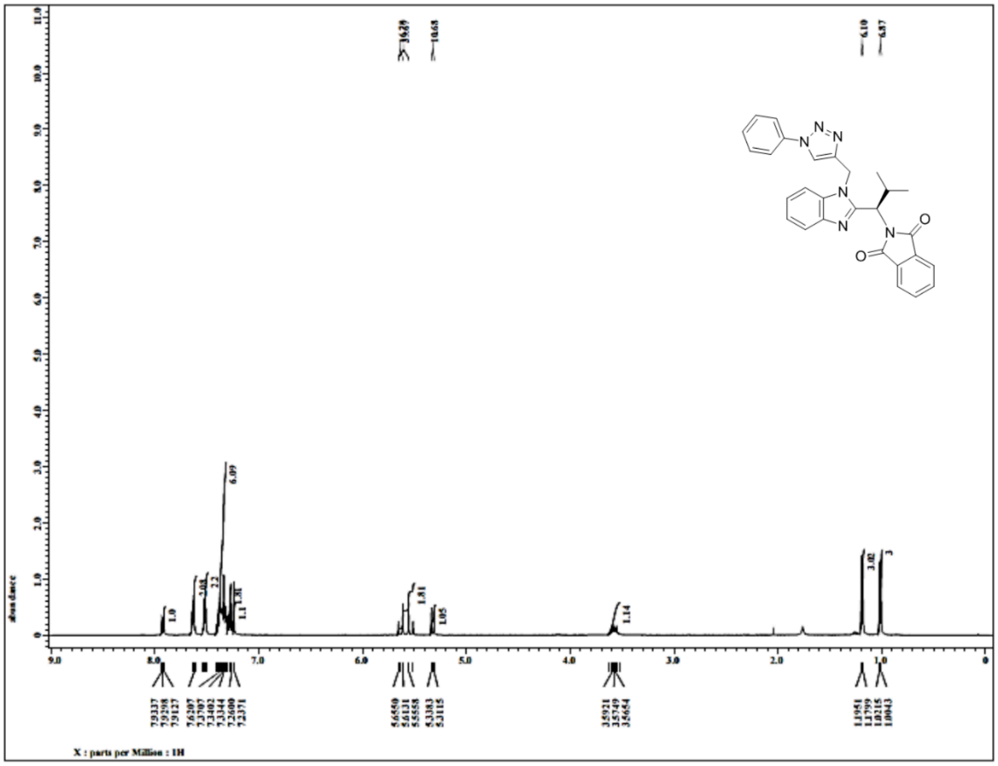

^{1}H NMR spectrum of compound **6k** ($CDCl_3$, 400MHz)

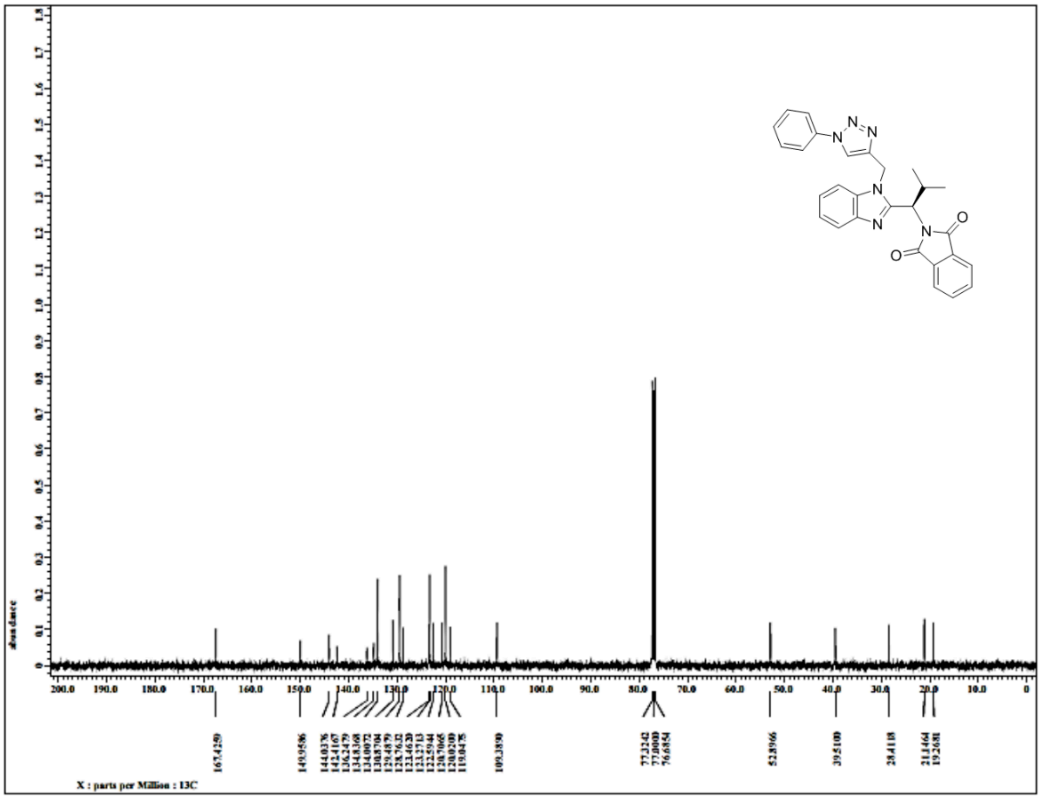

^{13}C NMR spectrum of compound **6k** ($CDCl_3$, 100MHz)

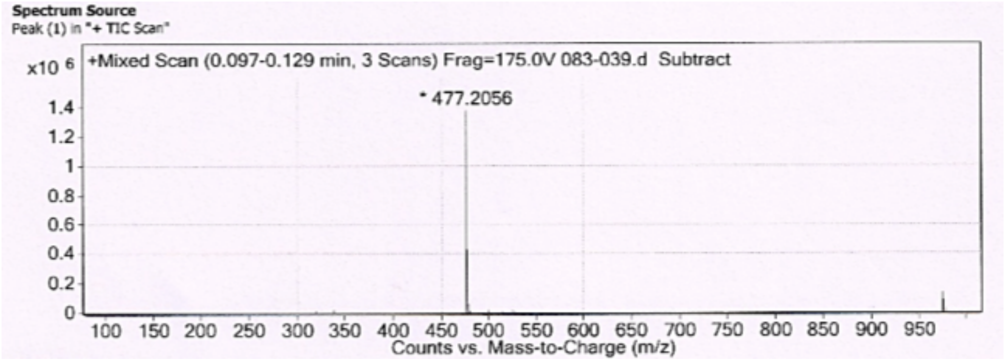

HRMS spectrum of compound **6k**

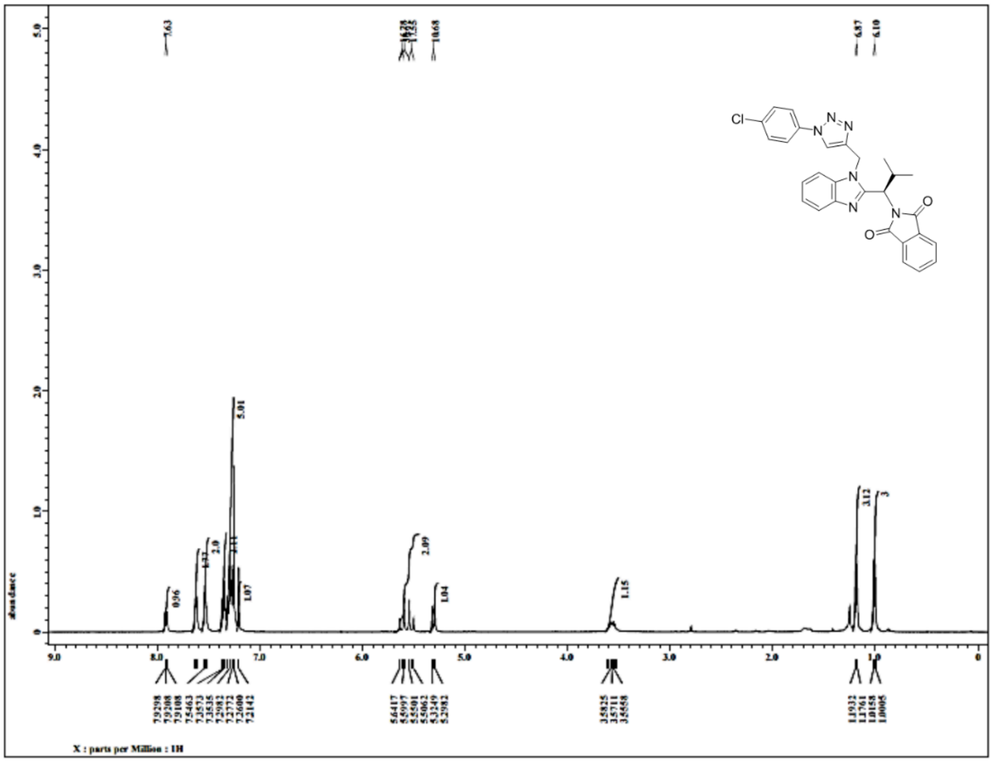

^{1}H NMR spectrum of compound **6l** ($CDCl_3$, 400MHz)

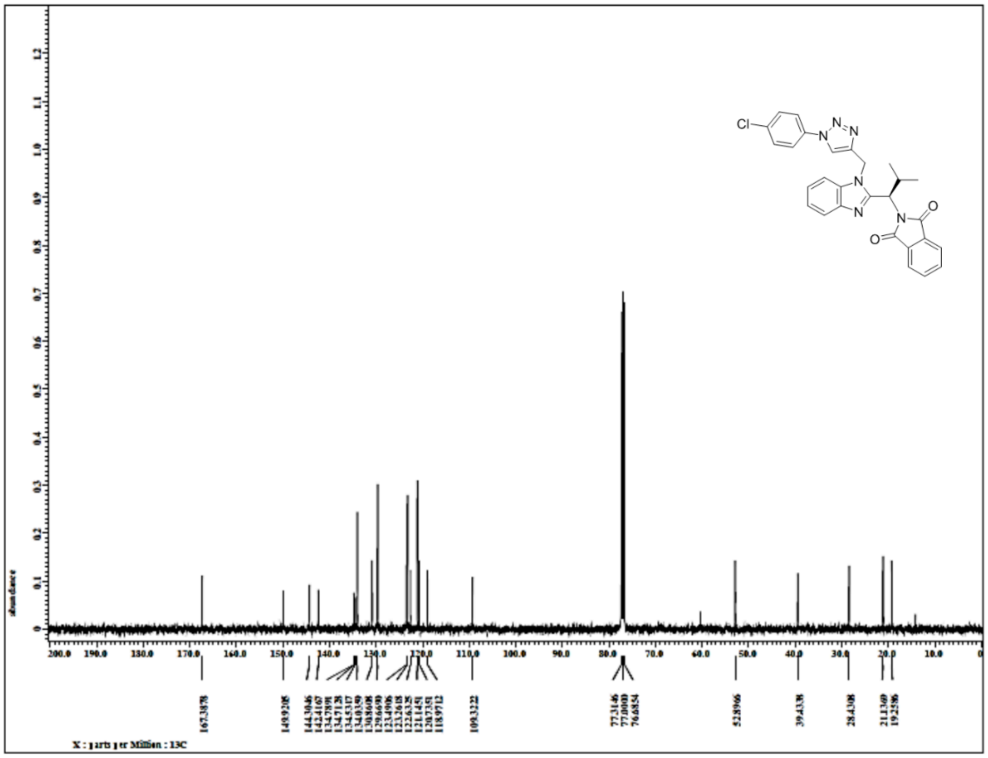

^{13}C NMR spectrum of compound **6l** ($CDCl_3$, 100MHz)

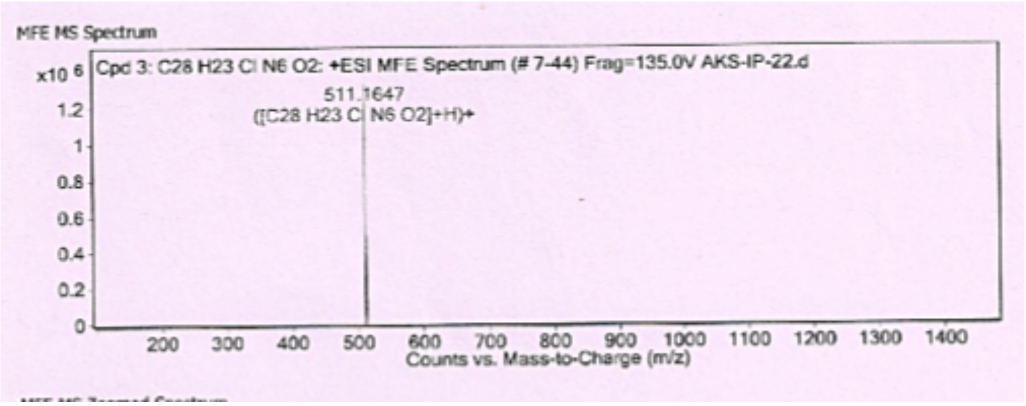

HRMS spectrum of compound **6l**

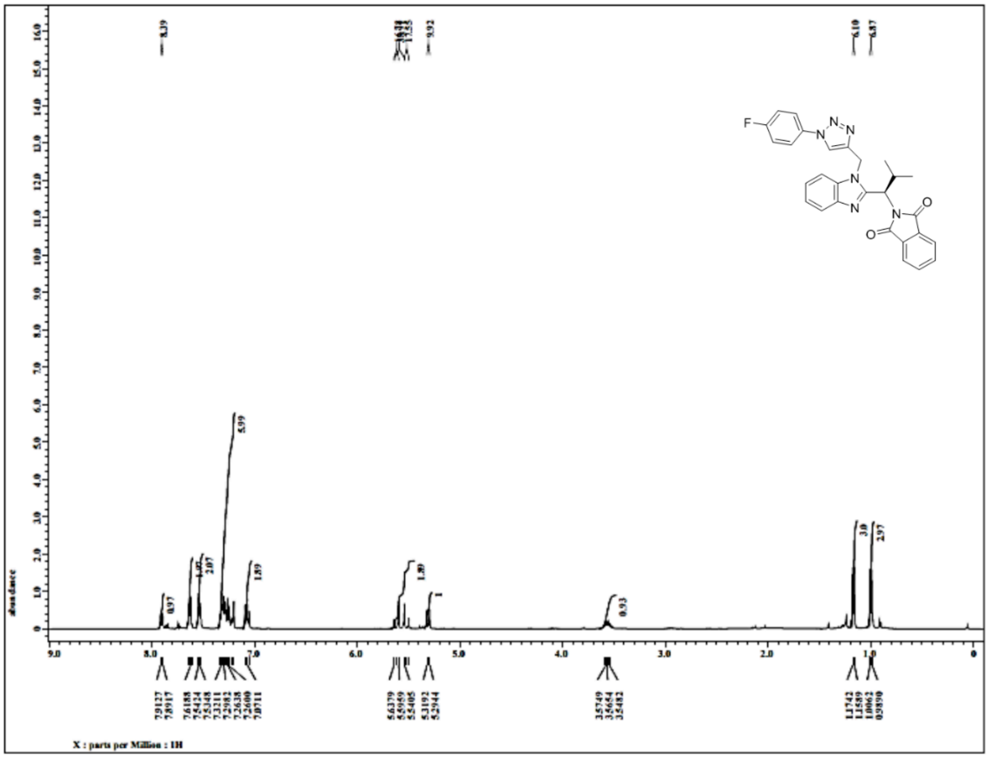

^{1}H NMR spectrum of compound **6m** ($CDCl_3$, 400MHz)

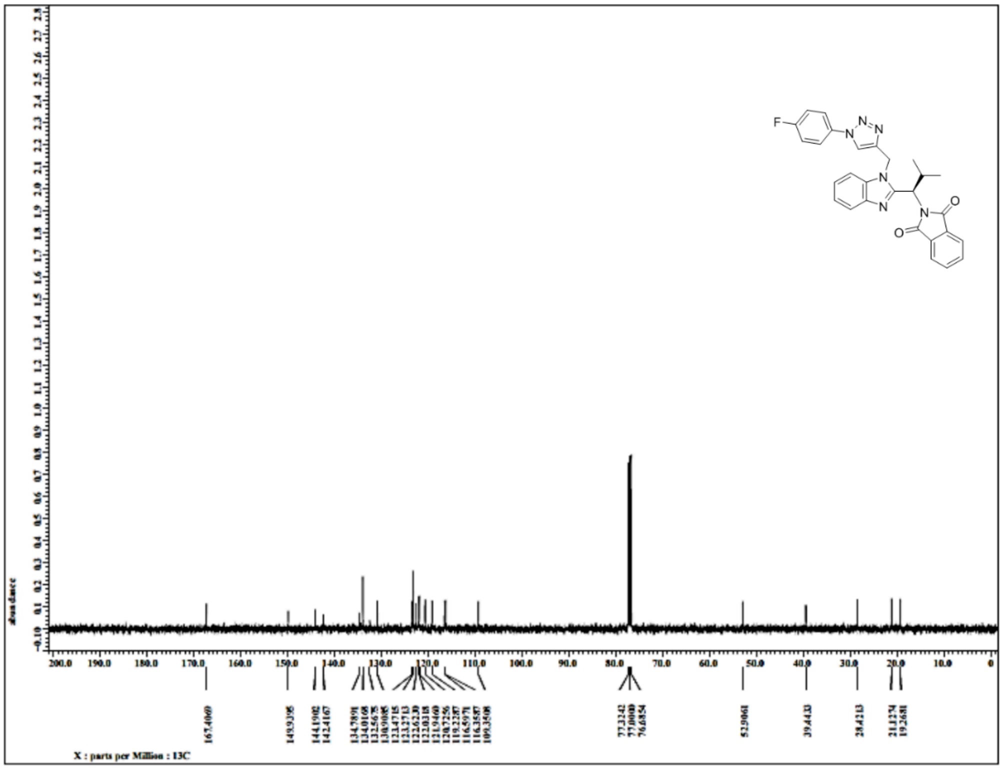

^{13}C NMR spectrum of compound **6m** ($CDCl_3$, 100MHz)

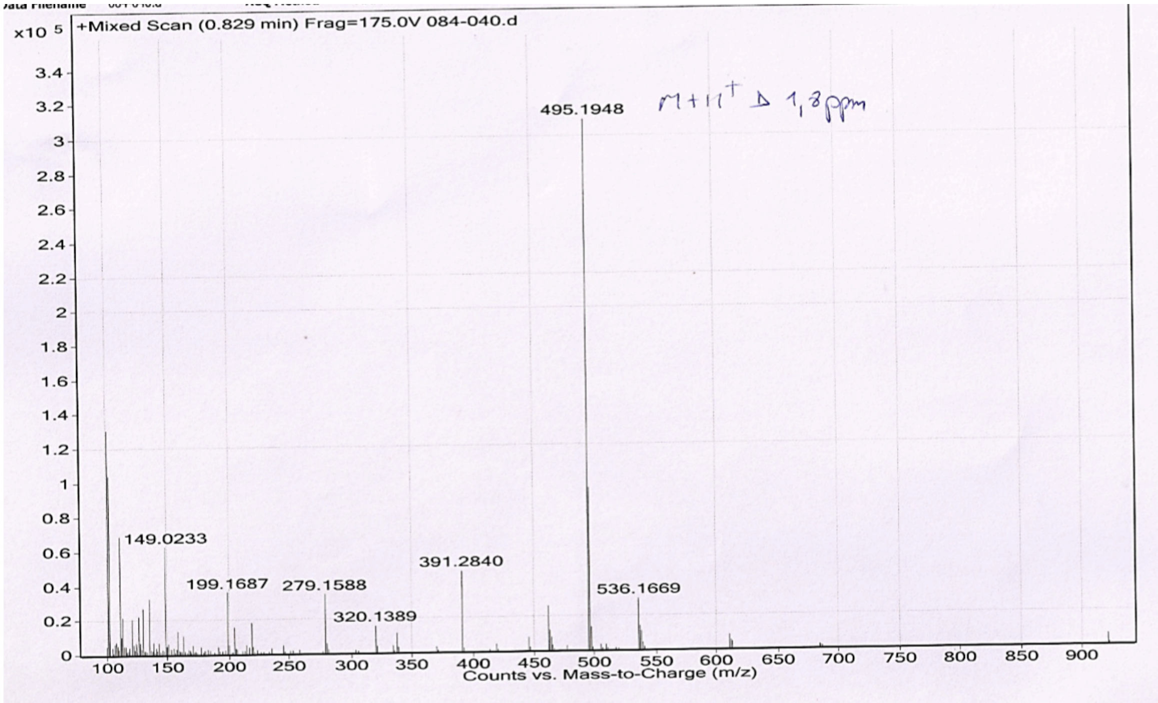

HRMS spectrum of compound **6m**

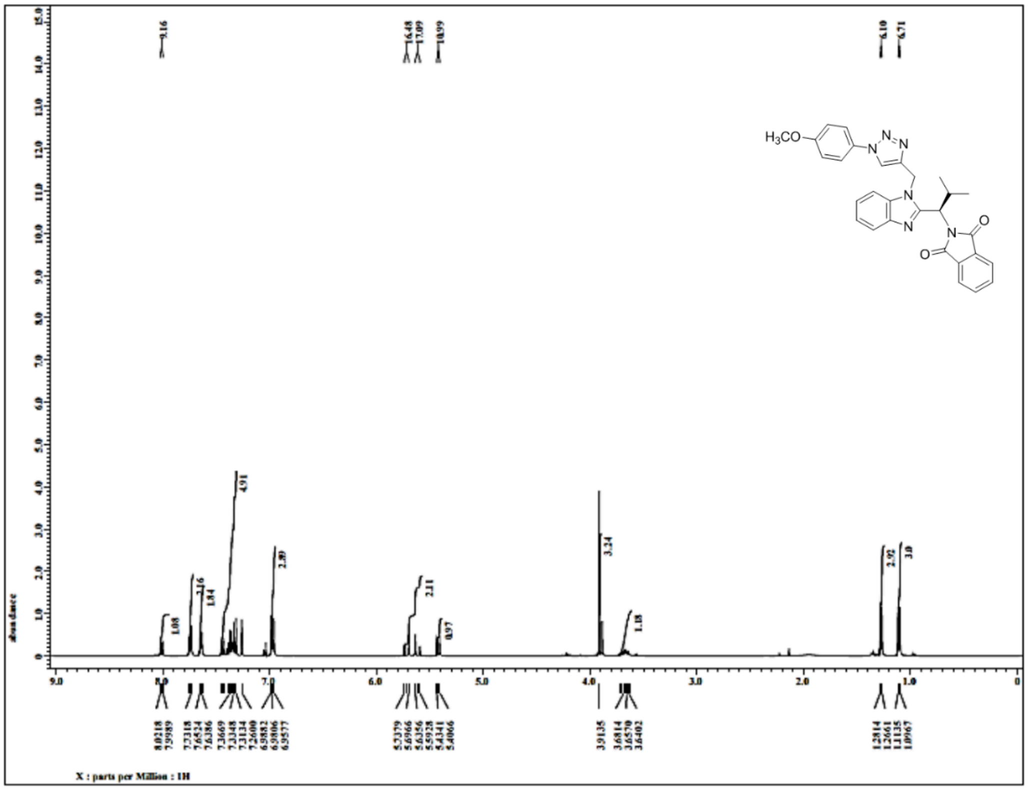

^{1}H NMR spectrum of compound **6n** ($CDCl_3$, 400MHz)

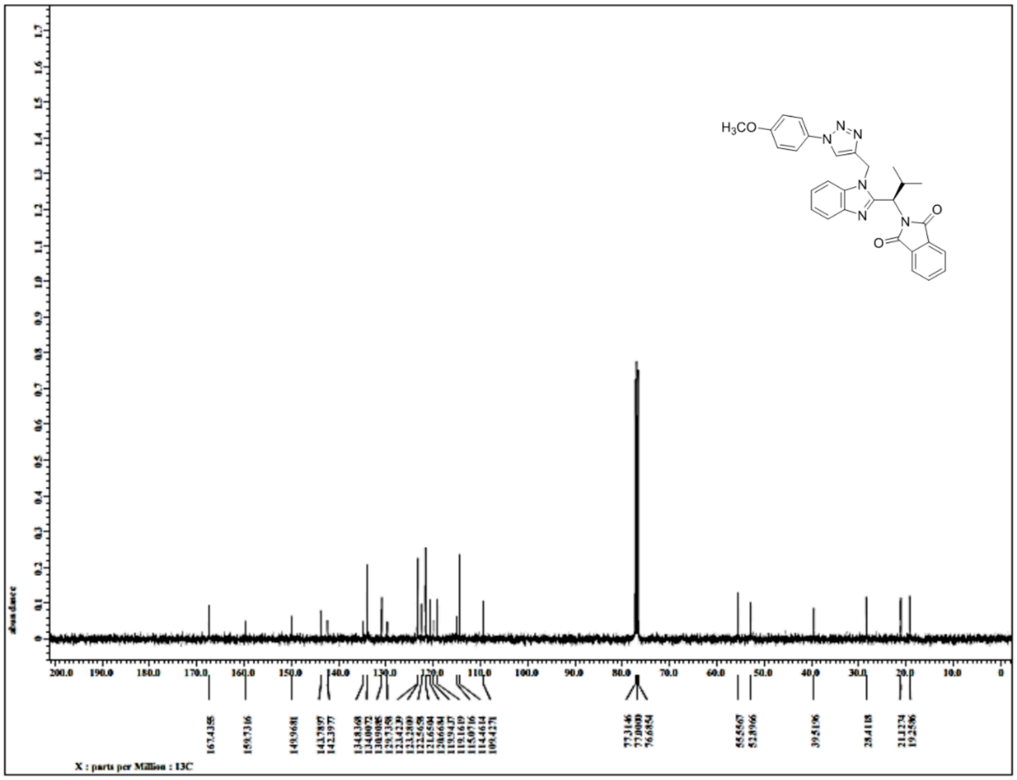

^{13}C NMR spectrum of compound **6n** ($CDCl_3$, 100MHz)

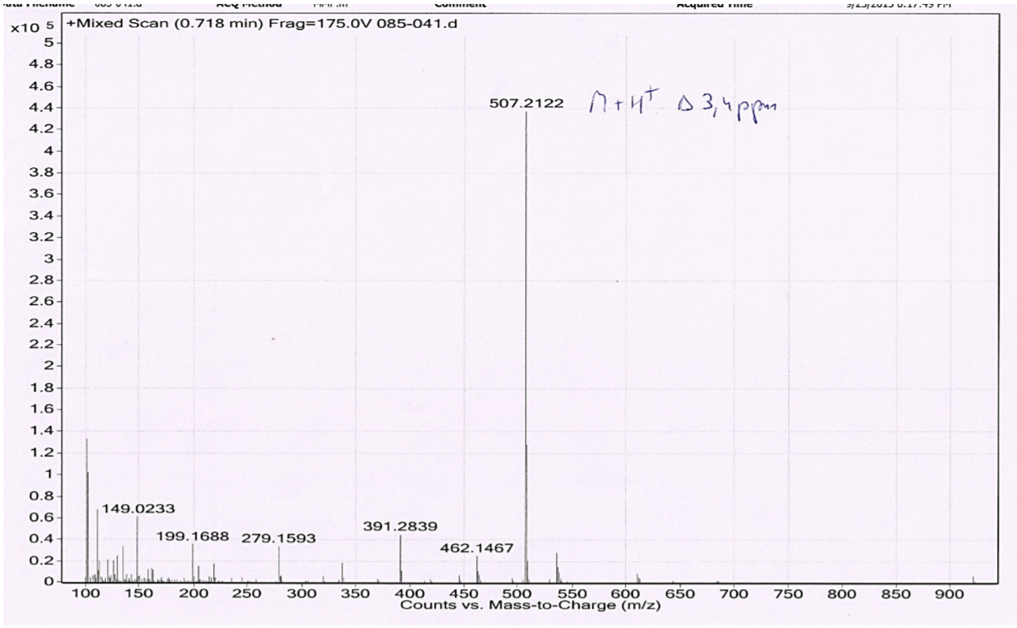

HRMS spectrum of compound **6n**

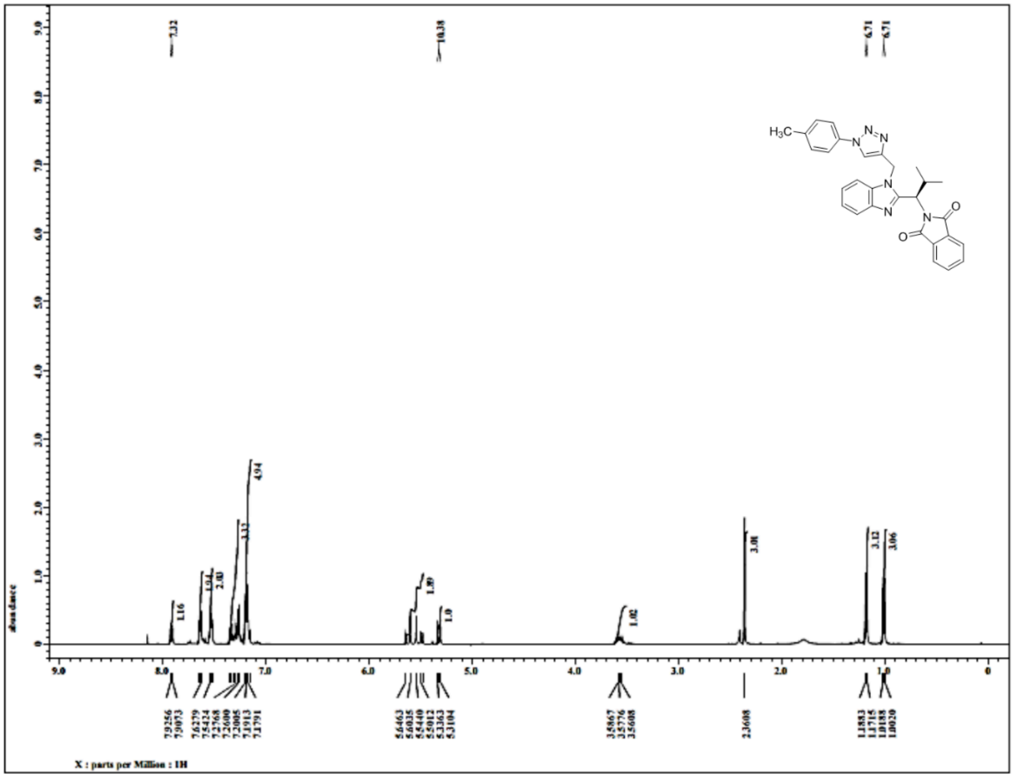

^{1}H NMR spectrum of compound **6o** ($CDCl_3$, 400MHz)

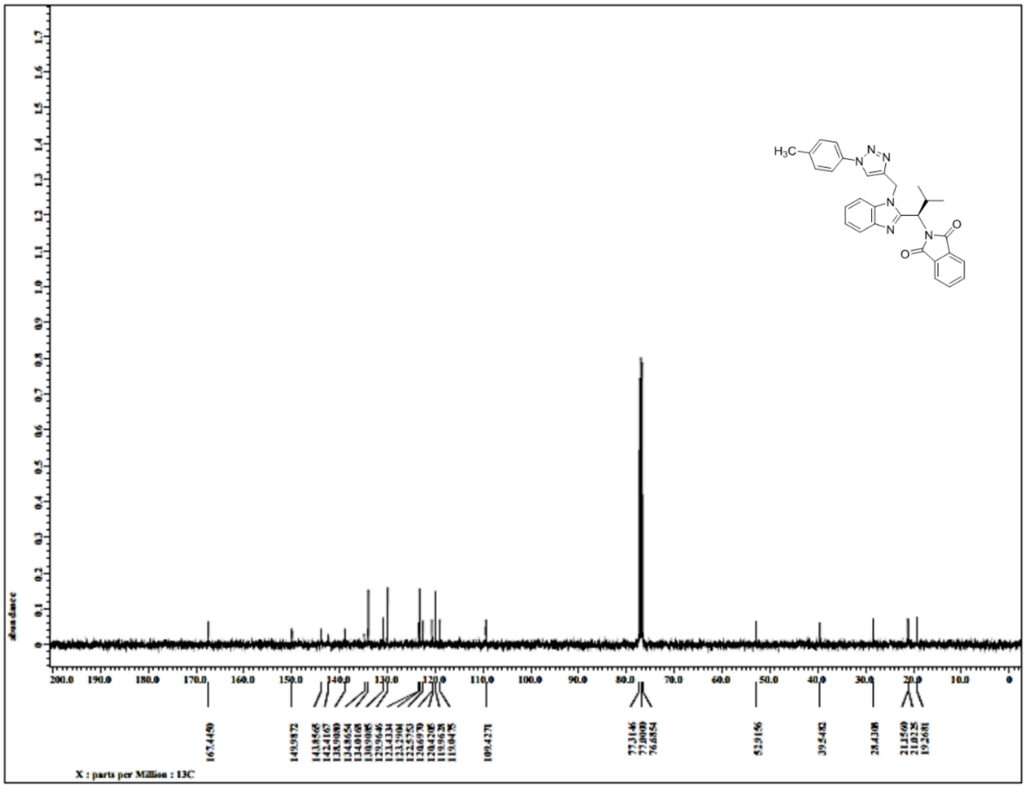

^{13}C NMR spectrum of compound **6o** ($CDCl_3$, 100MHz)

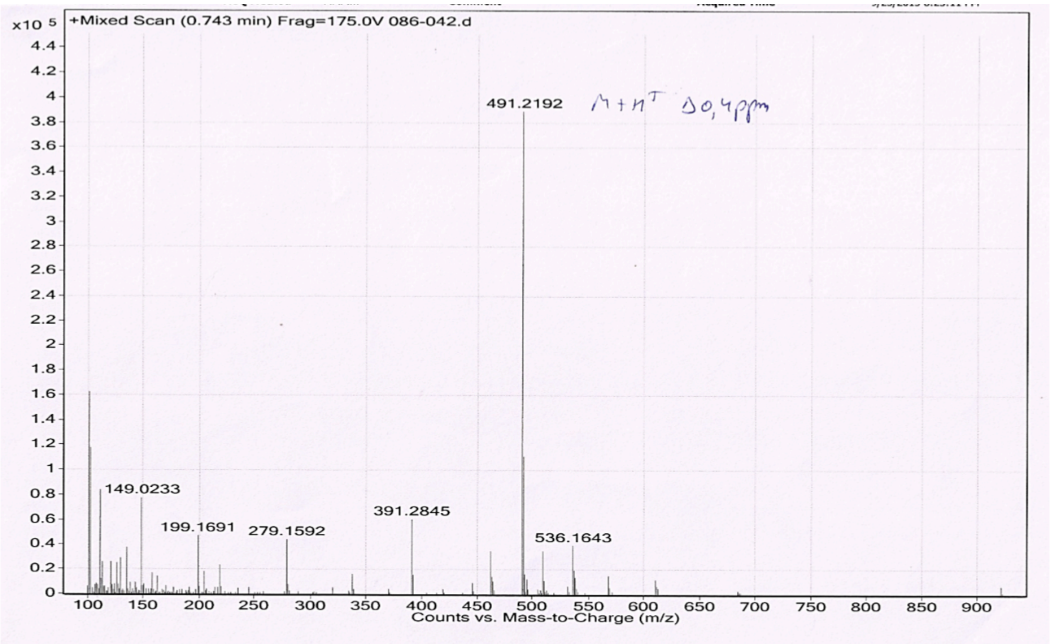

HRMS spectrum of compound **6o**

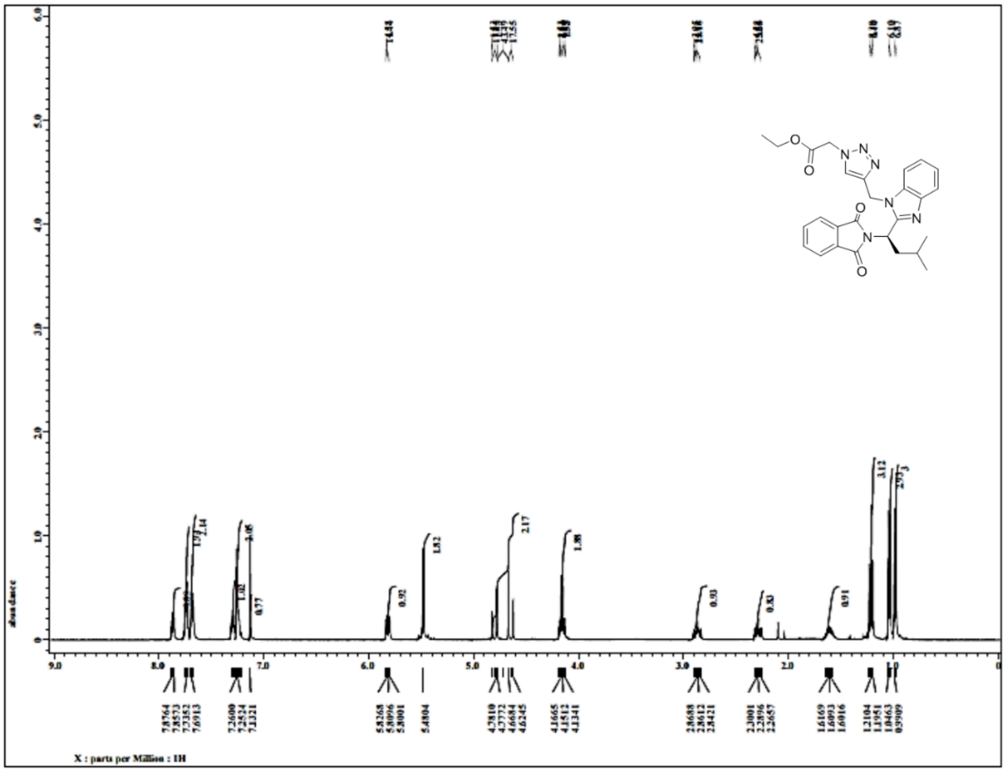

^{1}H NMR spectrum of compound **6p** ($CDCl_3$, 400MHz)

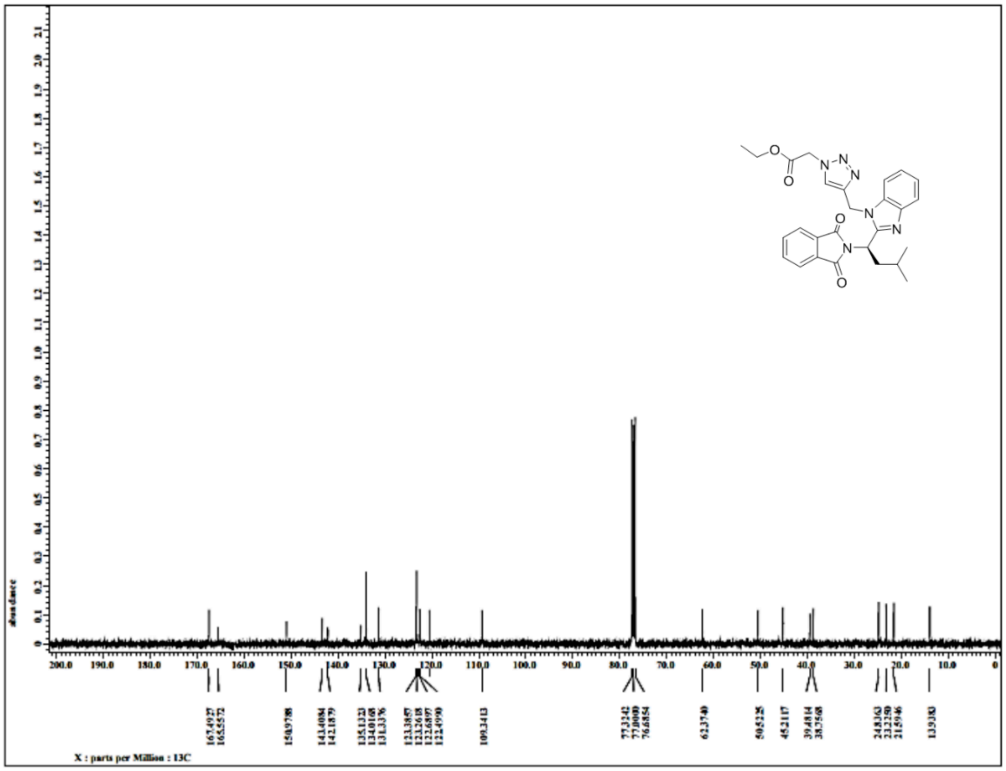

^{13}C NMR spectrum of compound **6p** ($CDCl_3$, 100MHz)

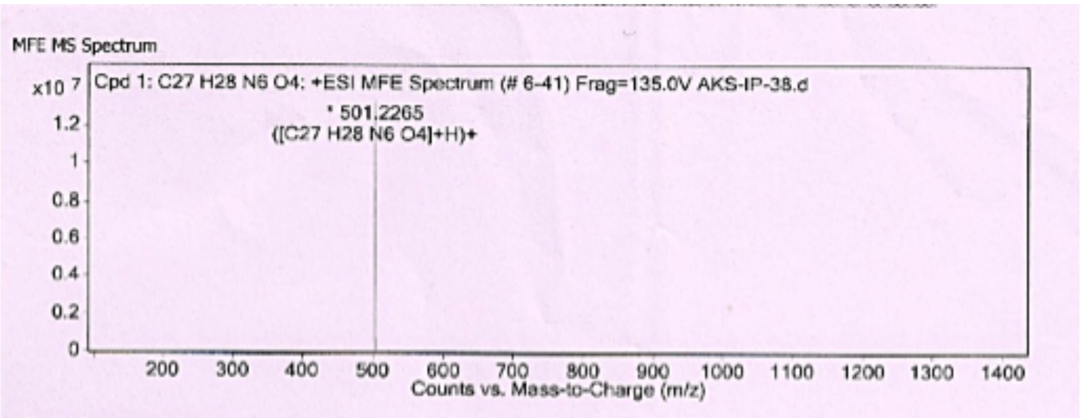

HRMS spectrum of compound **6p**

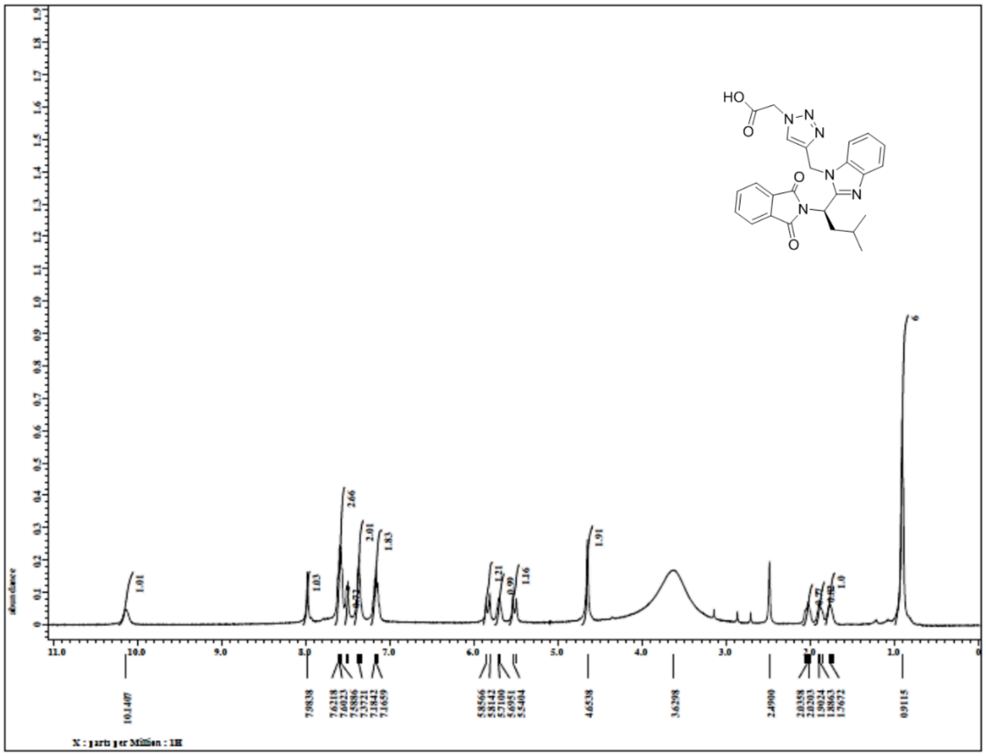

^{1}H NMR spectrum of compound **6q** (DMSO, 400MHz)

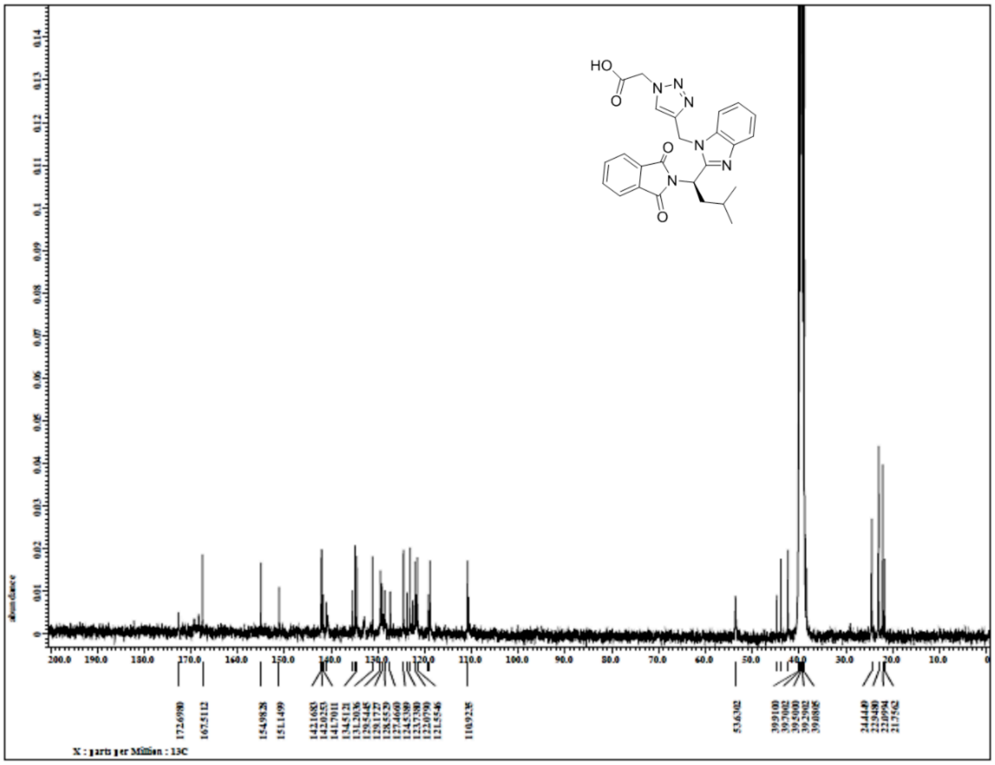

^{13}C NMR spectrum of compound **6q** (DMSO, 100MHz)

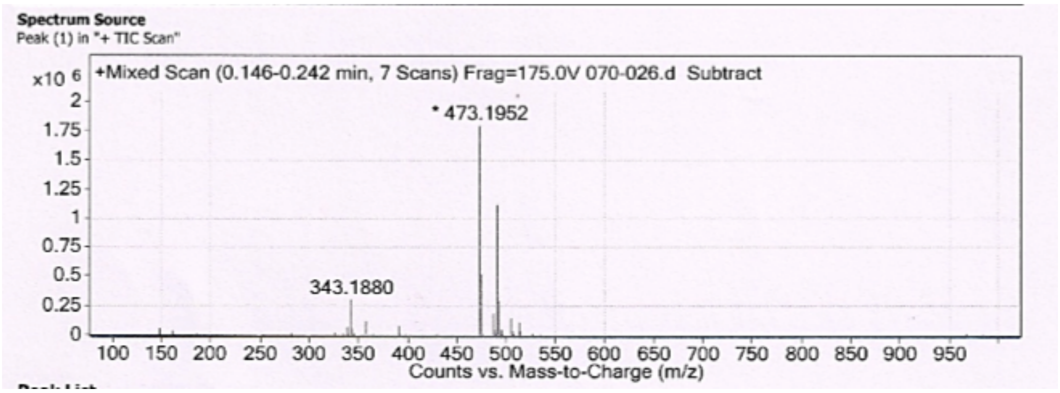

HRMS spectrum of compound **6q**

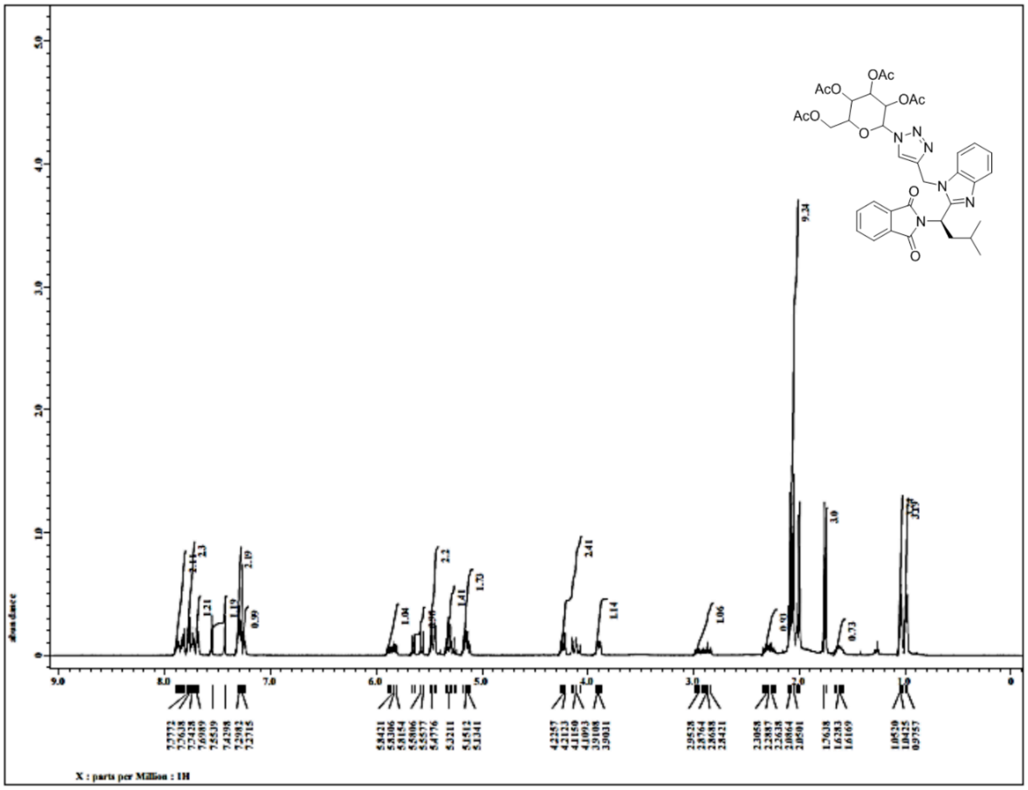

^{1}H NMR spectrum of compound **6r** ($CDCl_3$, 400MHz)

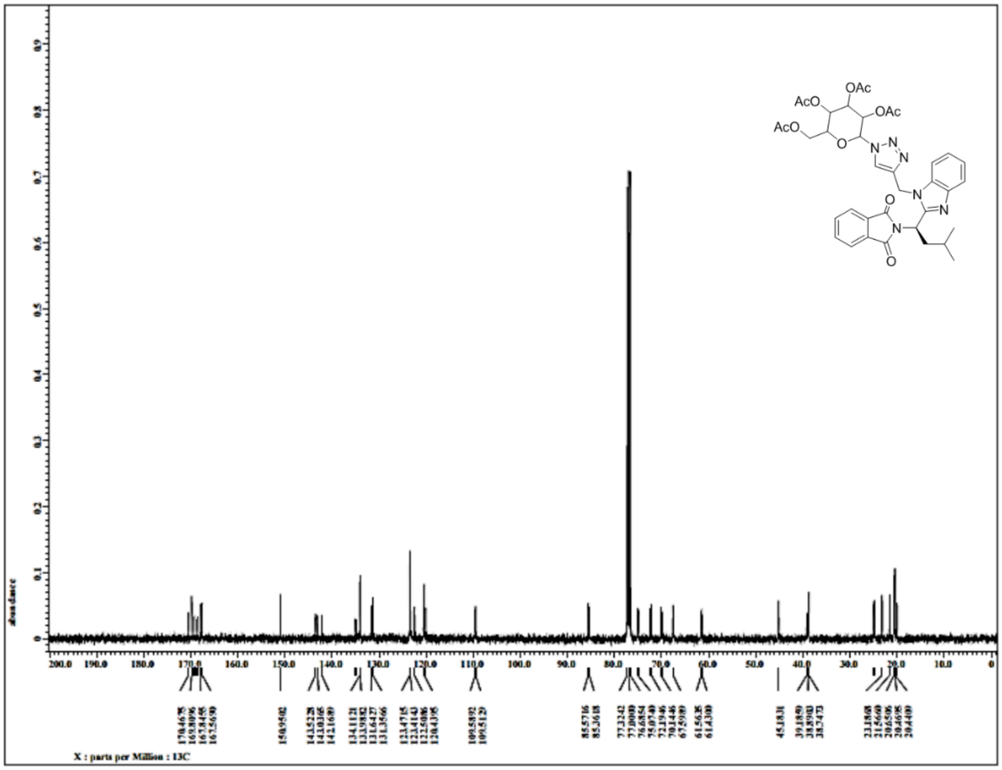

^{13}C NMR spectrum of compound **6r** ($CDCl_3$, 100MHz)

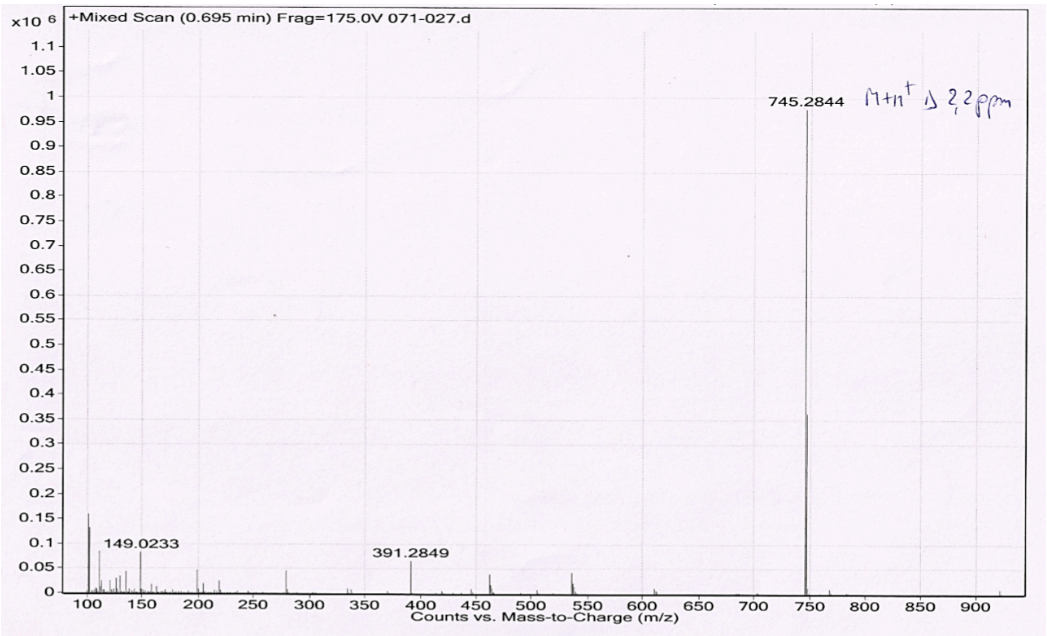

HRMS spectrum of compound **6r**

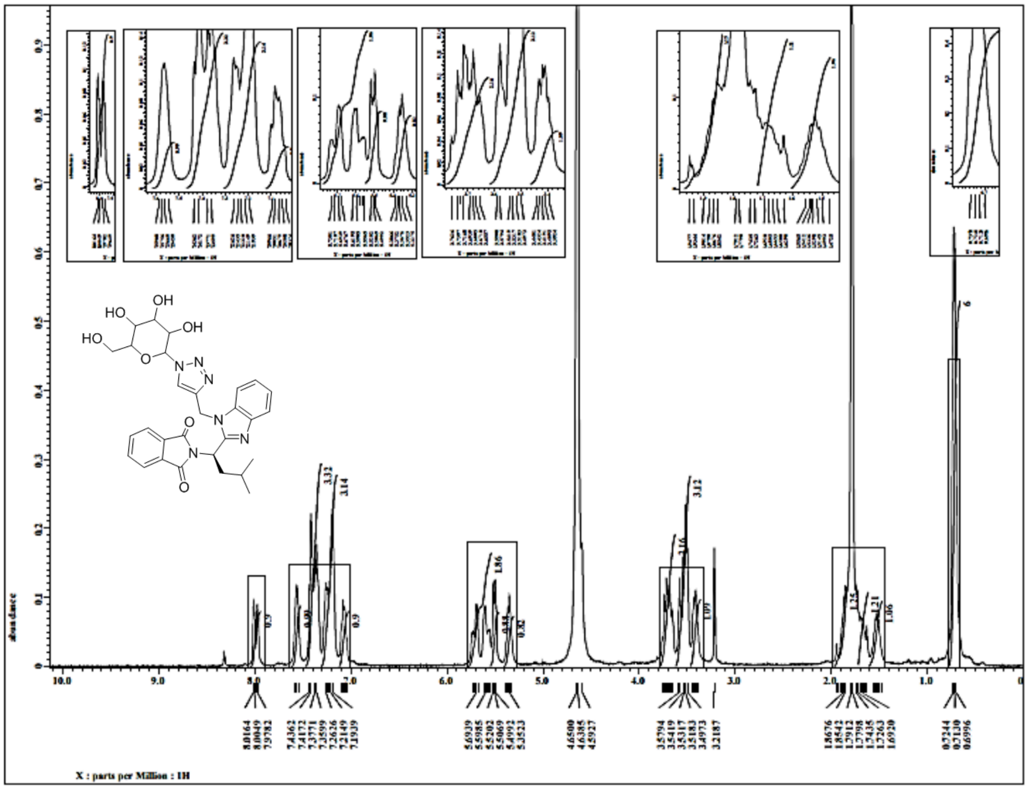

^{1}H NMR spectrum of compound **6s** (D_2O, 400MHz)

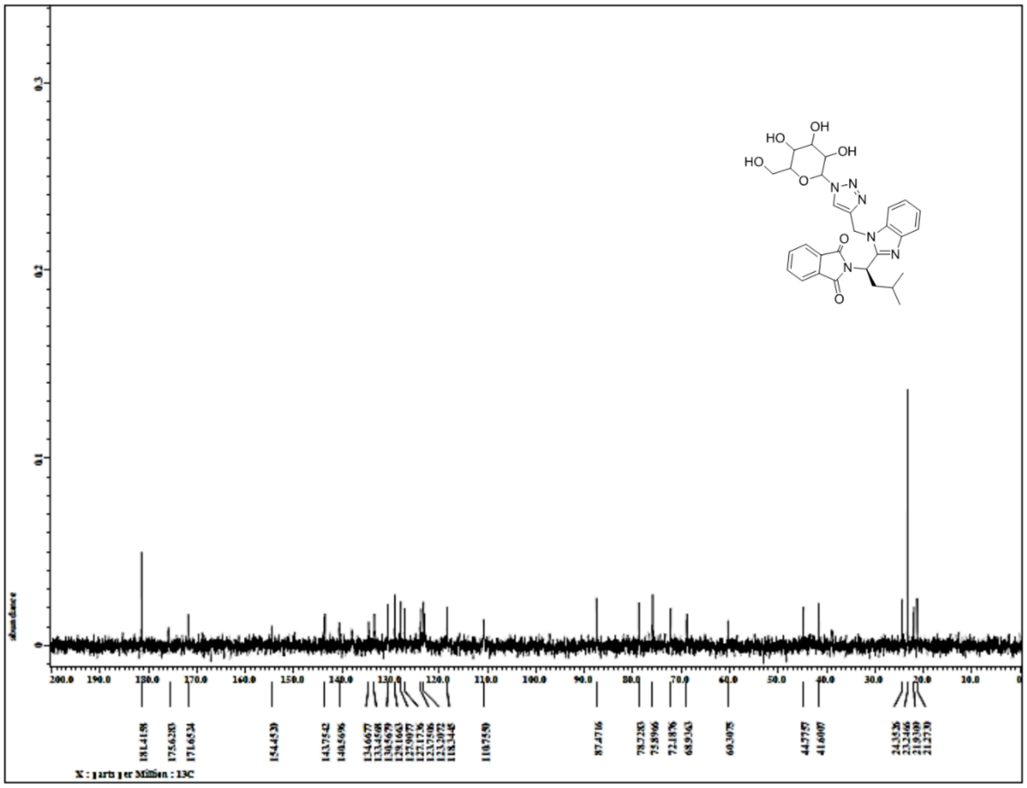

^{13}C NMR spectrum of compound **6s** (D_2O, 100MHz)

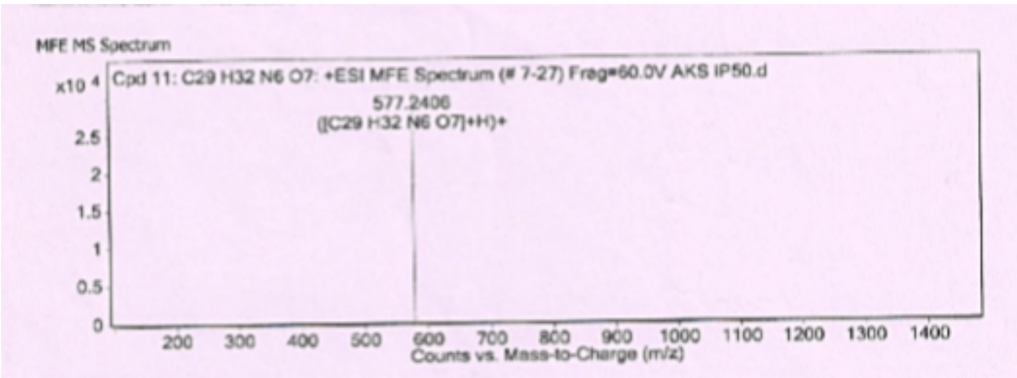

HRMS spectrum of compound **6s**

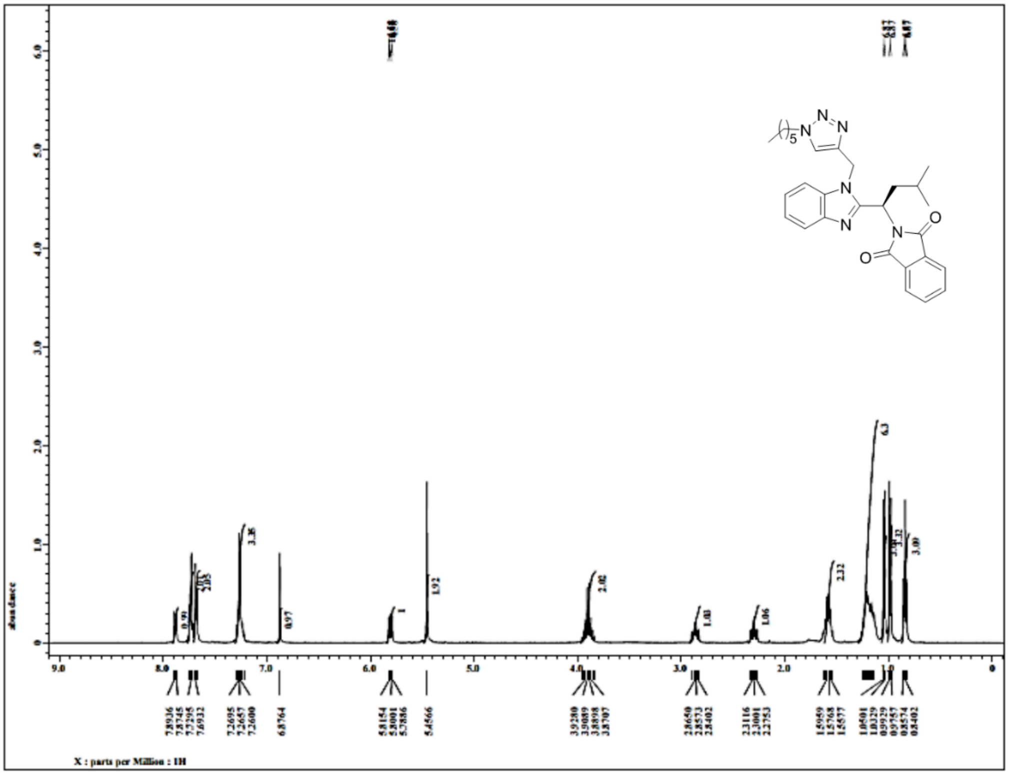

^{1}H NMR spectrum of compound **6t** ($CDCl_3$, 400MHz)

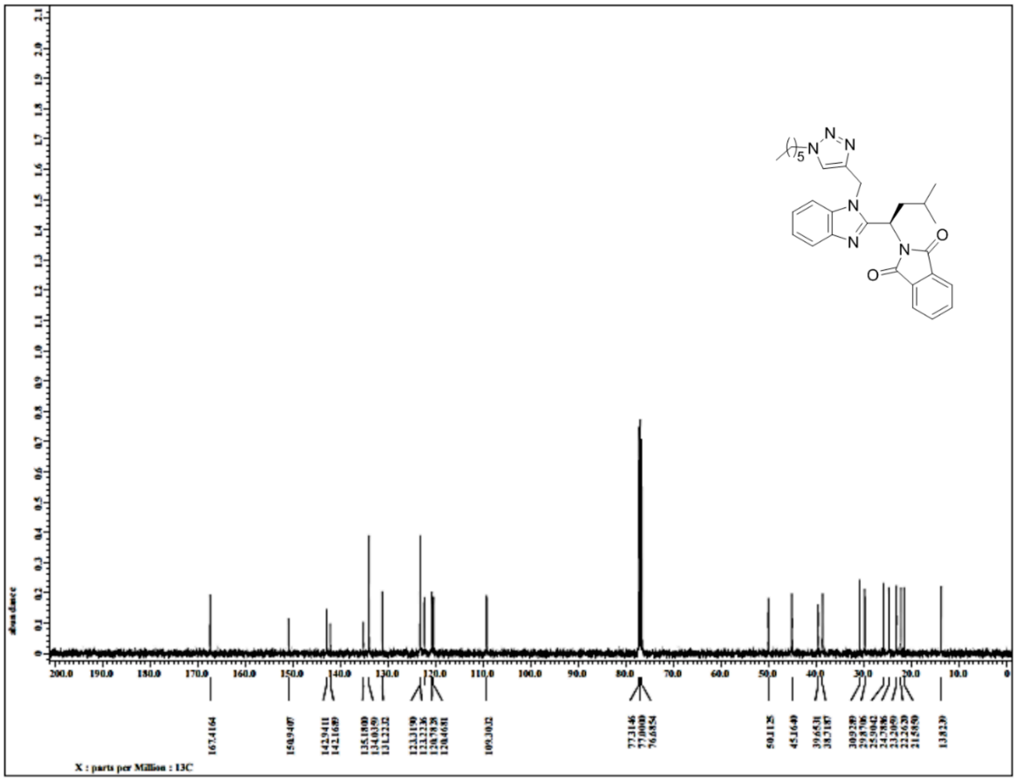

^{13}C NMR spectrum of compound **6t** ($CDCl_3$, 100MHz)

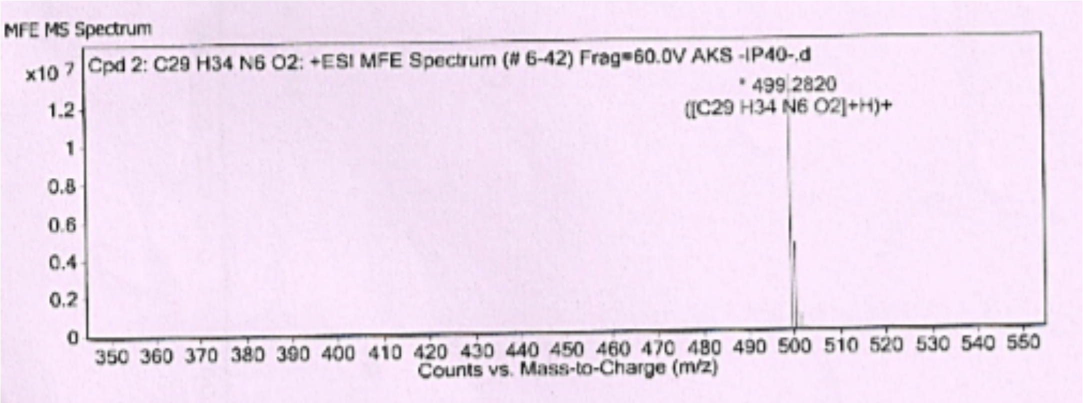

HRMS spectrum of compound **6t**

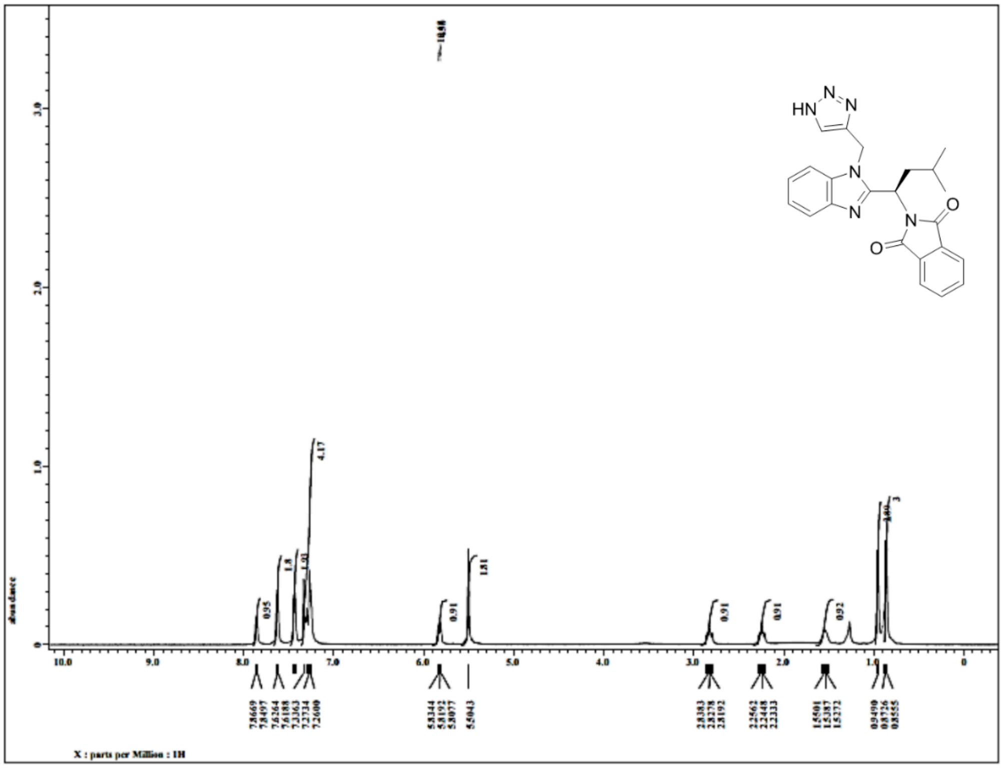

^{1}H NMR spectrum of compound **6u** ($CDCl_3$, 400MHz)

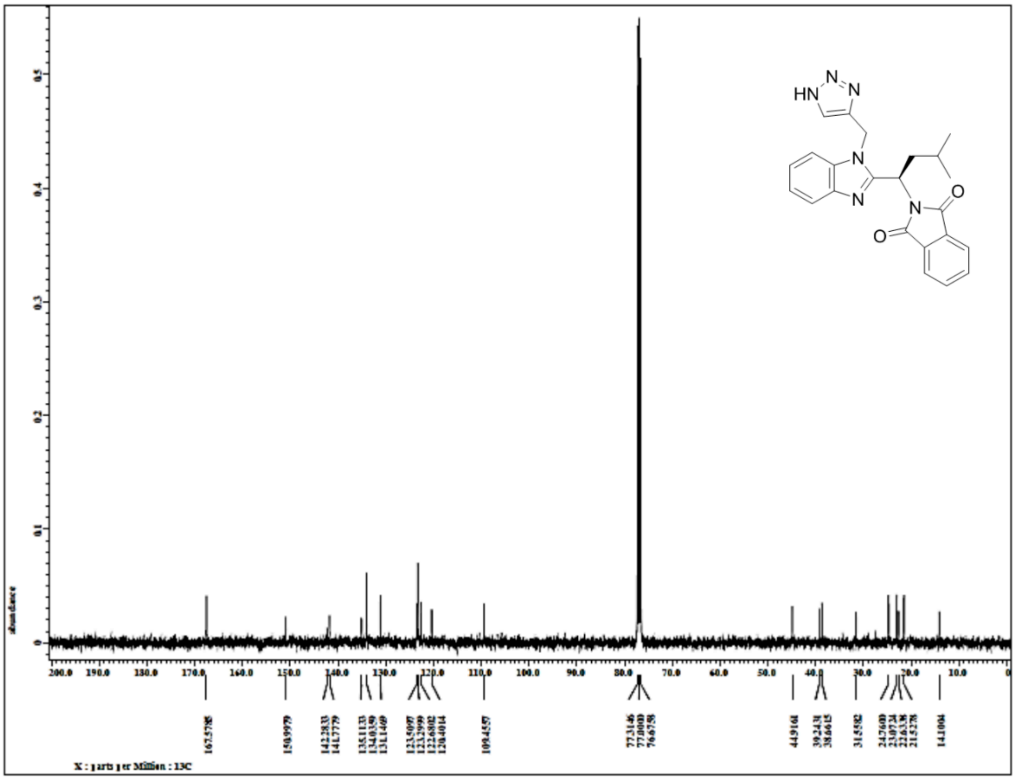

^{13}C NMR spectrum of compound **6u** ($CDCl_3$, 100MHz)

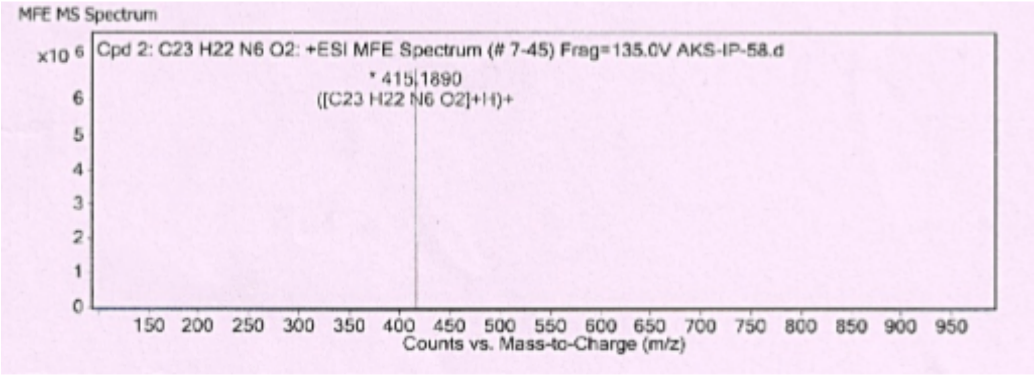

HRMS spectrum of compound **6u**

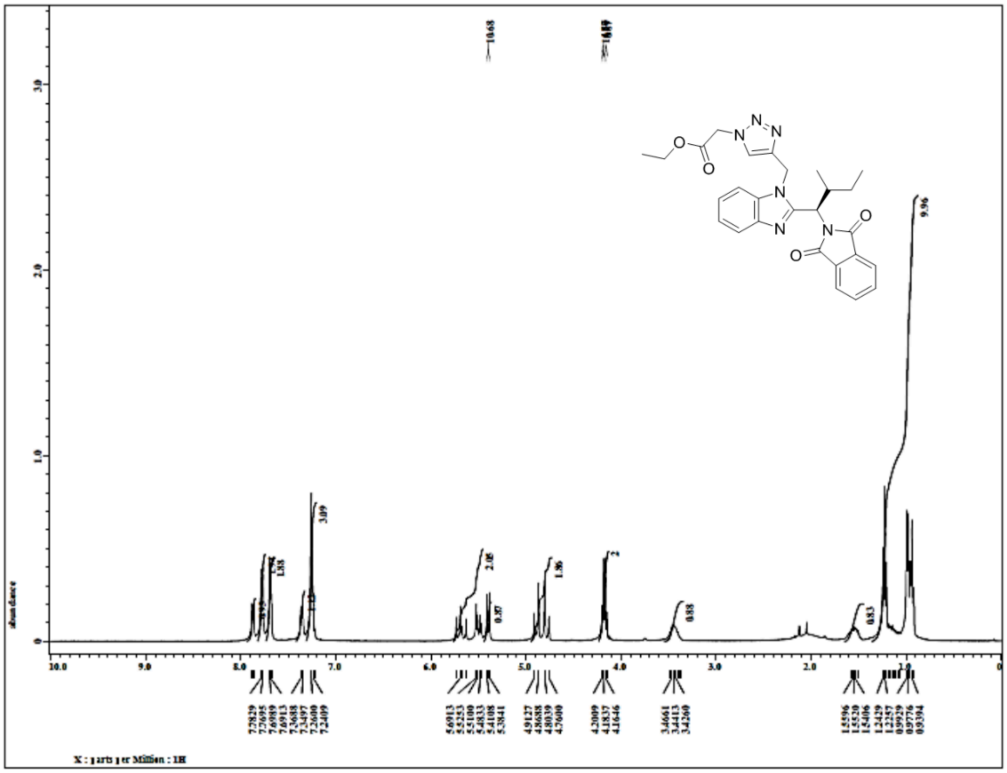

^{1}H NMR spectrum of compound **6v** ($CDCl_3$, 400MHz)

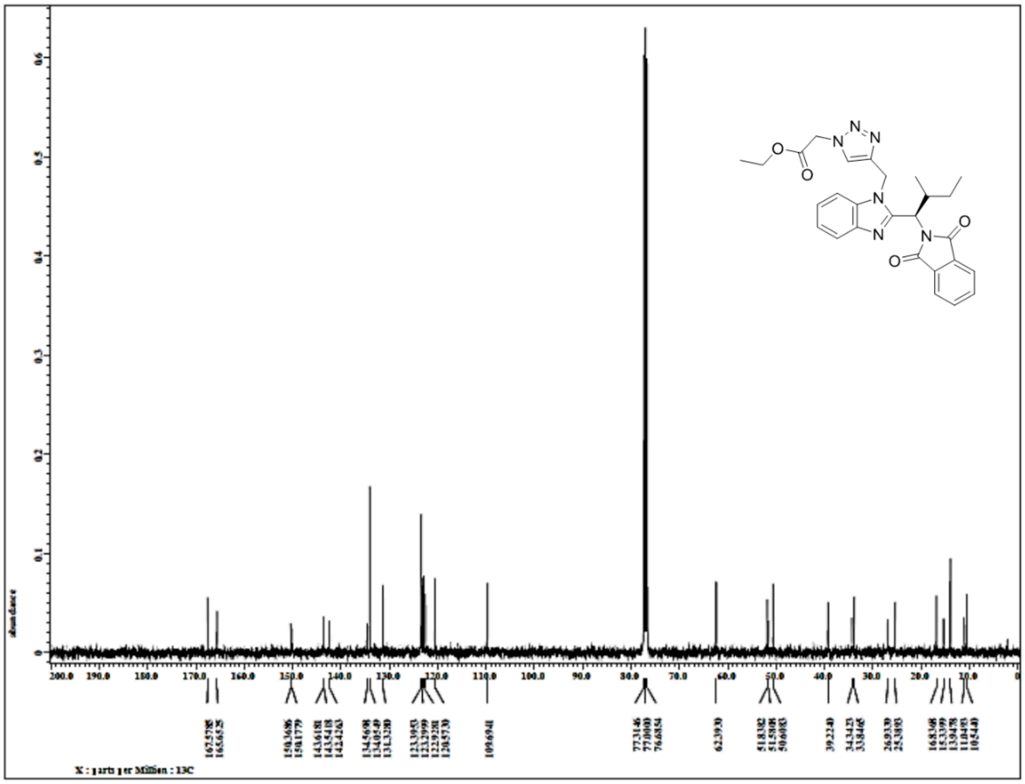

^{13}C NMR spectrum of compound **6v** ($CDCl_3$, 100MHz)

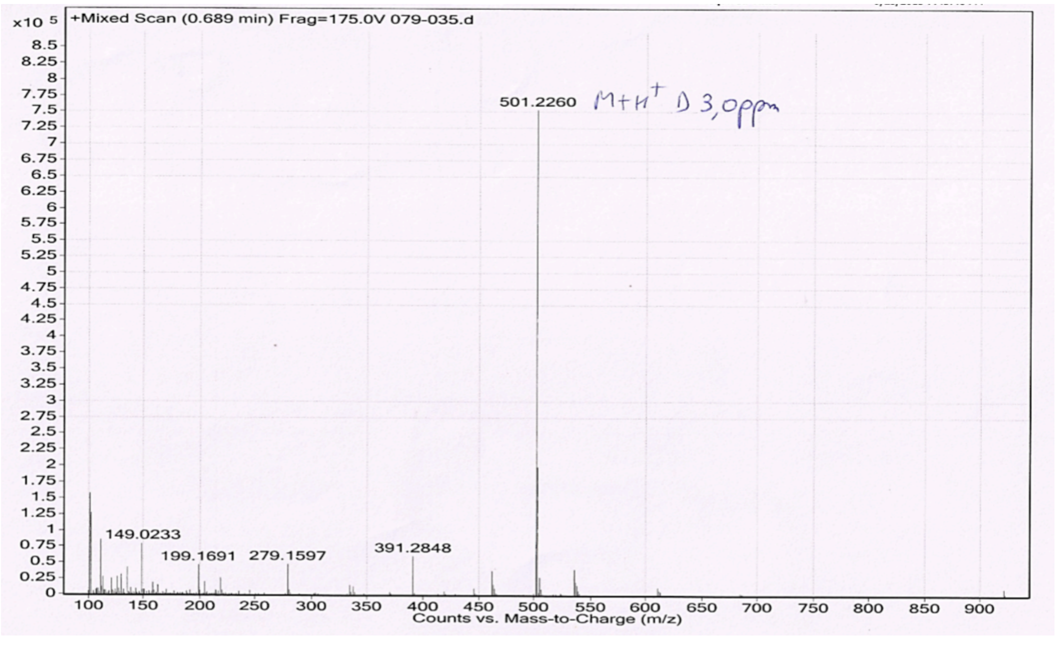

HRMS spectrum of compound **6v**

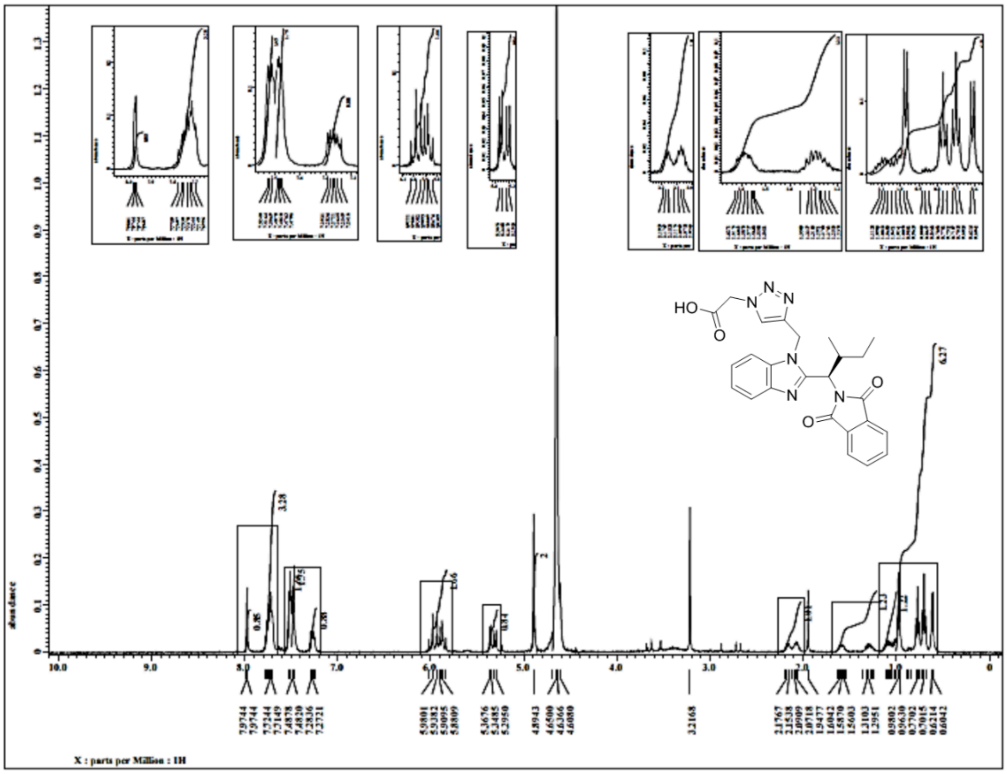

^{1}H NMR spectrum of compound **6w** (D_2O, 400MHz)

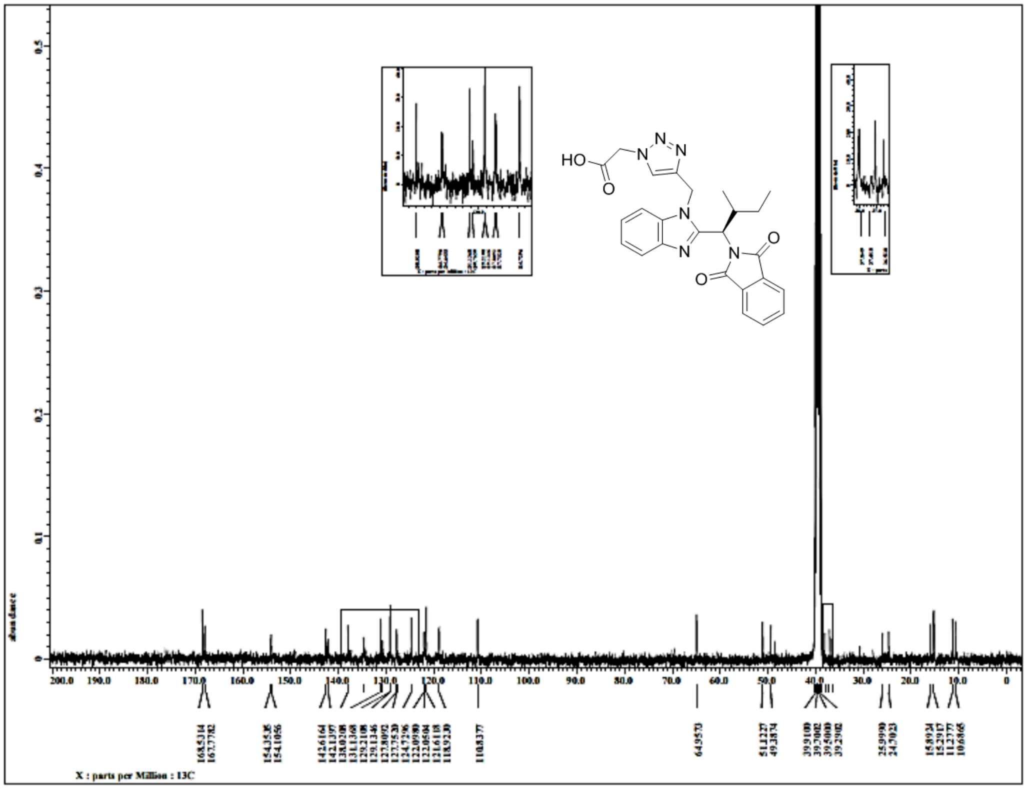

^{13}C NMR spectrum of compound **6w** (DMSO, 100MHz)

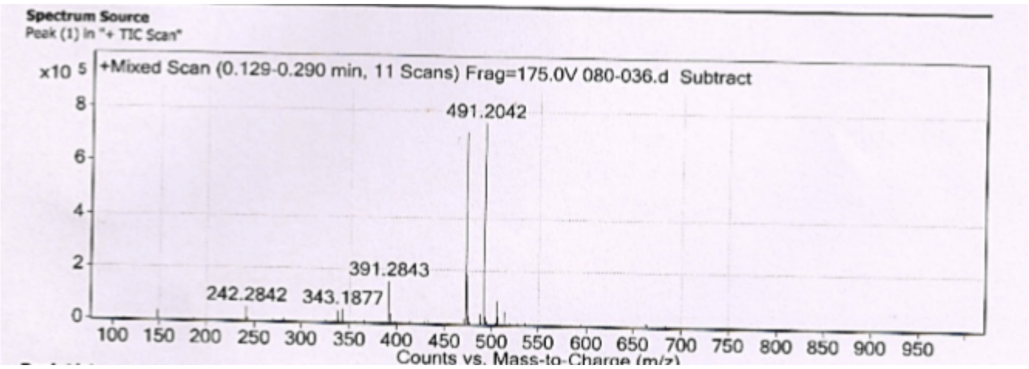

HRMS spectrum of compound **6w**

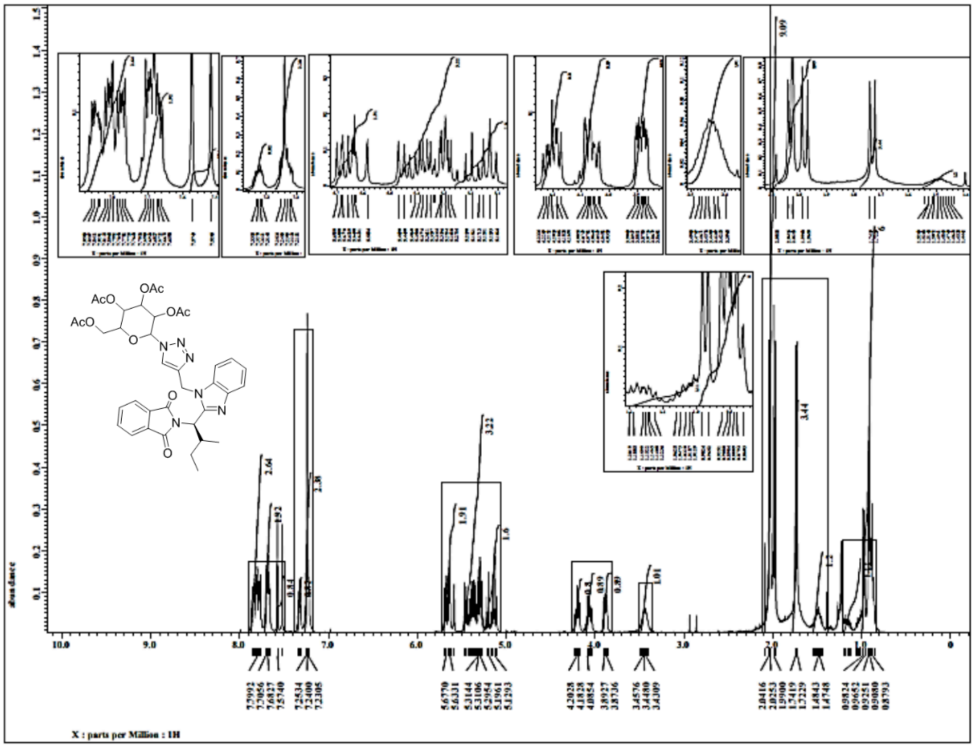

^{1}H NMR spectrum of compound **6x** ($CDCl_3$, 400MHz)

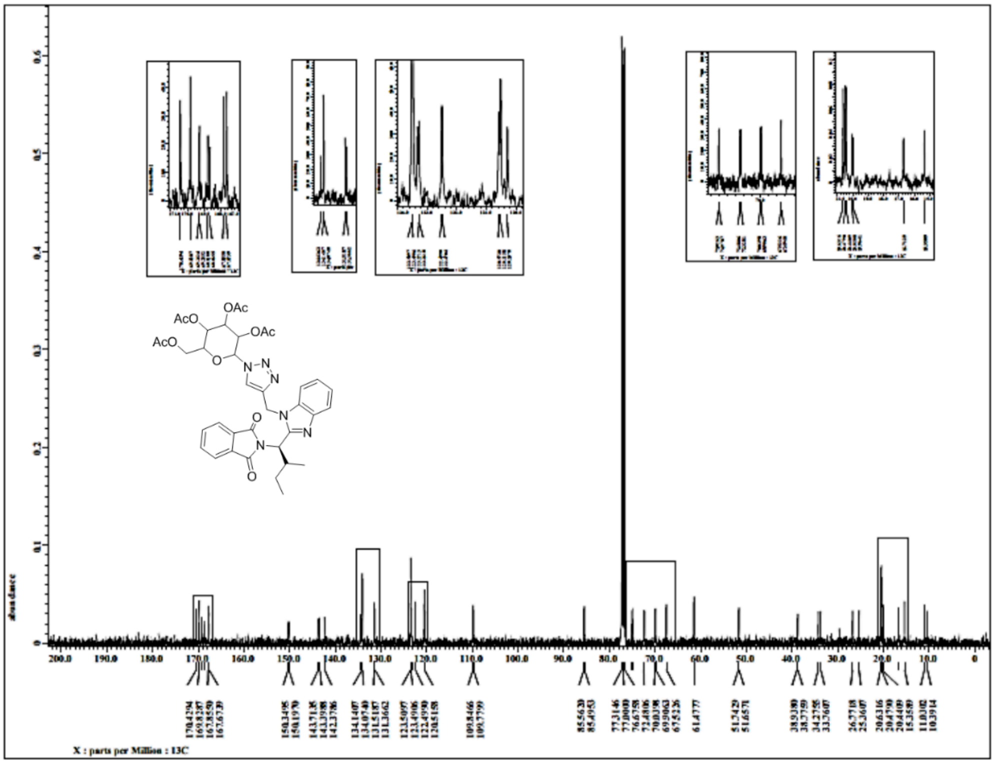

^{13}C NMR spectrum of compound **6x** ($CDCl_3$, 100MHz)

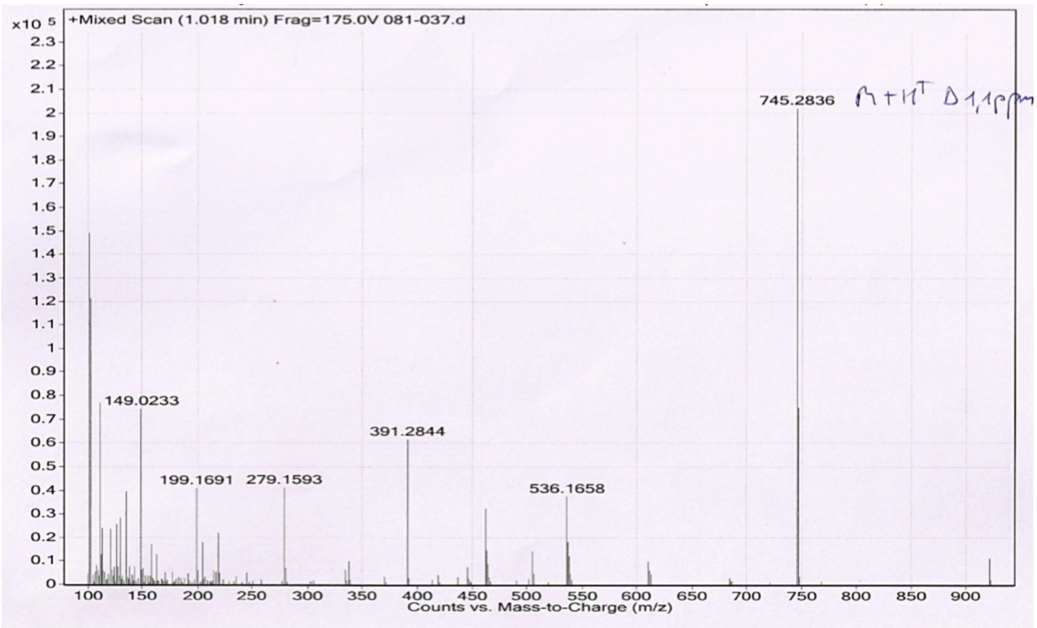

HRMS spectrum of compound **6x**

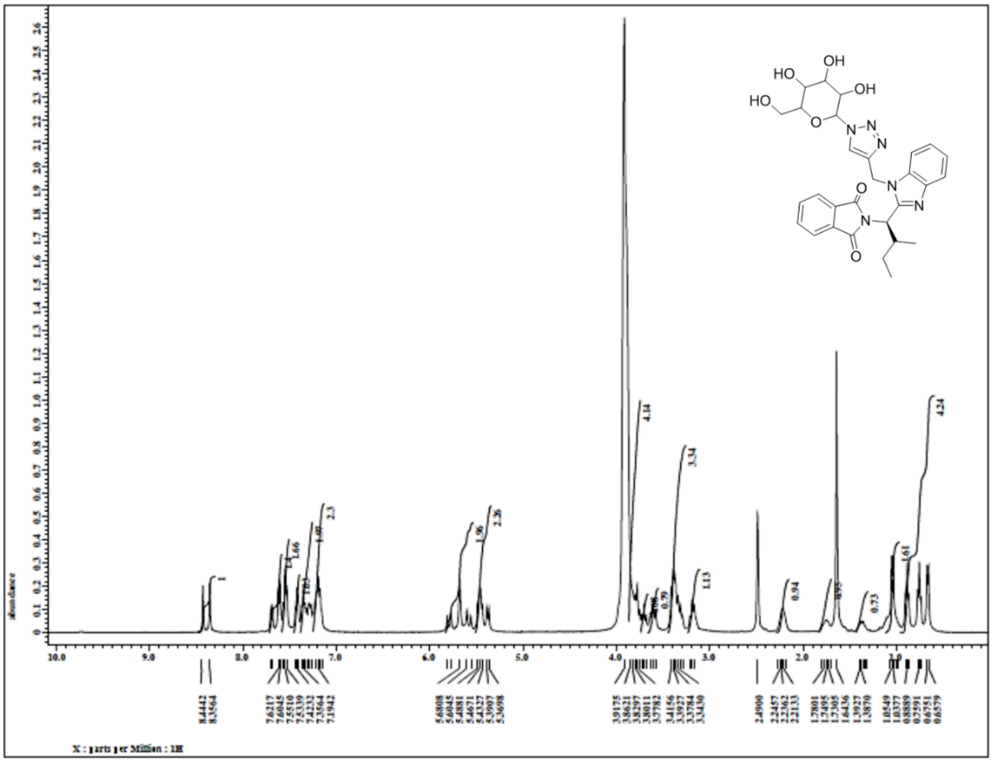

^{1}H NMR spectrum of compound **6y** (DMSO, 400MHz)

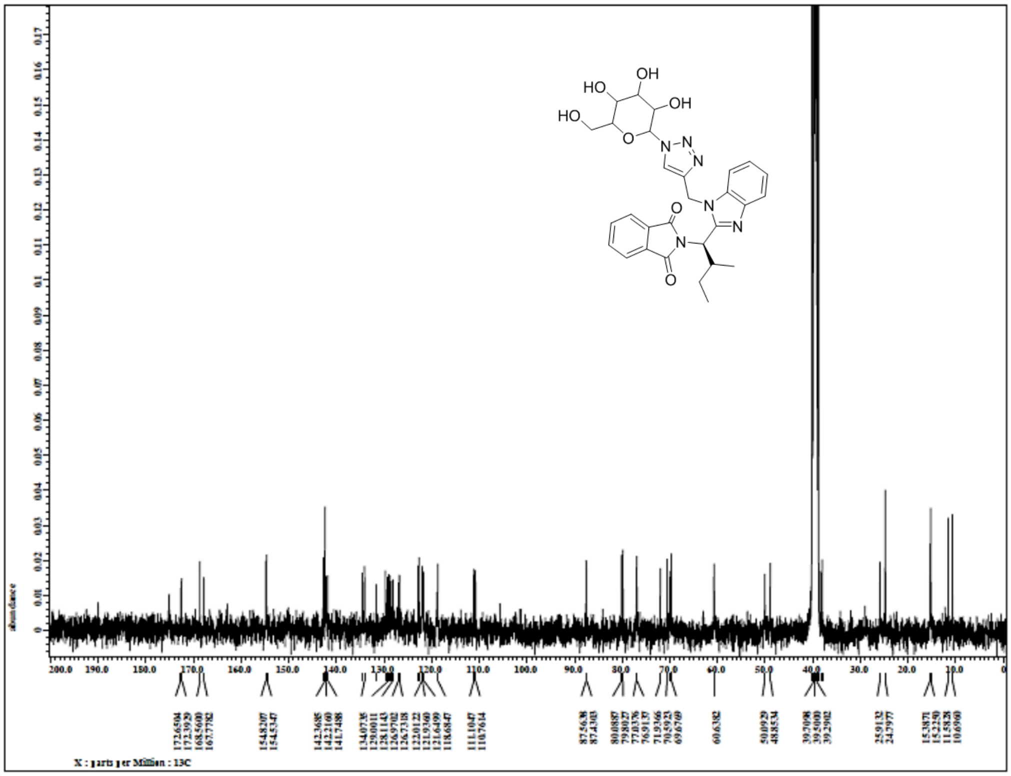

^{13}C NMR spectrum of compound **6y** (DMSO, 100MHz)

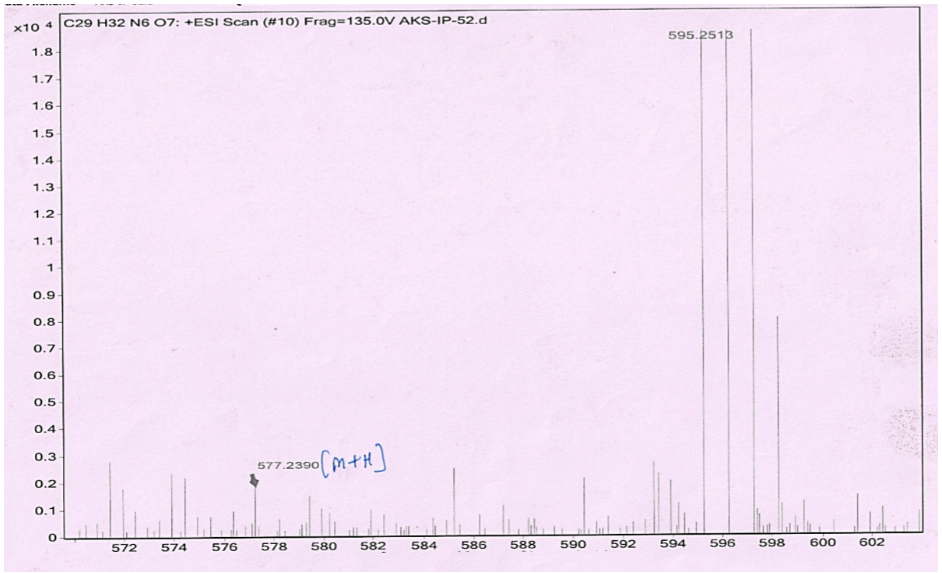

HRMS spectrum of compound **6y**

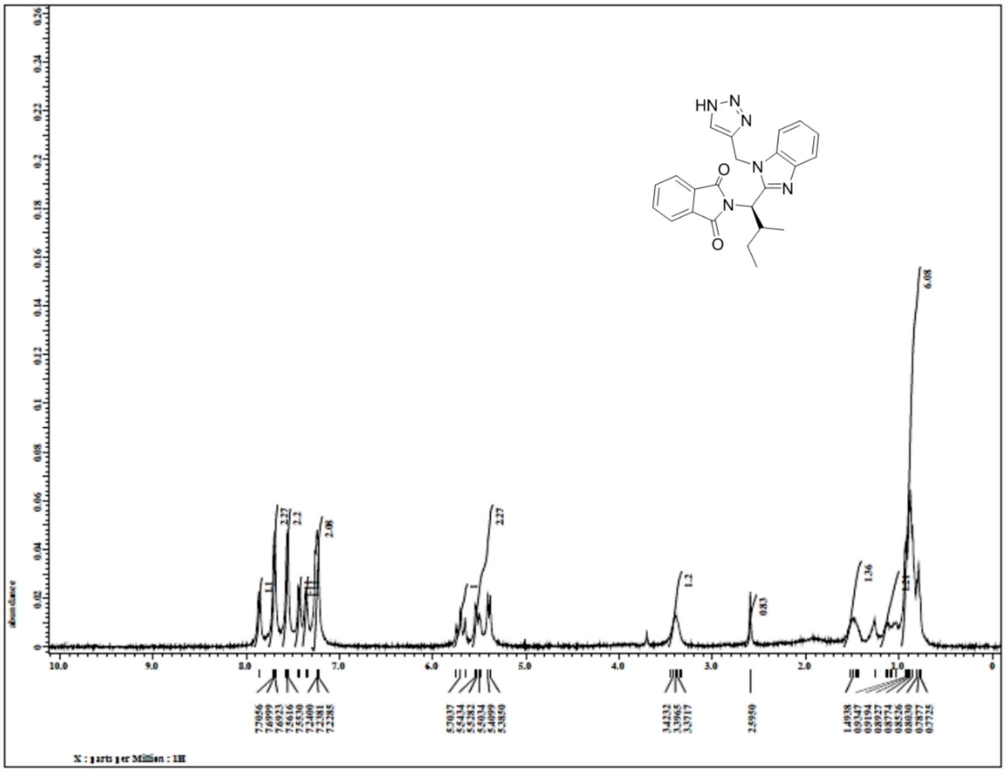

^{1}H NMR spectrum of compound **6z** ($CDCl_3$, 400MHz)

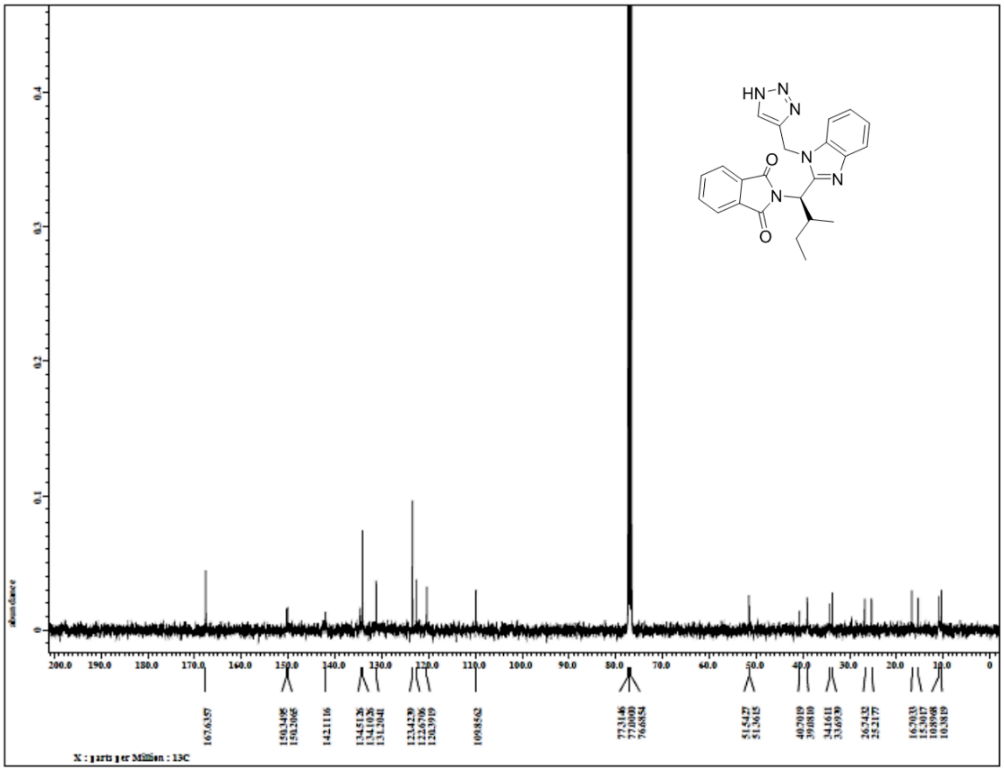

^{13}C NMR spectrum of compound **6z** ($CDCl_3$, 100MHz)

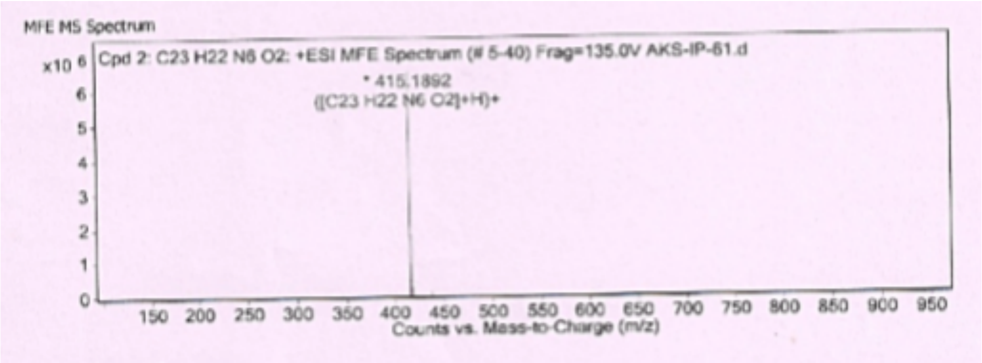

HRMS spectrum of compound **6z**

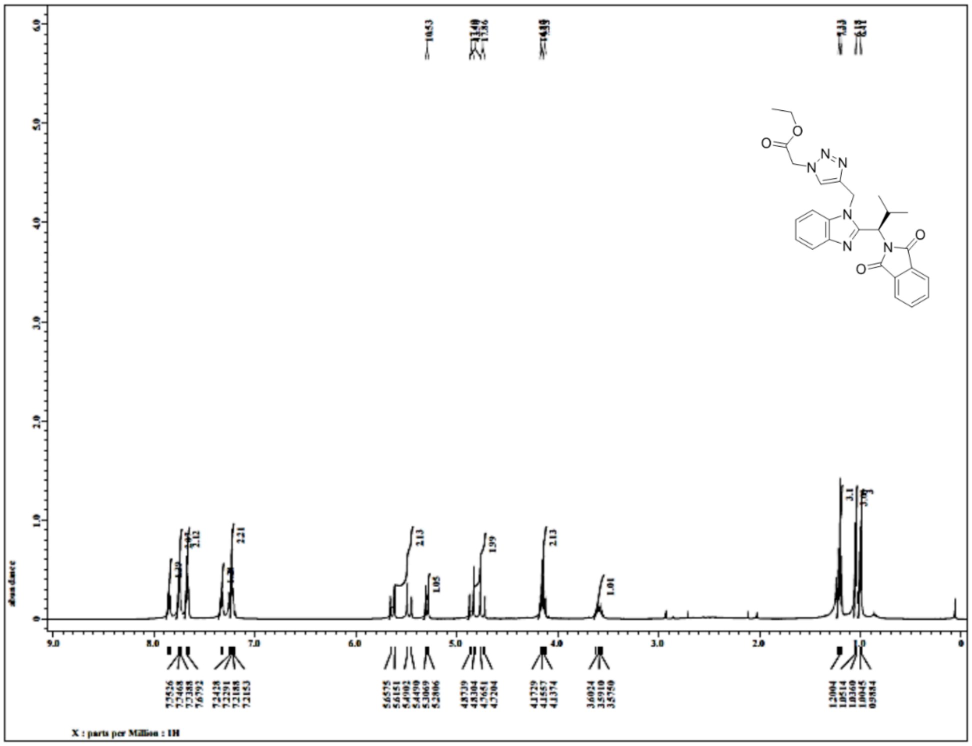

^{1}H NMR spectrum of compound **6a′** ($CDCl_3$, 400MHz)

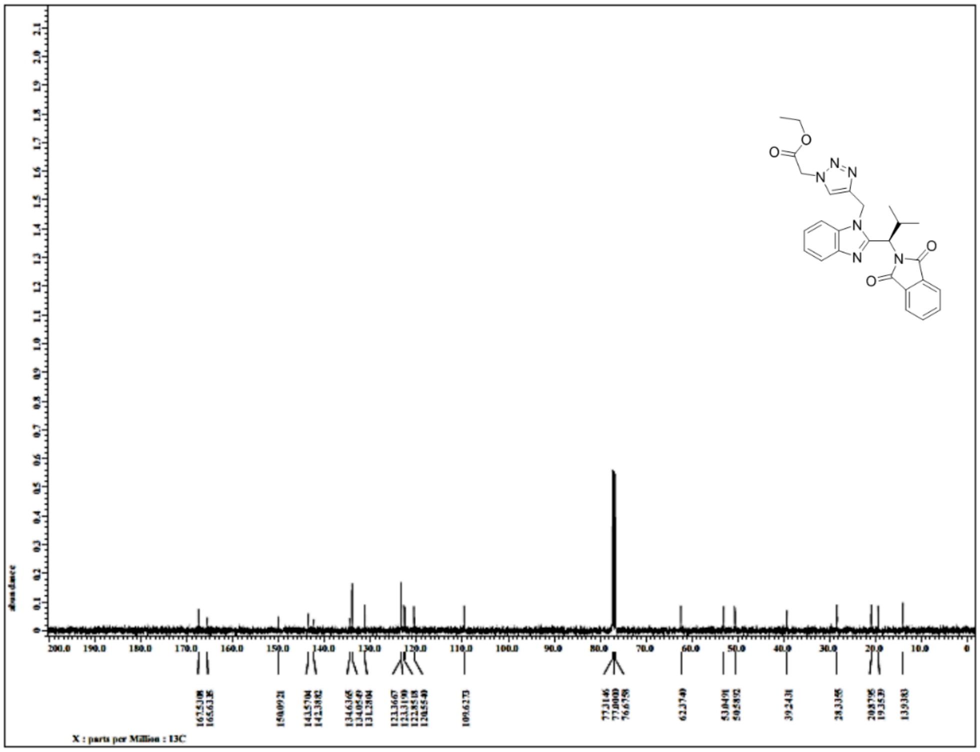

^{13}C NMR spectrum of compound **6a′** ($CDCl_3$, 100MHz)

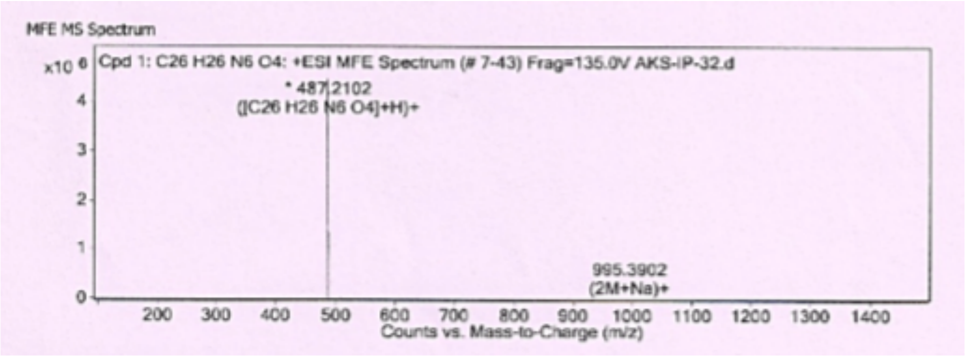

HRMS spectrum of compound **6a′**

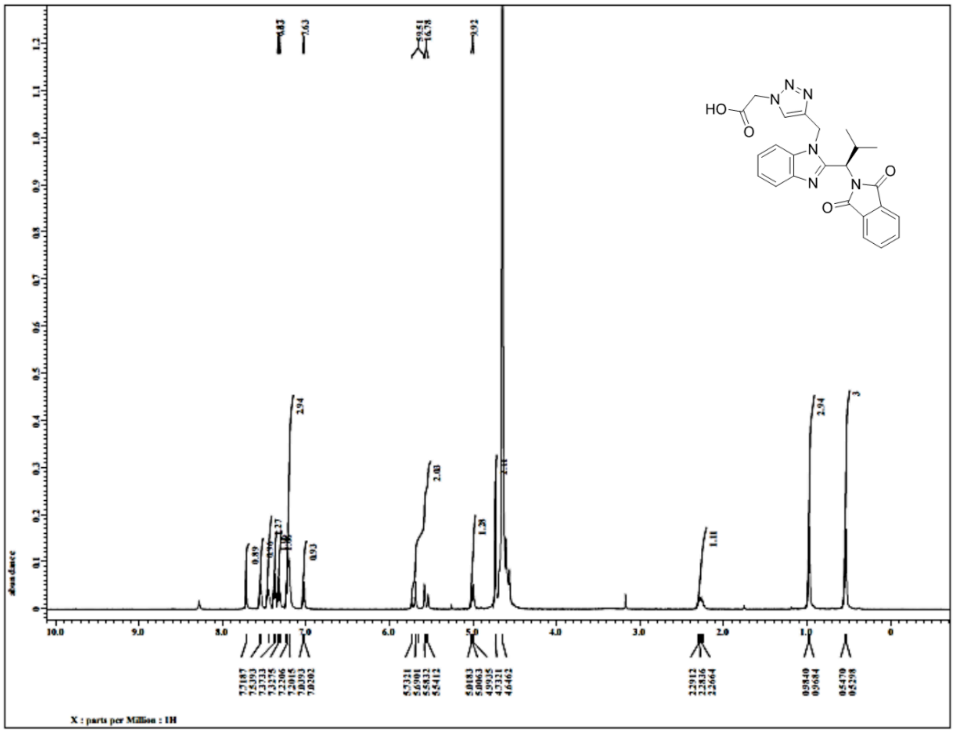

^{1}H NMR spectrum of compound **6b′** (D_2O, 400MHz)

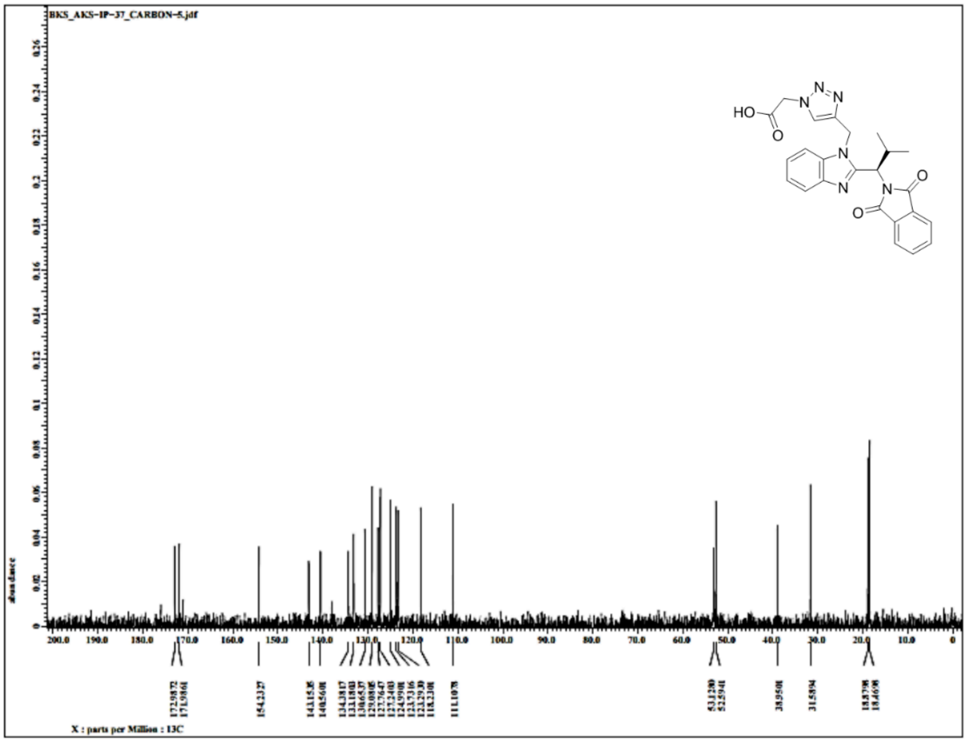

^{13}C NMR spectrum of compound **6b′** (D_2O, 100MHz)

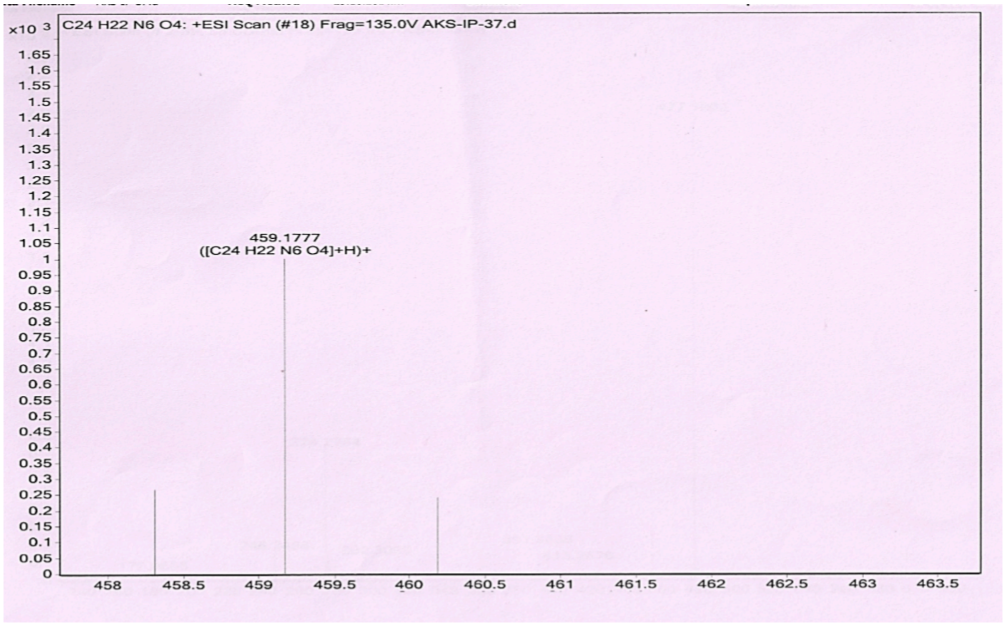

HRMS spectrum of compound **6b′**

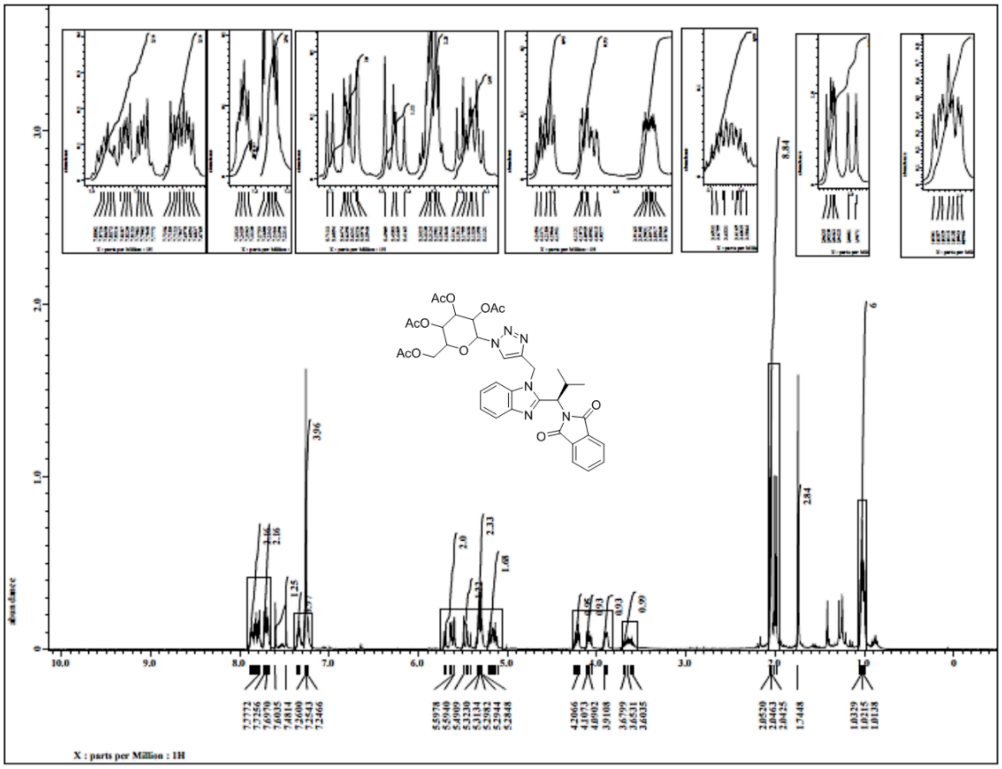

^{1}H NMR spectrum of compound **6c′** ($CDCl_3$, 400MHz)

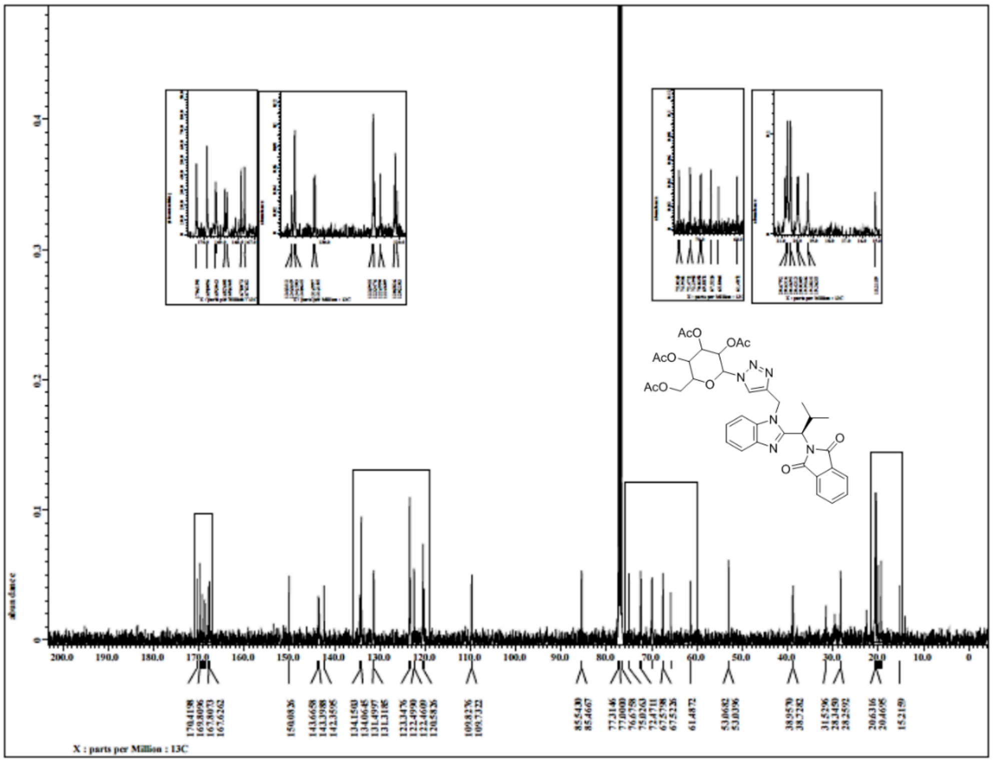

^{13}C NMR spectrum of compound **6c′** ($CDCl_3$, 100MHz)

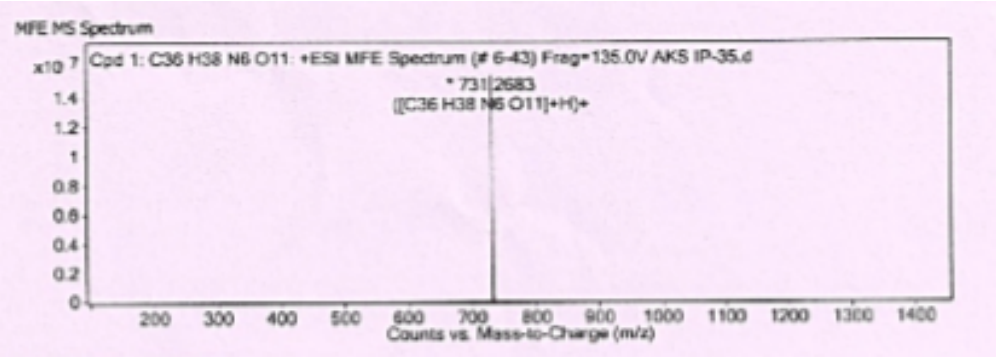

HRMS spectrum of compound **6c′**

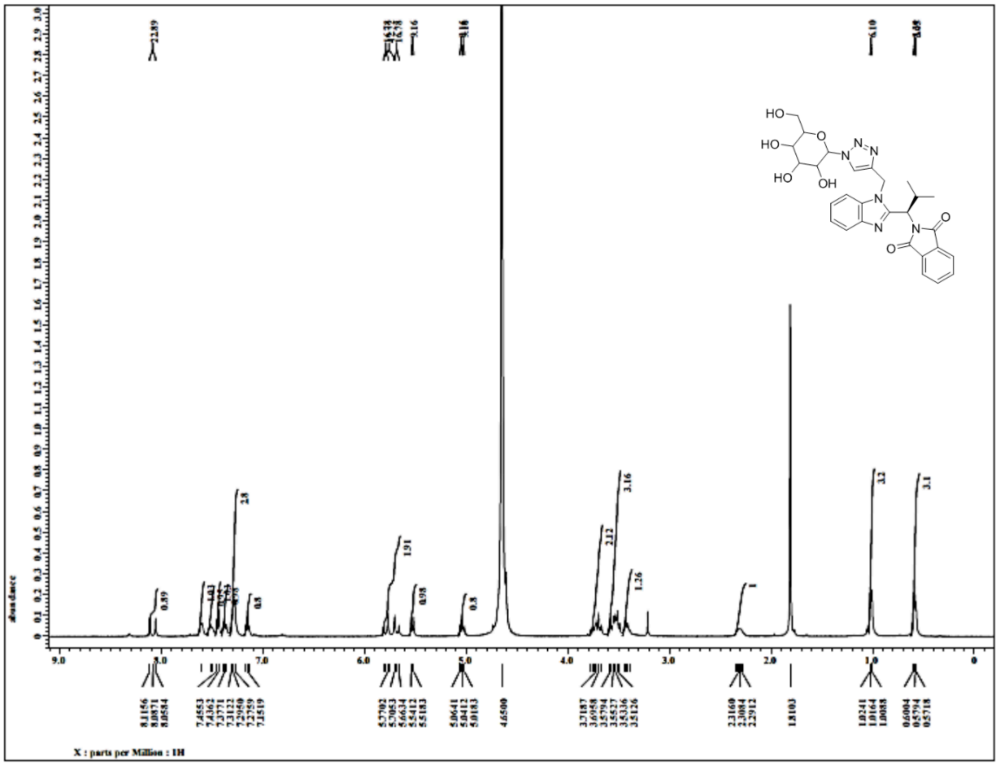

^{1}H NMR spectrum of compound **6d′** (D_2O, 400MHz)

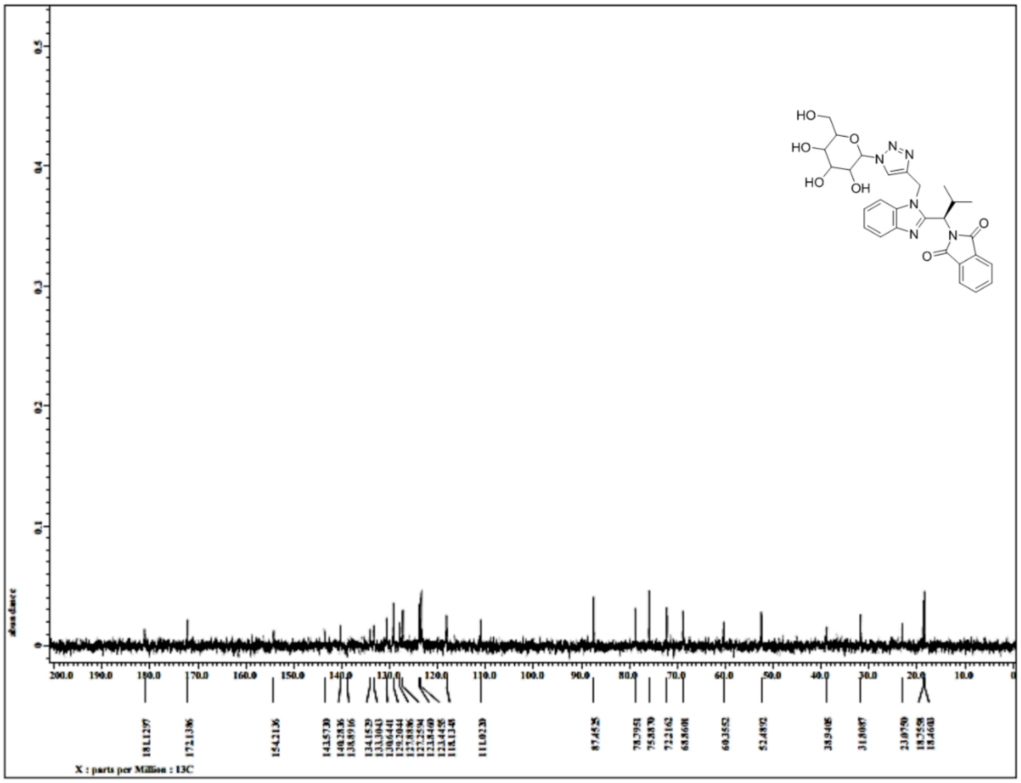

^{13}C NMR spectrum of compound **6d′** (D_2O, 100MHz)

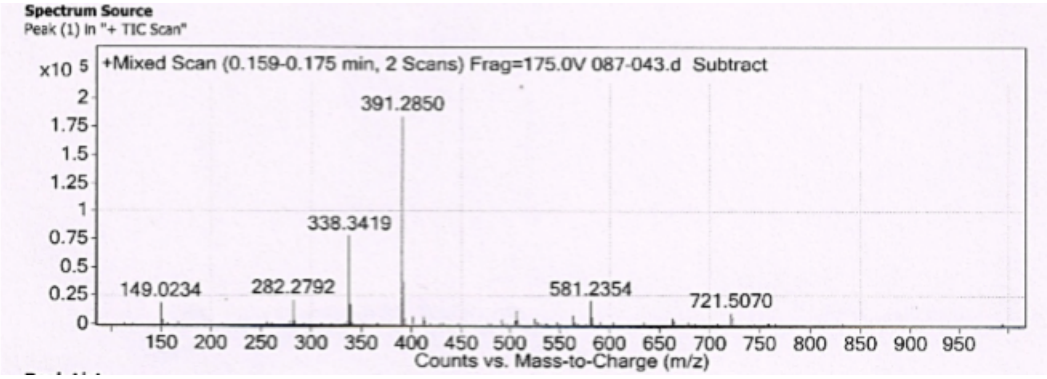

HRMS spectrum of compound **6d′**

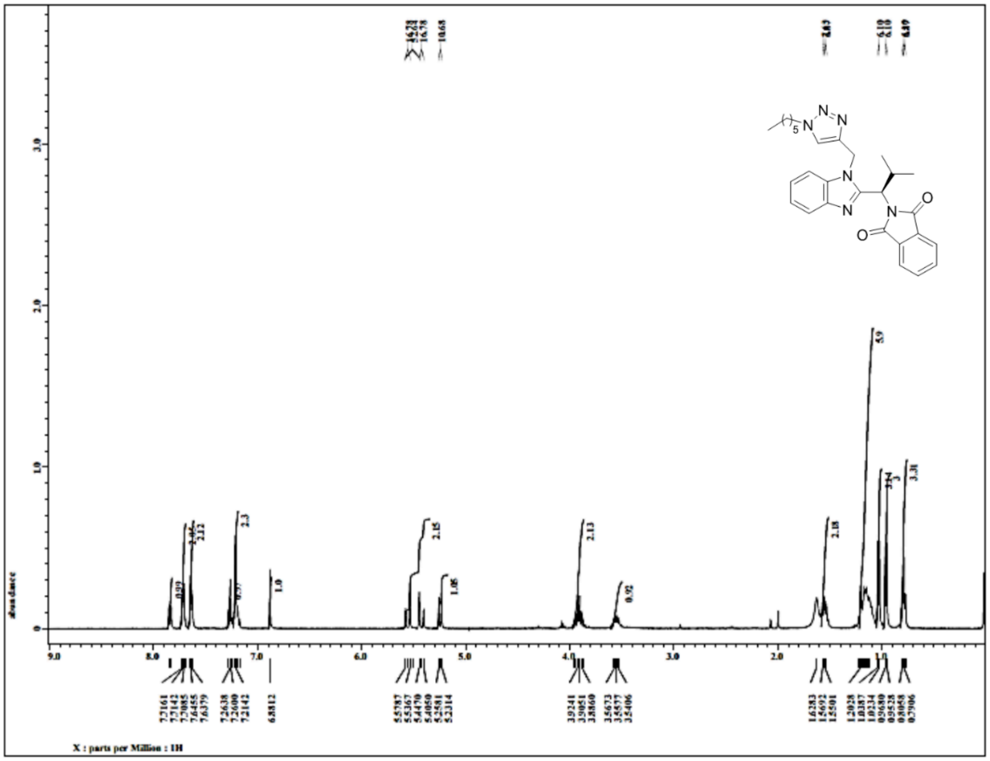

^{1}H NMR spectrum of compound **6e′** ($CDCl_3$, 400MHz)

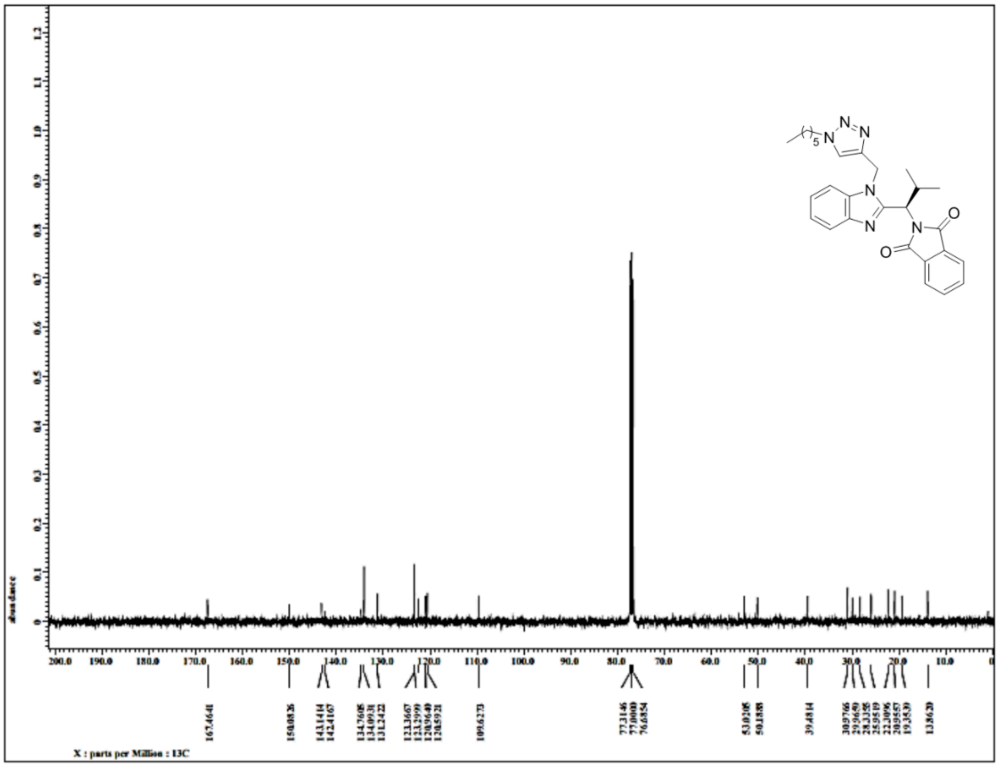

^{13}C NMR spectrum of compound **6e′** ($CDCl_3$, 100MHz)

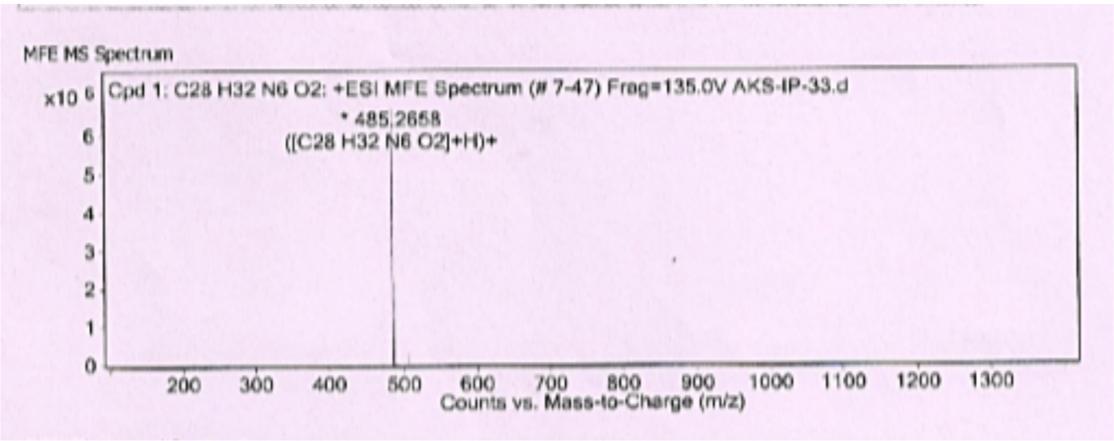

HRMS spectrum of compound **6e′**

References

[S1] *ENHANCE*: Oxford Xcalibur Single Crystal Diffractometer, version 1.171.34.40 (Oxford Diffraction Ltd: Oxford, U. K, **2006**).

[S2] *CrysAlisPro*, version 1.171.34.40 (Oxford Diffraction Ltd: Oxford, U. K, **2006**).

[S3] *SAINT-NT*, version 6.04, Bruker Analytical X-ray Systems (Madison, WI, **2001**).

[S4] Altomare, A. *et al.* SIR92 - a program for automatic solution of crystal structures by direct methods J. *Appl. Crystallogr.* **1994**, *27*, 435.

[S5] Sheldrick, G. M. *SHELXL-97*: Program for crystal structure refinement (University of Gottingen: Gottingen, Germany, **1997**).

[S6] Sheldrick, G. M. A short history of *SHELX Acta Crystallogr.* **2008**, *A64*, 112.

[S7] Farrugia, L. J. *WinGX* suite for small-molecule single-crystal crystallography *J. Appl. Crystallogr.* **1999**, *32*, 837.

[S8] Klaus, B. *DIAMOND*, version 2.0 c (University of Bonn, Bonn, Germany, **2004**).
